# Primary care and specialist physicians’ prescribing preferences for concurrent probiotic-antibiotic therapy: a multinational clinical practice survey across 13 countries

**DOI:** 10.3389/fmed.2025.1685840

**Published:** 2025-10-17

**Authors:** Stephan R. Vavricka, Cecilia Bartoli, Carolina Castillo

**Affiliations:** ^1^Department of Gastroenterology and Hepatology, University Hospital Zurich, Zurich, Switzerland; ^2^Department of Medical Affairs, Menarini, Florence, Italy

**Keywords:** microbiome, gut microbiota, probiotic supplementation, probiotic-antibiotic therapy, prescription preferences

## Abstract

Antibiotic-induced intestinal microbiota disruption represents a significant clinical concern, yet physician practices regarding concurrent probiotic supplementation remain poorly characterized across international healthcare systems. We investigated physician attitudes, knowledge, and prescribing behaviors in 390 physicians from 13 countries concerning probiotic-antibiotic co-administration and evaluated receptivity to innovative probiotic formulations with a cross-sectional online survey conducted in June 2025. Sampling included random selection of direct email outreach to known internal medicine specialists, general practitioners, and family physicians. Participants included internal medicine specialists (42%), general practitioners (46%), and family physicians (12%). There was an overall response rate of 52%. While 98% of physicians demonstrated high awareness of antibiotic-induced microbiota disruption, only 37% consistently recommended probiotics when prescribing antibiotics, with substantial variation in co-prescribing practices: 7% prescribed probiotics to nearly all patients, 13% to 1–10, 20% to 11–25, 33% to 26–50, and 27% to 51–99% of antibiotic recipients. Regional variations showed Lithuania, Colombia and Peru had the highest co-prescription rates (50% of physicians prescribing to >50% of patients), while Finland demonstrated more conservative patterns (50% prescribing to <25% of patients). During cold and flu season, antibiotic prescribing rates were evenly distributed across physician groups but decreased substantially outside peak respiratory illness periods. Most physicians (68%) found probiotics useful when taken with antibiotics, with 96% considering them for patients with prior antibiotic-associated diarrhea history. Physician receptivity to advanced probiotic formulations was consistently high, with 92% welcoming products that could be taken simultaneously with antibiotics and 92% willing to recommend such products to patients with special concerns, indicating that while physicians maintain high awareness of antibiotic microbiome impact, probiotic co-prescribing practices remain inconsistent globally with regional variations reflecting differences in healthcare policies and clinical guidelines.

## 1 Introduction

Antibiotics remain among the most frequently prescribed medications globally, with variations in prescribing patterns across healthcare systems. The recognition of antibiotic-induced intestinal microbiota disruption has intensified clinical interest in concurrent probiotic supplementation as a protective strategy. Existing evidence suggests that antibiotic therapy can significantly compromise intestinal microbial diversity, with recovery periods extending weeks to months following treatment completion. Recent systematic reviews have questioned the effectiveness of concurrent probiotic supplementation in maintaining gut microbiome diversity during antibiotic therapy, finding no significant differences in Shannon, Chao1, and observed operational taxonomic unit diversity indices between probiotic-supplemented and control groups (1). However, this lack of measurable diversity preservation does not negate the clinical utility of probiotics during antibiotic therapy.

However, current evidence supports probiotics in preventing antibiotic-associated diarrhea (AAD) (2–5), which affects up to 30% of patients receiving antibiotic therapy. Meta-analyses concluded that probiotics may provide a moderate effect for preventing AAD in children, adults, and elderly adults (5, 6). Thus, while probiotics may not preserve overall microbiome diversity metrics during antibiotic treatment, they provide clinically meaningful benefits through alternative mechanisms including immune modulation, increasing gut barrier integrity, producing antimicrobial substances, modulating the gut microbiome, increasing water absorption, and direct antimicrobial effects that collectively reduce AAD risk (7). Additionally, we must consider that strong evidence was found supporting the hypothesis that the efficacy of probiotics is both strain-specific and disease-specific, and clinical choice of the appropriate probiotic for each patient is challenging and requires both consideration of the type of probiotic strain(s) given and the type of disease indication for which it is needed (8).

Previous regional surveys have demonstrated considerable variability in probiotic co-prescription rates, ranging from 16% in Japan to 39% in Singapore among Asia-Pacific physicians, suggesting that cultural, economic, and healthcare system factors may influence physician decision-making regarding probiotic therapy (9, 10). Studies from Indonesia revealed that while physicians acknowledged the significance of antimicrobial resistance and rational antibiotic prescribing, this conflicted with reported suboptimal practices, including insufficient application of stewardship principles and defensive prescribing due to diagnostic uncertainty (9).

Several international studies have highlighted the variability in healthcare professionals’ knowledge, attitudes, and prescribing of probiotics. For example, Fijan et al. (11) found that while many health professionals across 30 countries rated their knowledge of probiotics as average to good, significant gaps remain, pointing to the need for better education and clearer guidelines. In the Asia-Pacific, Ghoshal et al. (12) reported low rates of probiotic co-prescription, and Zhang et al. (13) noted pediatric probiotic prescribing ranging from 13% in Japan to 60% in South Korea, which reflects diverse healthcare policies and cultural views. In the Middle East, surveys by Arshad et al. (14) and Ababneh *et al* (15) revealed poor knowledge and cautious attitudes among healthcare providers influenced by lack of focused training programs (14). European studies, including Wilson et al. (16), show general acceptance of probiotics and would recommend them if they had more information to support informed decision-making within patient care. In North America, Williams et al. (17) found positive but inconsistent attitudes among gastroenterologists, underscoring the need for more definitive evidence in practice. To date, no study has analyzed physician awareness, attitudes, and prescribing practices regarding this therapeutic approach across multiple countries spanning different continents, leaving a gap in understanding of global healthcare variations.

Given this context, we aimed to characterize physician attitudes, knowledge, and prescribing behaviors concerning concurrent probiotic-antibiotic therapy across a diverse international cohort. We also evaluated physician receptivity to innovative probiotic formulations and identifying barriers to probiotic co-prescription.

## 2 Methods

### 2.1 Design and participants

This multinational cross-sectional survey was conducted in June 2025 using established online survey methodology protocols. The study employed English-language questionnaires distributed through random selection of direct email outreach to known internal medicine specialists, general practitioners, and family physicians from Sermo, a global physician network comprising 1.3 million physicians across 150 countries. Eligible participants included practicing physicians spending ≥65% of their time in active patient care. The final sample comprised 390 physicians across 13 countries: European countries included Germany, Spain, Finland, France, Ireland, Italy, and Lithuania. Non-European representation included Colombia, Mexico, Peru, Saudi Arabia, South Africa, and United Arab Emirates. Sample size determination was based on descriptive study design requirements rather than statistical power calculations, as this investigation aimed to characterize physician attitudes and practices without conducting formal statistical comparisons between groups. The target of 30 physicians per country was selected to ensure adequate representation of prescribing patterns and attitudes within each healthcare system while maintaining feasibility for international data collection. Participants voluntarily agreed to participate in this market research study, and received payment for their participation.

### 2.2 Survey

The survey instrument underwent pre-testing with a small physician sample following standard Sermo platform methodology, with subsequent refinements made to question wording and survey flow based on pilot feedback. The questionnaire assessed multiple domains consistent with established physician survey methodology: physician demographics and practice characteristics, antibiotic prescribing patterns, microbiome awareness and knowledge, probiotic knowledge and attitudes, current probiotic co-prescribing practices, physician understanding of microbiome recovery, and evaluation of innovative probiotic products. Online survey administration was conducted through established platforms, following best practices for physician survey response optimization. Data collection procedures ensured participant anonymity and confidentiality.

### 2.3 Statistical analyses

Descriptive statistics characterized physician demographics and response patterns. Country-specific descriptions examined international variations in prescribing behaviors and product attitudes. Response frequencies were calculated for Likert-scale items assessing product importance ratings, with multi-point scales collapsed into binary categories (high versus low agreement) when appropriate.

Institutional Review Board or ethics committee approval was not required for this study as it constituted market research involving anonymous physician opinions and prescribing practices, falling outside the scope of human subjects research requiring formal ethical oversight.

## 3 Results

Each participating country contributed 30 physicians with a total of 390 physicians. The survey achieved representation across three primary medical specialties: general practice (46%), internal medicine (42%), and family practice (12%). There was an overall response rate of 52%. English proficiency distribution showed 66% of participants reporting very comfortable or fluent proficiency, with 34% reporting comfortable proficiency. Practice experience varied, with 23% having >20 years, 37% having 11–20 years, 30% having 6–10 years, and 10% having <5 years of experience.

### 3.1 Antibiotic prescribing patterns

The cold and flu season revealed differences in prescribing behaviors, with varying antibiotic prescription rates. During these peak respiratory illness months, prescribing practices showed a roughly even distribution: approximately 28% of physicians limited antibiotic prescriptions to fewer than 20% of their adult patients, while a similar proportion (27%) prescribed to 21–30% of cases. Moderately higher prescribing rates (40%) characterized 22% of physicians, while 23% demonstrated wider prescribing patterns, providing antibiotics to over 40% of their patients. Outside of cold and flu season these percentages varied substantially, with 25% of physicians limiting antibiotic prescriptions to <10% of the patients, 30% to 11–20% of cases, 22% to 21–30% of cases, 13% to 31–40% of cases, and only 10% to over 40% of cases.

### 3.2 Microbiome awareness and knowledge

**Figure 1:**
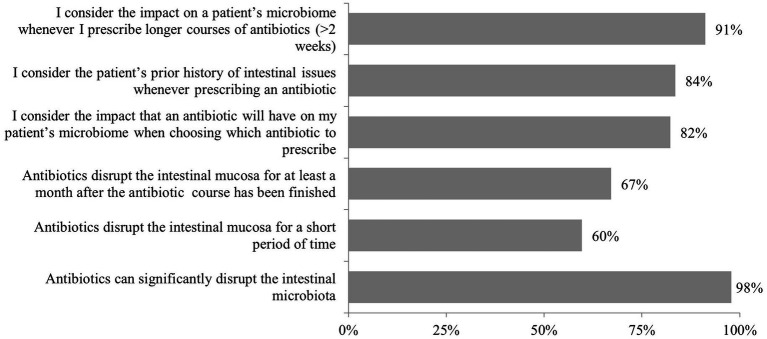
Responses to the question “Thinking about antibiotics and the impact they can have on the microbiome, how would you respond to the following statements?.” Highlights: *n* = 390 total; *n* = 30 per country. 98% of physicians recognized the risk of antibiotic-induced microbiota disruption; 91% of physicians reported routinely considering the impact of antibiotics on the gut microbiome when making prescribing decisions; 67% indicated microbiome disruption persists for ≥1 month post-antibiotic; 60% acknowledged shorter-term mucosal effects.

Physicians demonstrated consistent awareness of antibiotics’ effects on intestinal microbial communities, with 98% recognizing antibiotic-induced microbiota disruption and most actively incorporating this knowledge into clinical decision-making (82% weigh microbiome impact when selecting antibiotics, 84% consider patients’ previous intestinal complications). Regarding recovery timeframes, 60% acknowledged that mucosal disruption occurs over shorter timeframes, while 67% recognized that meaningful microbiome perturbations persist for at least 1 month following treatment completion as shown in Figure 1. The influence of microbiome science became most pronounced when physicians contemplated extended antibiotic courses exceeding 2 weeks, with 91% of practitioners considering these ecological consequences into their treatment planning (Figure 1).

### 3.3 Probiotic knowledge and attitudes

Despite acknowledging practical challenges with probiotic co-administration (52% recognized deactivation by antibiotics, 48% acknowledged the dosing complexity), physicians showed strong support for probiotic utility. 68% found probiotics useful when taken with antibiotics, with particularly high endorsement for specific high-risk clinical scenarios: 96% for patients with prior AAD, 92% for prolonged antibiotic courses, and 87% for broad-spectrum or powerful antibiotics (noting that the term “powerful antibiotics” may have been subject to varied interpretation among respondents). 92% welcomed new probiotic options that could be taken alongside antibiotics to reduce microbiome impact and accelerate recovery of a diverse microbiota.

### 3.4 Current probiotic co-prescribing practices

**Figure 2:**
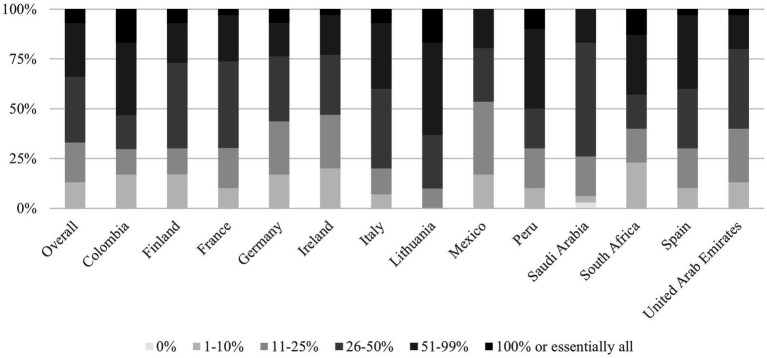
Responses by country to the question: “When prescribing antibiotics to adult patients today, for what percentage of patients do you also recommend probiotics?.” Highlights: *n* = 390 total; *n* = 30 per country. Prescribing rates ranged broadly, 7% prescribe probiotics to nearly all patients, 27% to 51–99%, revealing heterogeneity across countries. Lithuania, Colombia and Peru: Highest co-prescription rates (≥50% of physicians prescribe to >50% of patients). Finland: Most conservative (50% prescribe to <25% of patients).

Among surveyed physicians, 7% indicated they prescribe probiotics to every patient receiving antibiotic therapy. The distribution showed: 13% prescribed probiotics to 1–10% of patients, 20% to 11–25, 33% to 26–50, and 27% to 51–99% of patients. All physicians in the study reported having prescribed probiotics to adult patients receiving antibiotic therapy at some point, as shown in Figure 2.

### 3.5 Physician understanding of microbiome recovery

When presented with a graph illustrating ciprofloxacin’s impact on intestinal microbiome adapted from Dethlefsen et al. (18), physician responses demonstrated high understanding of dysbiosis timeline and recovery requirements. The majority (89%) recognized that antibiotic-induced dysbiosis occurs rapidly after treatment initiation, and 89% acknowledged that weeks to months are required for microbiome diversity recovery. Regarding concurrent probiotic therapy, 89% agreed that it may be prudent to recommend a probiotic alongside the antibiotic to lessen the impact of antibiotic-induced dysbiosis. However, opinions diverged on optimal continuation duration to allow full recovery of the microbiome: 60% supported continuing probiotics for one additional week post-antibiotic therapy, 57% for one additional month, and 41% for three additional months.

### 3.6 Product profile evaluation

**Figure 3:**
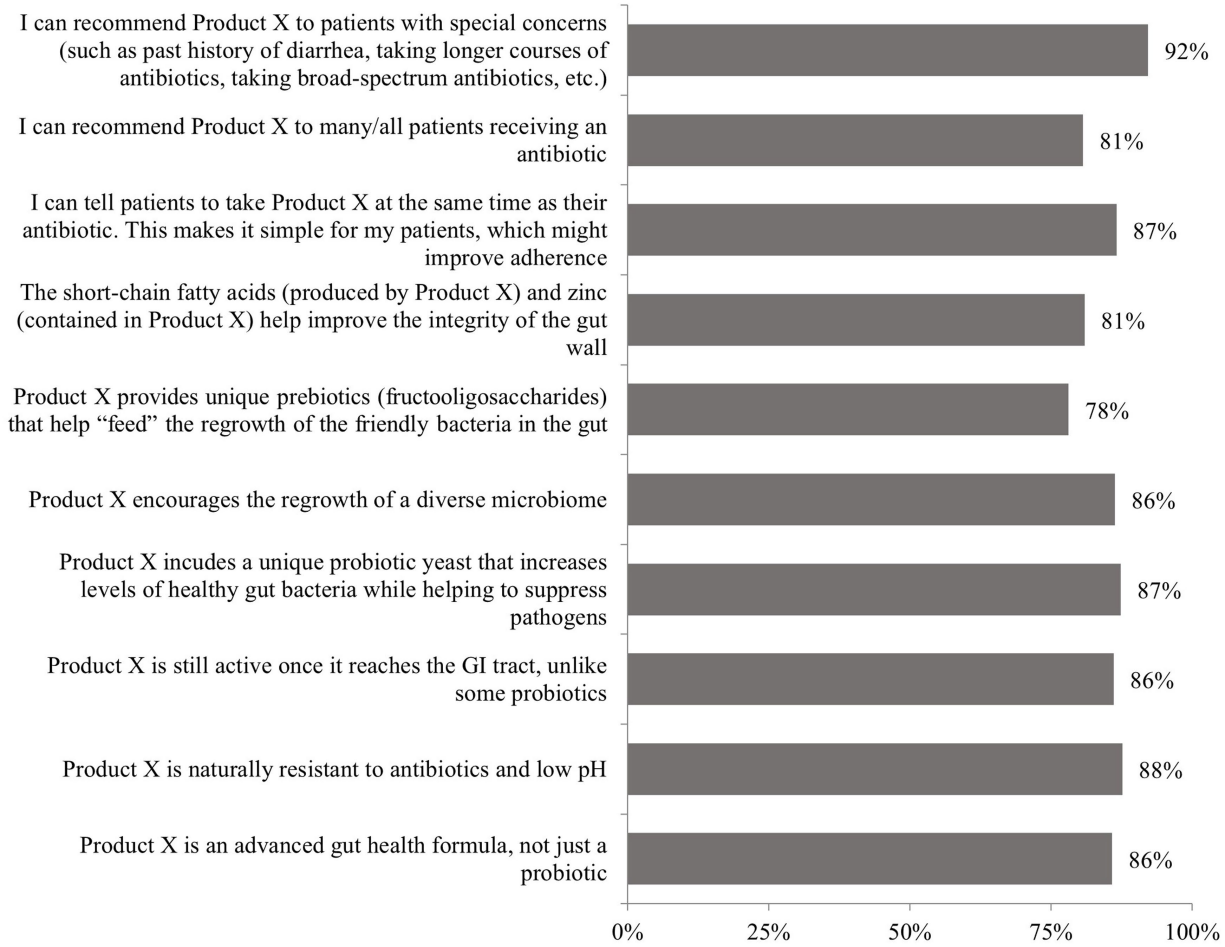
Responses to the question: “Based on this brief product profile, how would you respond to the following statements?.” GI, gastrointestinal; Product X, 3biotic+. Highlights: The greatest agreement (92%) was for recommending Product X to patients with special concerns, such as those with a history of diarrhea or requiring longer or broad-spectrum antibiotic courses; 87% agreed they could recommend Product X for concurrent use with antibiotics, and appreciated the simplicity of taking it at the same time as antibiotic therapy.

Physician receptivity to the probiotic formulation (Kaleidon 3biotic+) was consistently high across all evaluated characteristics. Key acceptance rates included: 92% would recommend it to patients with special concerns (such as past history of diarrhea, taking longer courses of antibiotics, taking broad-spectrum antibiotics, etc.), 88% acknowledged natural antibiotic resistance and low pH resistance, 86% viewed it as an advanced gut health formula rather than a simple probiotic, 86% recognized continued gastrointestinal tract activity, and 87% appreciated the unique probiotic yeast component, as presented in Figure 3.

### 3.7 Regional variations

Finnish physicians demonstrated more conservative approaches, with 73% prescribing antibiotics to only 11–20% of patients during cold season, escalating to 77% limiting prescriptions to fewer than 10% of patients during non-seasonal periods. In contrast, Spanish practitioners exhibited substantially wider prescribing behaviors, with over half (53%) providing antibiotics to more than 40% of their patients during cold season.

Regional variations were observed regarding probiotic co-prescription practices. Lithuania, Colombia and Peru demonstrated the highest co-prescription rates, with 64, 54 and 50% of physicians prescribing probiotics to >50% of antibiotic recipients and 27, 17 and 20% to 26–50%, respectively. Mexico and Ireland showed the most conservative pattern, with 54 and 47% prescribing to less than 25% patients, and no physician prescribing it to all the patients, respectively (Figure 2). Countries like Germany or Finland, prescribed probiotics to less than 50% of the patients in 76 and 73% of the occasions, respectively.

Country-specific data presented in Supplementary data revealed consistently high acceptance of Kaleidon 3biotic + across all participating nations, with minimal variation in product perception.

## 4 Discussion

This international survey reveals some disconnection between physician awareness of antibiotic microbiome impact and actual probiotic co-prescribing practices among outpatient settings globally. Despite 98% of physicians recognizing antibiotic-induced microbiota disruption, only 37% consistently recommended probiotics when prescribing antibiotics. The observed regional variations in prescribing practices likely reflect differences in healthcare system policies, and cultural attitudes toward probiotic therapy. Lithuania, Colombia and Peru showed the highest co-prescription rates (with 50% of physicians prescribing probiotics to over half their antibiotic patients), while countries such as Mexico or Ireland showed a more conservative pattern. The observed patterns suggest that physician decision-making is influenced by multiple factors including regional medical education and cultural attitudes toward preventive interventions.

Prescribing patterns observed across countries reflect documented regional variations in antimicrobial philosophy and align with previously reported differences in healthcare professional knowledge and attitudes toward probiotics (11–16). The restrained approach evident in Finland aligns with the broader Nordic tradition of antimicrobial stewardship (19, 20). These national approaches to antimicrobial stewardship have been shaped by decades of policy development, surveillance programs, and professional education that emphasize restrictive use of antimicrobials (20). Our data are in alignment with these differences, which extend beyond antibiotic prescribing including probiotic co-therapy, where cultural attitudes toward preventive interventions and supplement use may influence clinical decision-making consistent across international boundaries. Although implementation challenges may vary across healthcare systems, evidence-based stewardship principles should serve as the universal standard, transcending regional differences and ensuring optimal patient care and antimicrobial conservation. Regional differences may partly reflect healthcare system factors such as reimbursement policies, availability of probiotic formulations, and local guideline inclusion.

The awareness of the microbiome’s importance among physicians across thirteen countries demonstrates how microbiome science has become a core part of medical education and ongoing training worldwide. This shared understanding cuts across different healthcare systems and training backgrounds, suggesting that the basic principles of how antibiotics affect the microbiome are now broadly accepted in the medical community. Yet, turning this knowledge into everyday practice remains complex, with many barriers to implementation. Clinical guidelines serve as essential legal and professional frameworks that empower physicians to implement evidence-based prevention and treatment strategies with confidence. These frameworks prioritize patient safety and optimal care rather than unguided clinical experimentation. These authoritative tools establish clear pathways that enable clinicians to act decisively while ensuring standardized clinical practice. However, the limited inclusion of probiotics in current formal guidelines primarily reflects ongoing scientific evaluation and the requirement for definitive evidence across diverse clinical scenarios, rather than administrative oversight. Existing guidance from leading health authorities remains inconsistent, while many acknowledge that probiotics can lower the risk of AAD, few offer firm recommendations for routine use. Furthermore, variation in probiotic product quality and formulation remain another major practical barrier, with physicians citing inconsistent composition, viability, and dosing among commercial brands. The lack of regulatory harmonization (21) and clear labeling (22) further complicates prescribing decisions, reflecting ongoing uncertainty about the robustness and generalizability of available evidence (23, 24).

Over 80% of physicians report the impact on the microbiome when choosing antibiotics and assessing patients, showing that scientific insights do influence clinical reasoning. The real challenge is not awareness but creating clear, evidence-based guidelines that help translate understanding into consistent action. Focusing on high-risk patient groups offers a practical way to improve outcomes, aligning with current evidence on preventing AAD, while also considering the resource limitations and diverse healthcare environments around the world.

This study has several methodological limitations that warrant consideration. The investigation employed convenience sampling through a single platform, potentially introducing selection bias toward physicians which may be more engaged, digitally literate, and research-oriented than the average clinician. The English-language requirement may have excluded physicians with limited proficiency, potentially affecting generalizability to non-English speaking healthcare environments and reducing the representativeness of the sample within participating countries. Self-reported prescribing data may be subject to recall bias and social desirability effects, potentially overestimating adherence to evidence-based practices, as documented in similar physician survey studies. Recall bias may particularly affect physicians’ ability to accurately estimate the percentage of patients for whom they prescribe probiotics alongside antibiotics, as these decisions may vary considerably based on individual patient factors that are not systematically documented in clinical practice. The cross-sectional design prevents assessment of causality between awareness and prescribing behaviors, limiting our ability to establish temporal relationships between knowledge and practice patterns. Although the survey found that a majority of physicians expressed strong interest in advanced probiotic formulations such as Kaleidon 3biotic+, it is important to recognize that these responses may reflect hypothetical optimism rather than guaranteed real-world prescribing behavior. When new products are introduced in a survey context, physicians’ stated receptivity often exceeds actual adoption rates observed in practice. We also recognize that these findings may have limited generalizability to specialists such as infectious diseases physicians or those practicing in inpatient hospital settings, where prescribing habits and perspectives on probiotic therapies could differ markedly from those of primary care and general practice physicians. Additionally, the survey did not capture detailed information about specific clinical scenarios, patient populations, or institutional guidelines that may influence prescribing decisions, potentially oversimplifying the complex factors that guide clinical practice. However, this study demonstrates significant strengths that enhance its validity and impact. The large sample size of 390 physicians across 13 diverse countries provides a broad representation and enables a cross-national description of physician attitudes and practices. The standardized questionnaire design ensures consistency in data collection across multiple healthcare systems and cultural contexts, facilitating reliable international comparisons. The multinational scope spanning European, Middle Eastern, African, and South American countries offers valuable insights into global variations in clinical practice that extend beyond single-country or regional studies.

## 5 Conclusion

This international survey demonstrates high physician awareness of antibiotic microbiome impact but inconsistent translation into probiotic co-prescribing practices. The regional variations suggest that healthcare system policies and local guidelines significantly influence clinical decision-making. The overwhelming physician interest in advanced probiotic formulations that can be administered simultaneously with antibiotics suggests that product innovation and clearer clinical guidance may jointly help overcome barriers to probiotic co-prescription. Future research should focus on implementation strategies that bridge the gap between physician awareness and consistent evidence-based practice.

## Data Availability

The original contributions presented in the study are included in the article/Supplementary material, further inquiries can be directed to the corresponding author.
