# Supplementary material for: Primary care and specialist physicians’ prescribing preferences for concurrent probiotic-antibiotic therapy: a multinational clinical practice survey across 13 countries
Source: Front Med (Lausanne). 2025 Oct 17;12:1685840. doi: 10.3389/fmed.2025.1685840 (PMC12577560; doi:10.3389/fmed.2025.1685840)
Supplement: Supplementary file 1 [file Data_Sheet_1.docx]

Supplementary Material

Table of contents

[1 Overall Report 2](#_Toc206070665)

[2 Colombia Report 7](#_Toc206070666)

[3 Finland Report 11](#_Toc206070667)

[4 France Report 15](#_Toc206070668)

[5 Germany Report 19](#_Toc206070669)

[6 Ireland Report 23](#_Toc206070670)

[7 Italy Report 27](#_Toc206070671)

[8 Lithuania Report 31](#_Toc206070672)

[9 Mexico Report 35](#_Toc206070673)

[10 Peru Report 39](#_Toc206070674)

[11 Saudi Arabia Report 43](#_Toc206070675)

[12 South Africa Report 47](#_Toc206070676)

[13 Spain Report 51](#_Toc206070677)

[14 United Arab Emirates Report 55](#_Toc206070678)

# Overall Report

In a typical week during cold and flu season, what percentage of the adult patients you see receive an antibiotic?


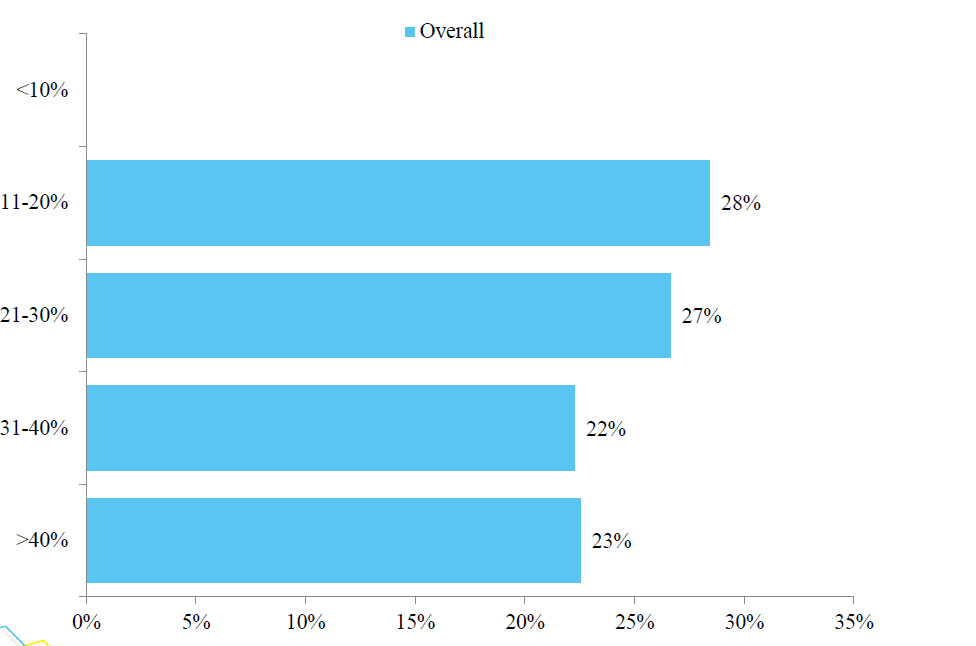


In a typical week, not during cold and flu season, what percentage of the adult patients you see receive an antibiotic?


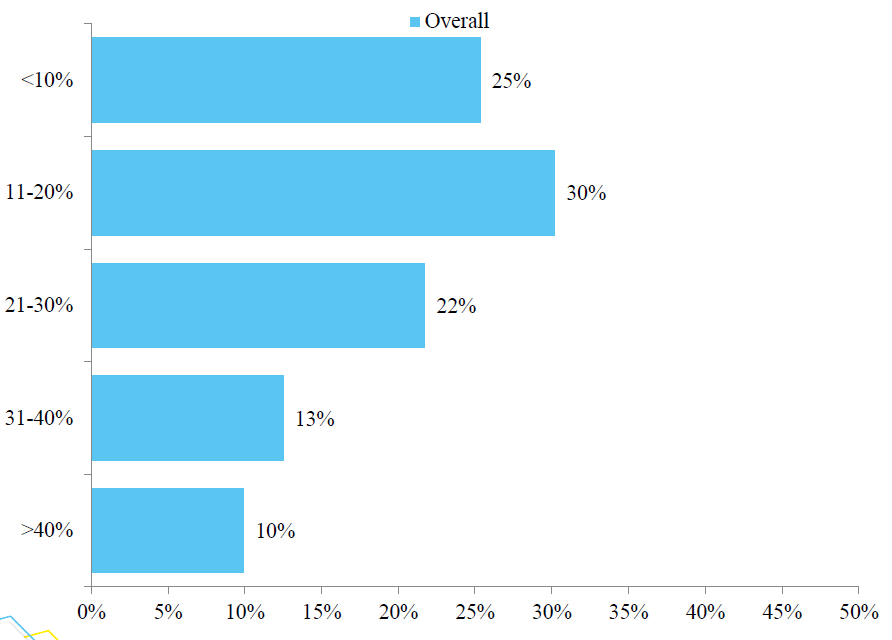


Do you see a role for probiotics when prescribing antibiotics to adult patients?


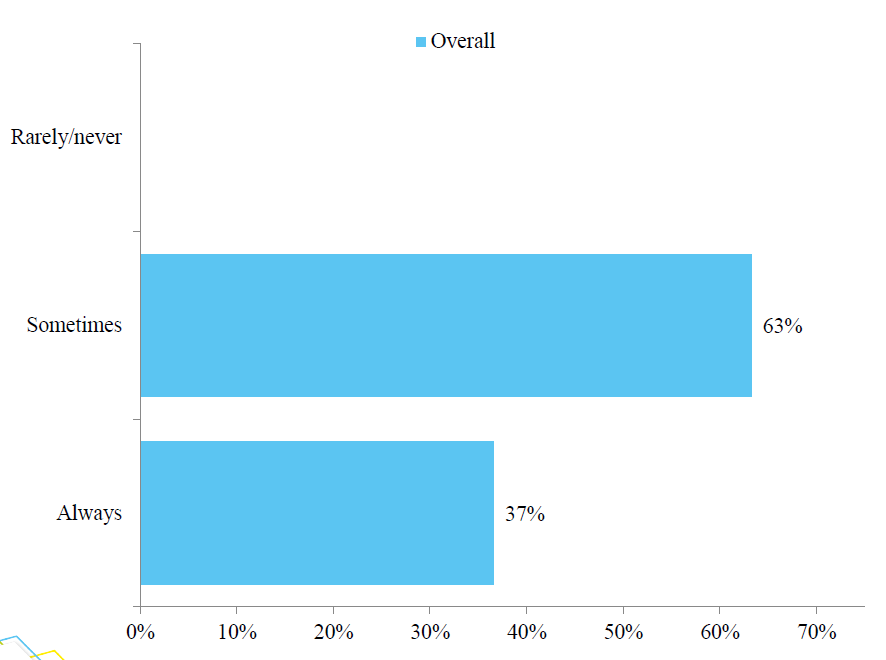


Thinking about antibiotics and the impact they can have on the microbiome, how would you respond to the following statements?


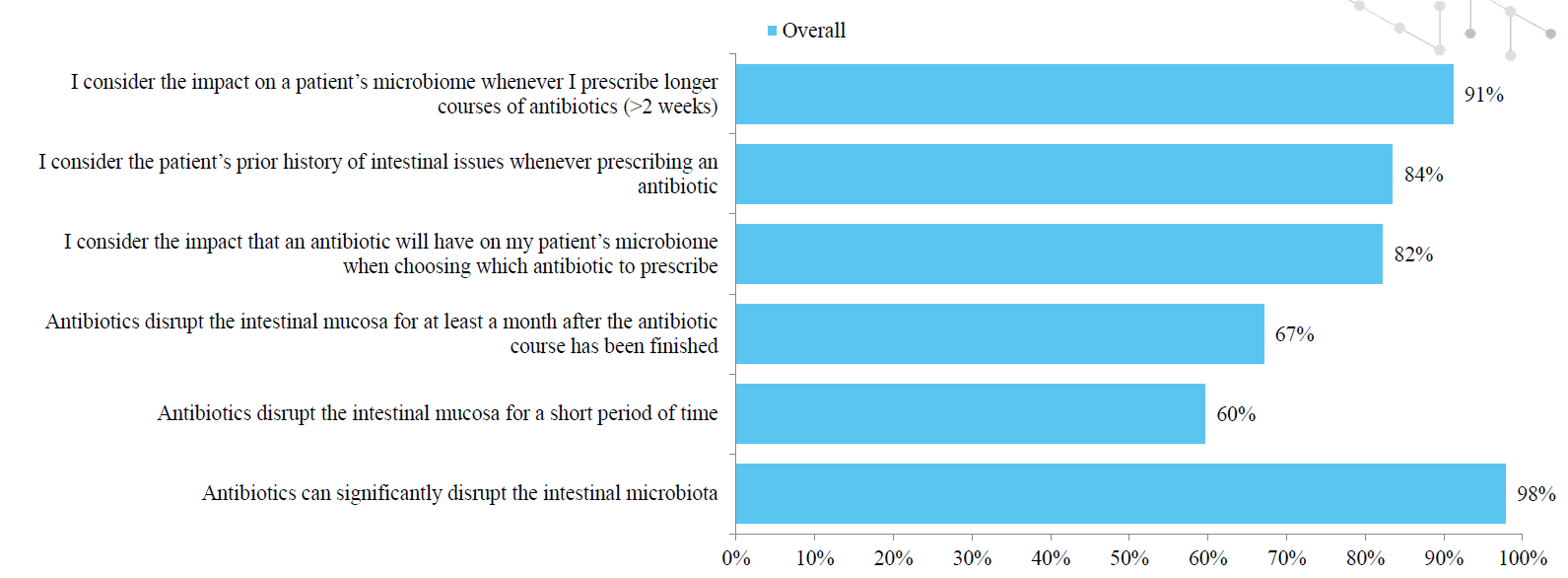


Thinking about antibiotics and the impact they can have on the microbiome, how do you respond to the following statements about probiotics?


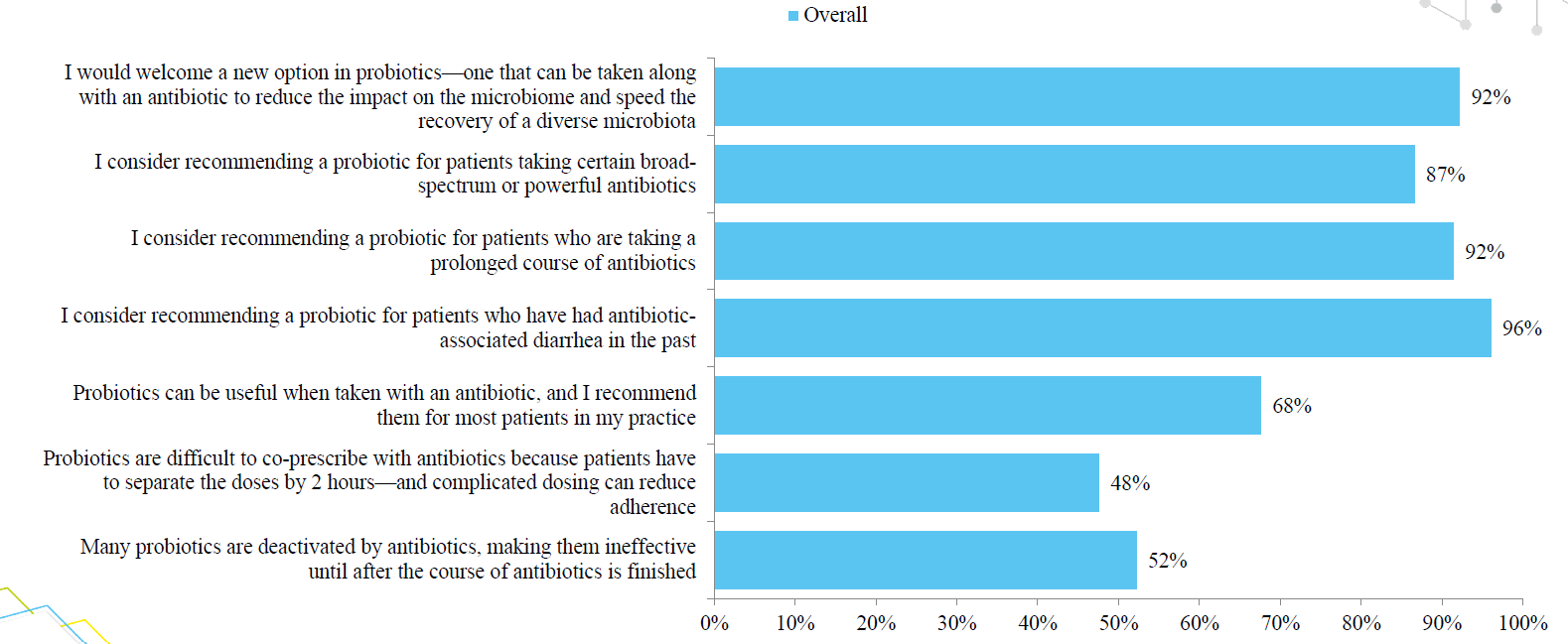


When prescribing antibiotics to adult patients today, for what percentage of patients do you also recommend probiotics?


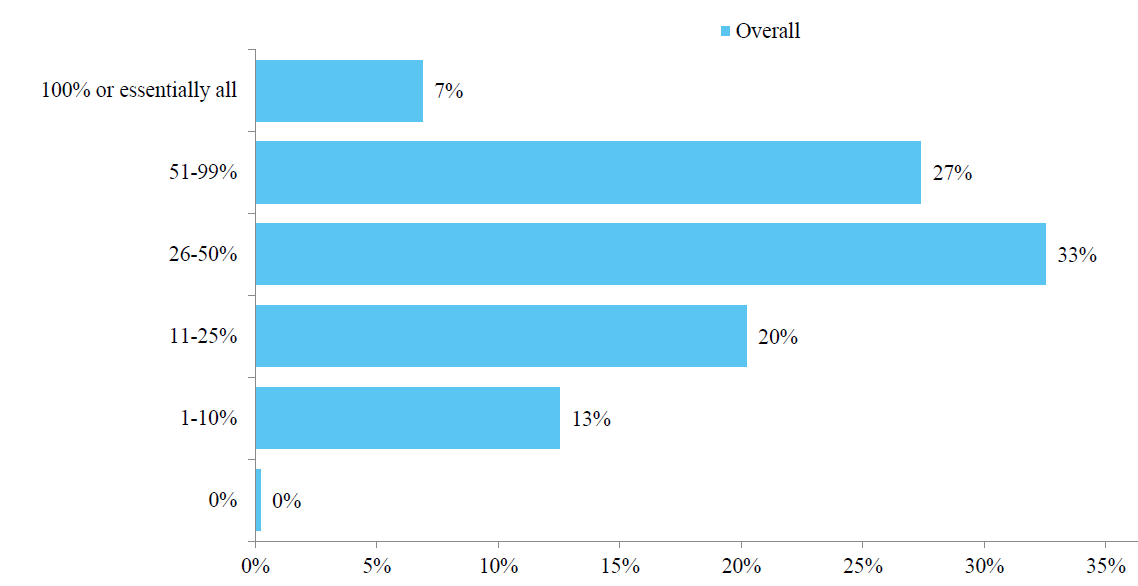


How would you respond to the following statements?


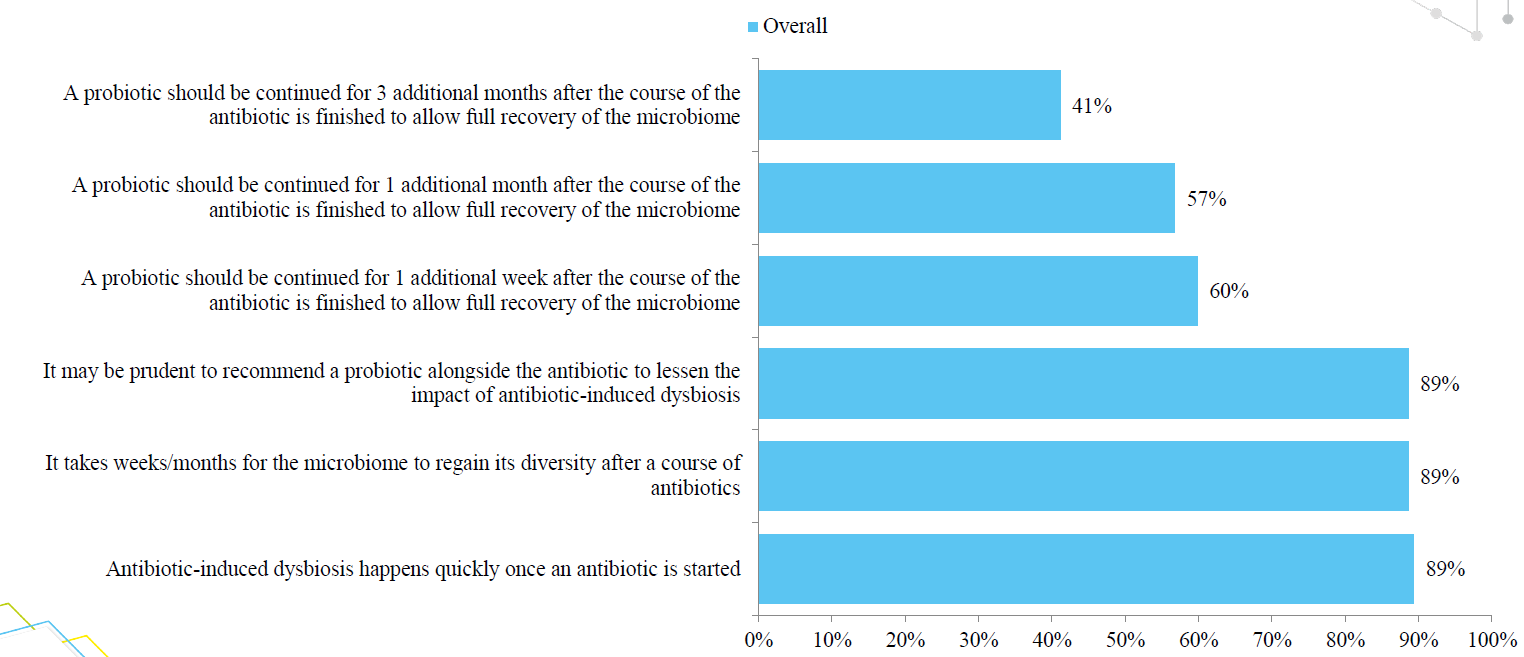


How would you respond to the following statements?


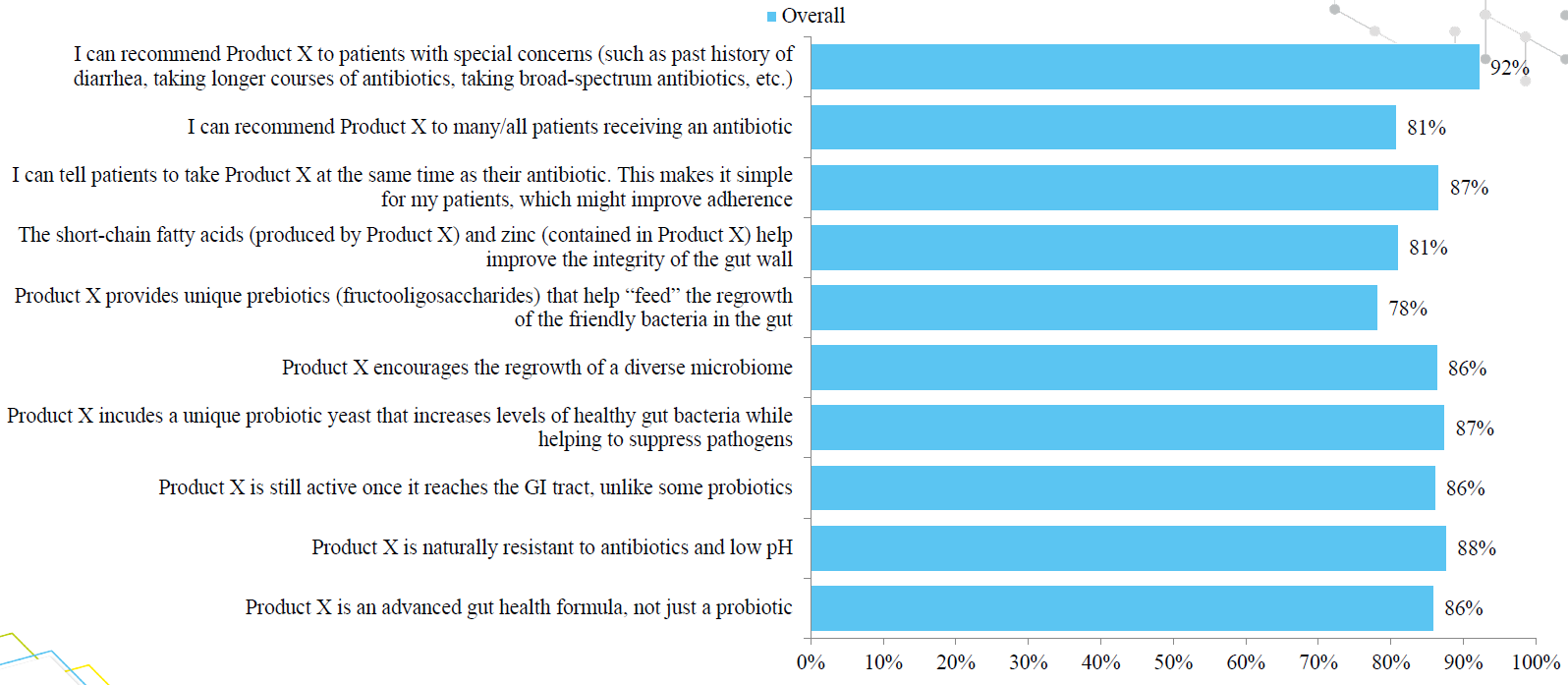


# Colombia Report

In a typical week during cold and flu season, what percentage of the adult patients you see receive an antibiotic?


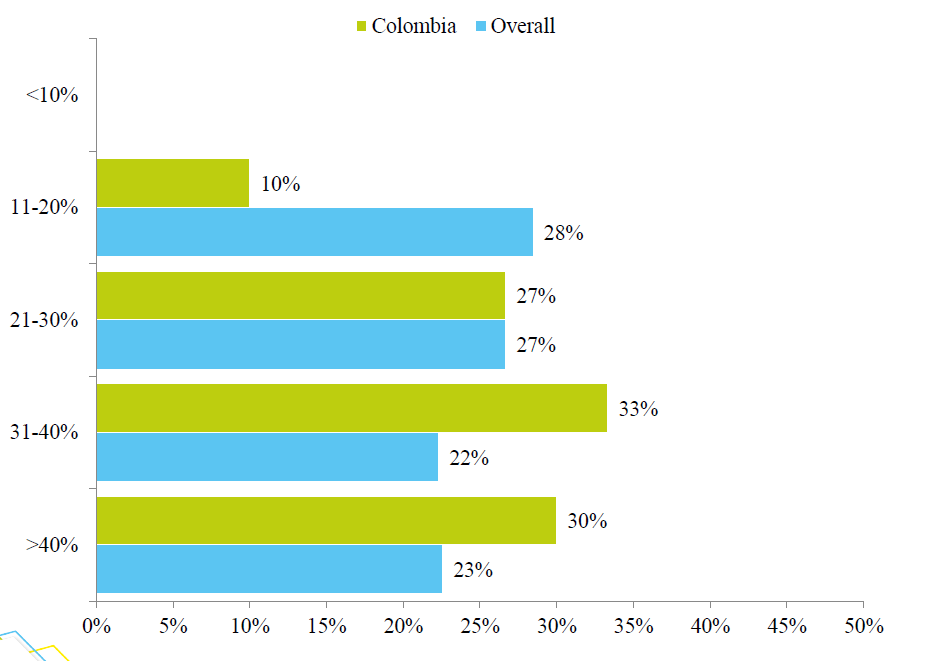


In a typical week, not during cold and flu season, what percentage of the adult patients you see receive an antibiotic?


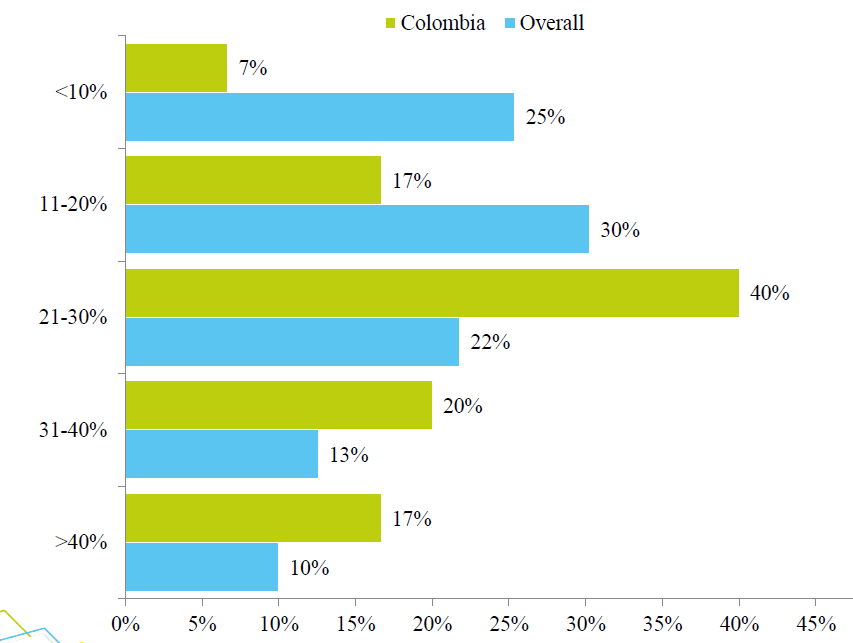


Do you see a role for probiotics when prescribing antibiotics to adult patients?


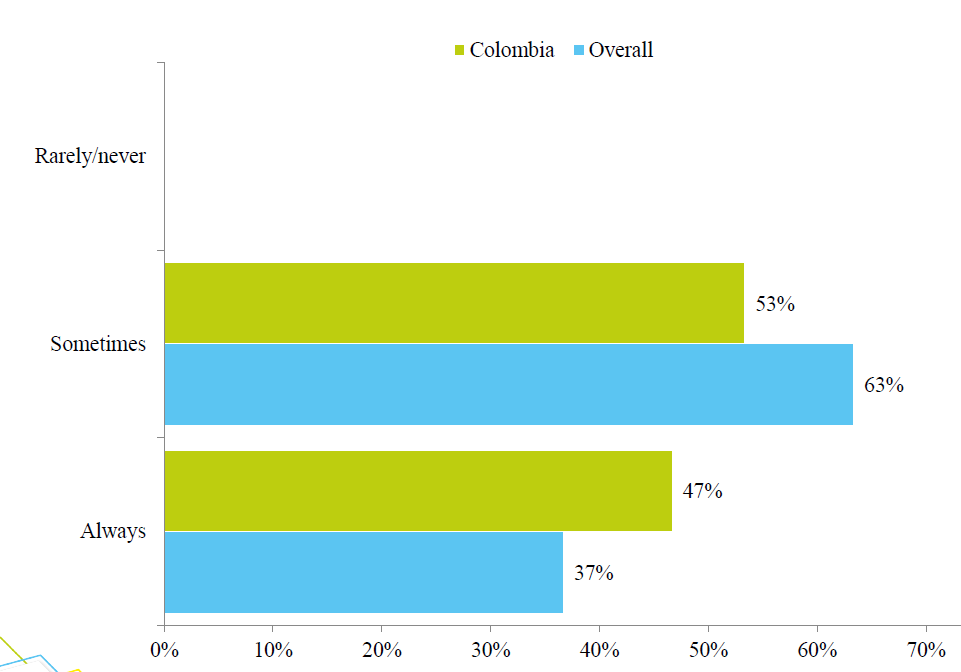


Thinking about antibiotics and the impact they can have on the microbiome, how would you respond to the following statements?


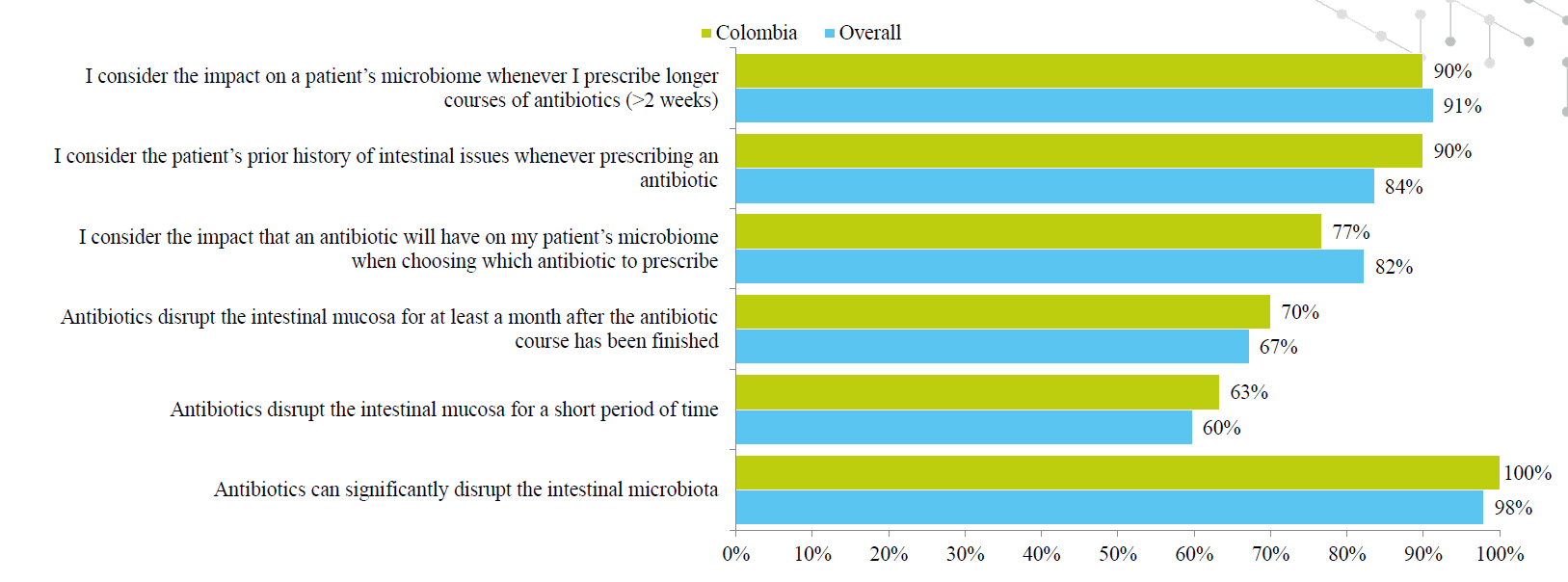


Thinking about antibiotics and the impact they can have on the microbiome, how do you respond to the following statements about probiotics?


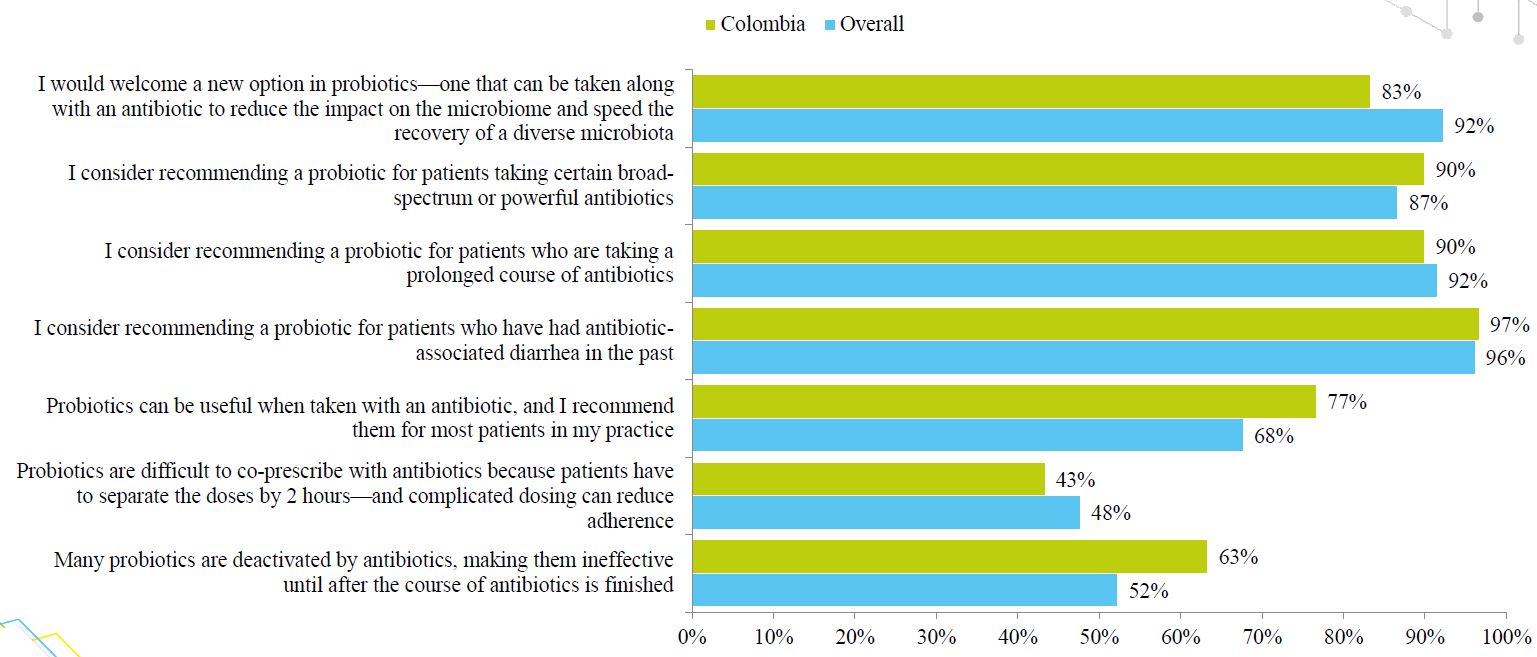


When prescribing antibiotics to adult patients today, for what percentage of patients do you also recommend probiotics?


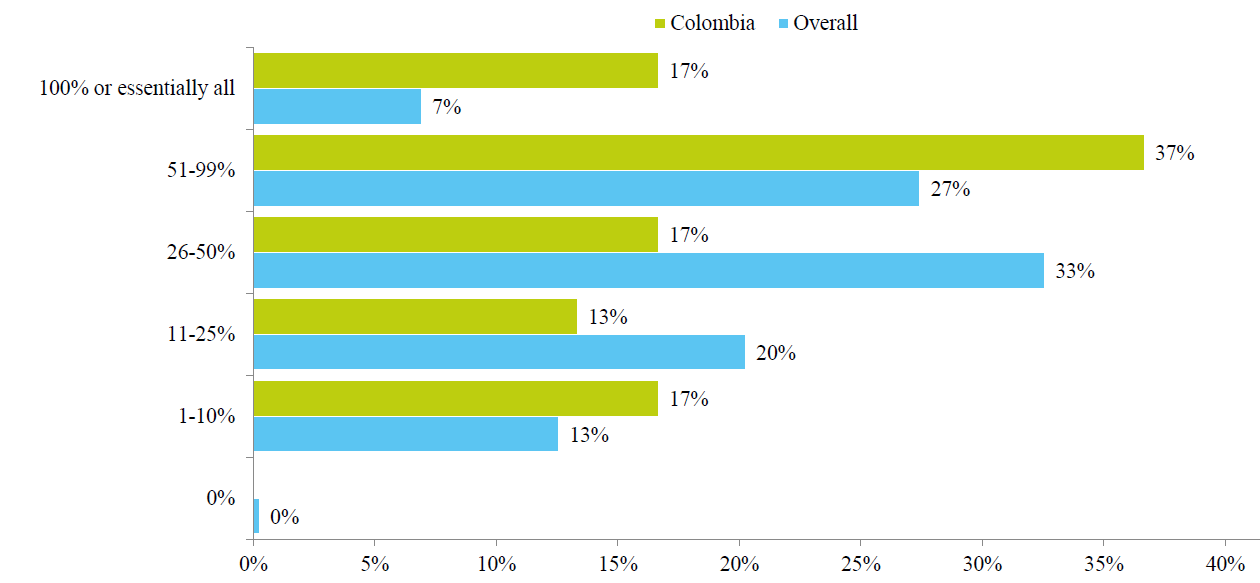


How would you respond to the following statements?


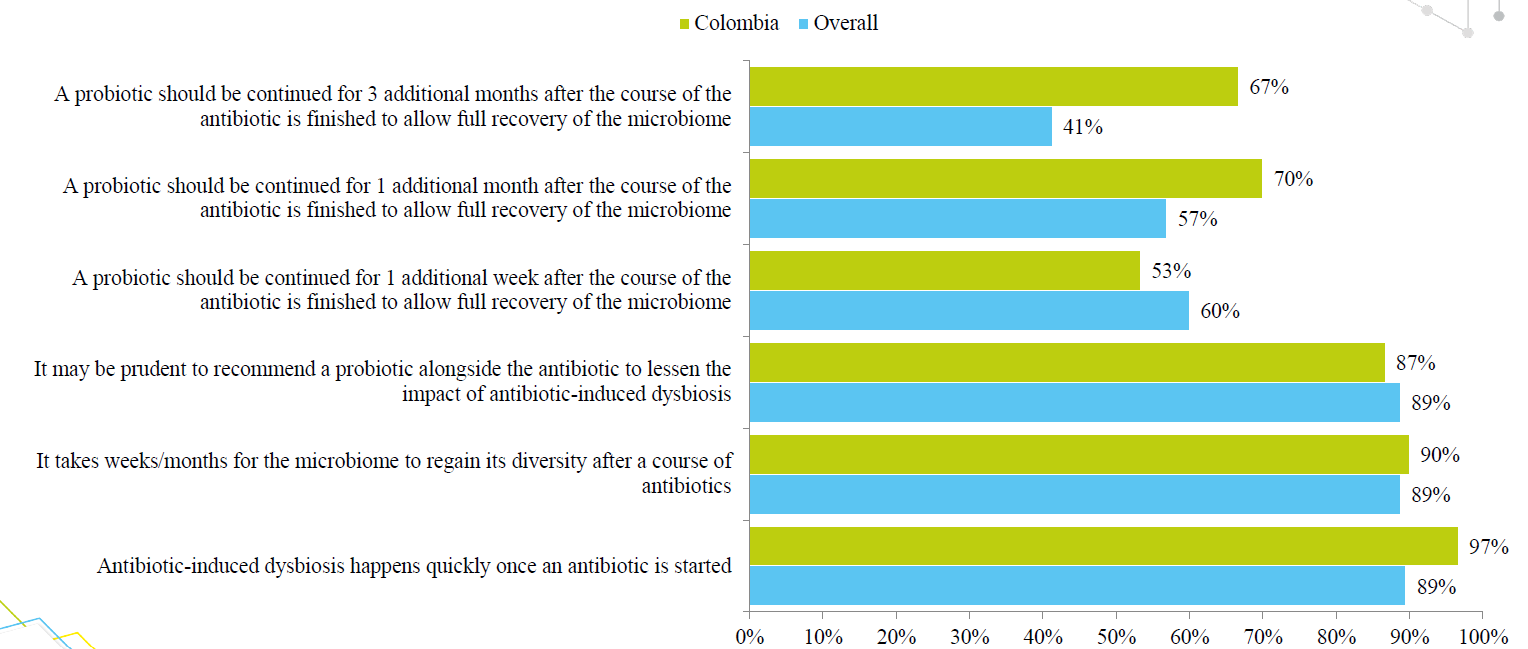


How would you respond to the following statements?


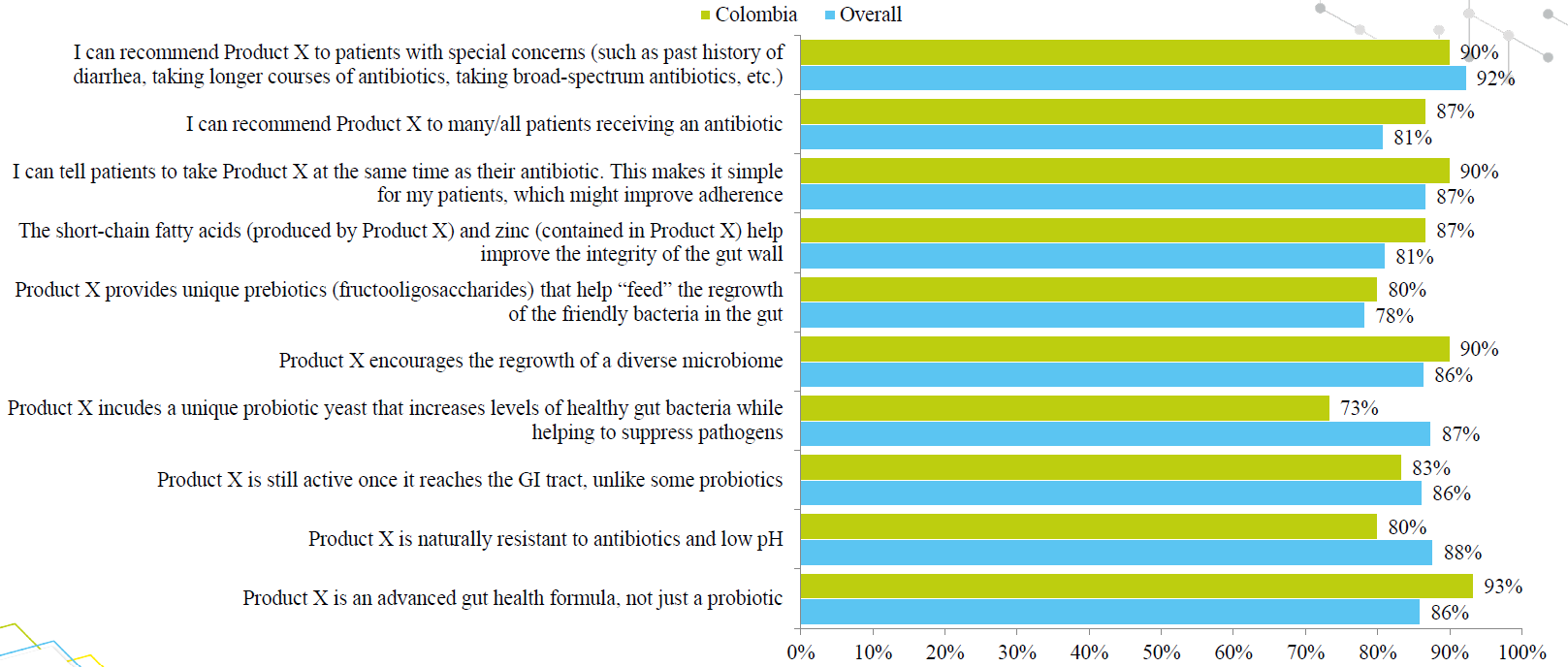


# Finland Report

In a typical week during cold and flu season, what percentage of the adult patients you see receive an antibiotic?


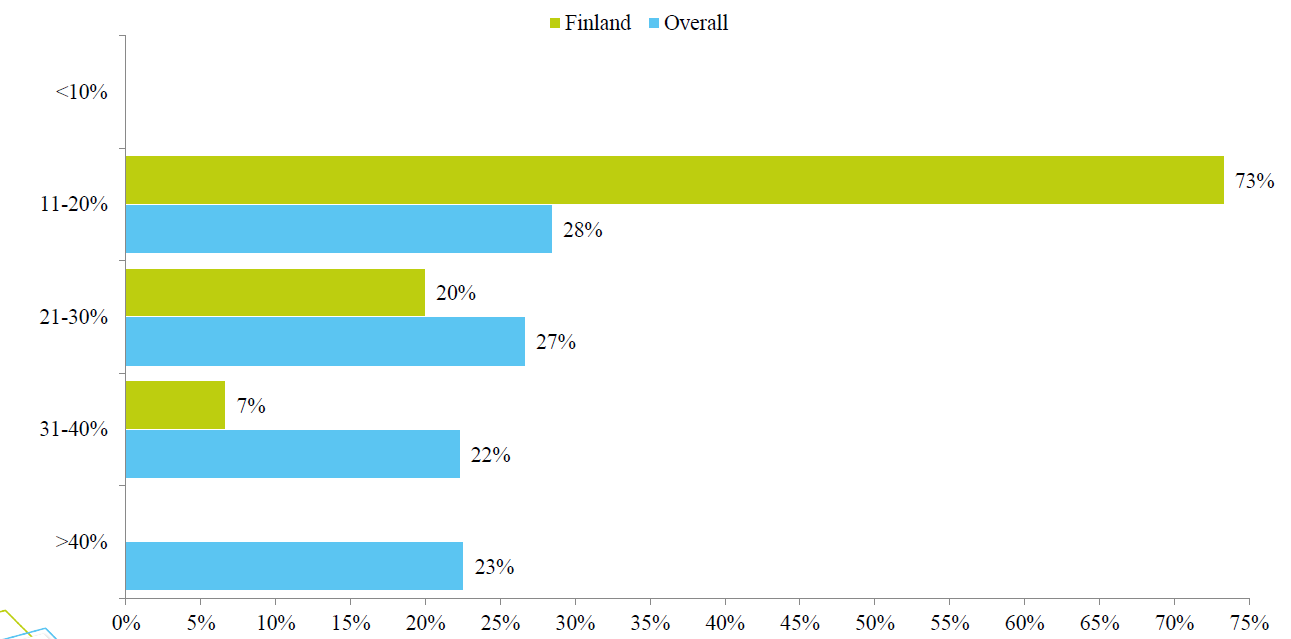


In a typical week, not during cold and flu season, what percentage of the adult patients you see receive an antibiotic?


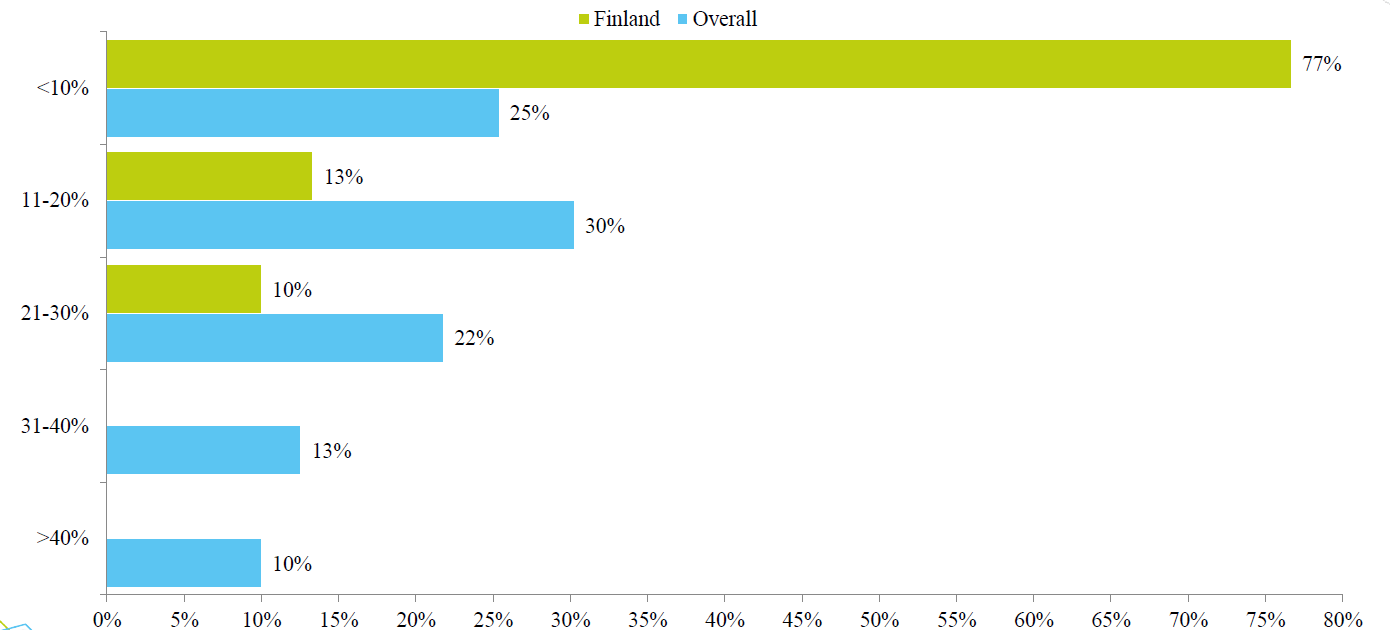


Do you see a role for probiotics when prescribing antibiotics to adult patients?


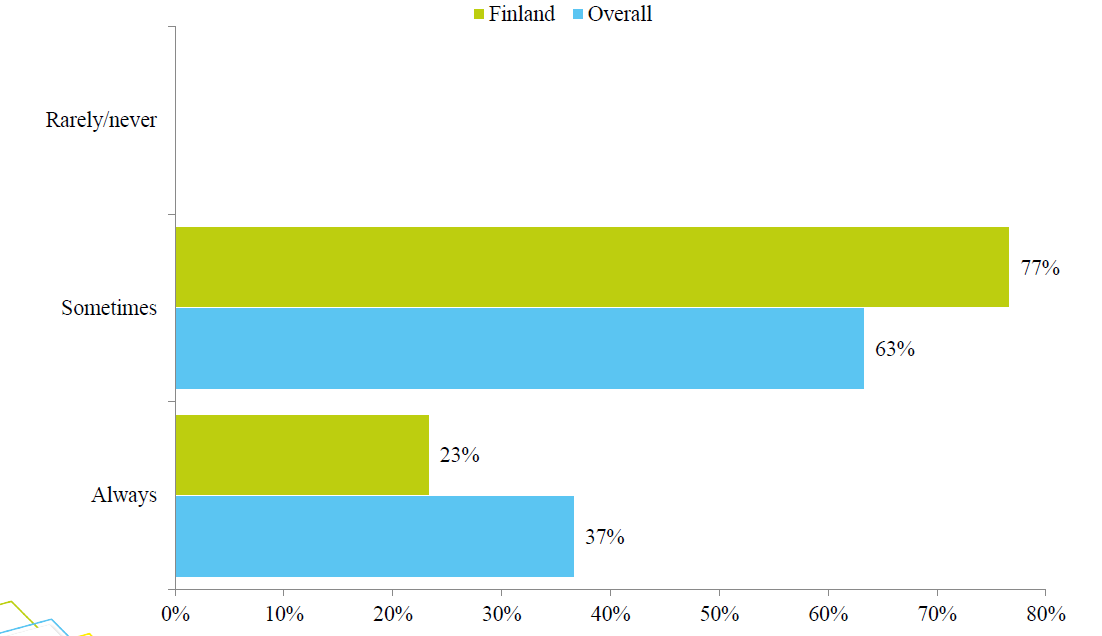


Thinking about antibiotics and the impact they can have on the microbiome, how would you respond to the following statements?


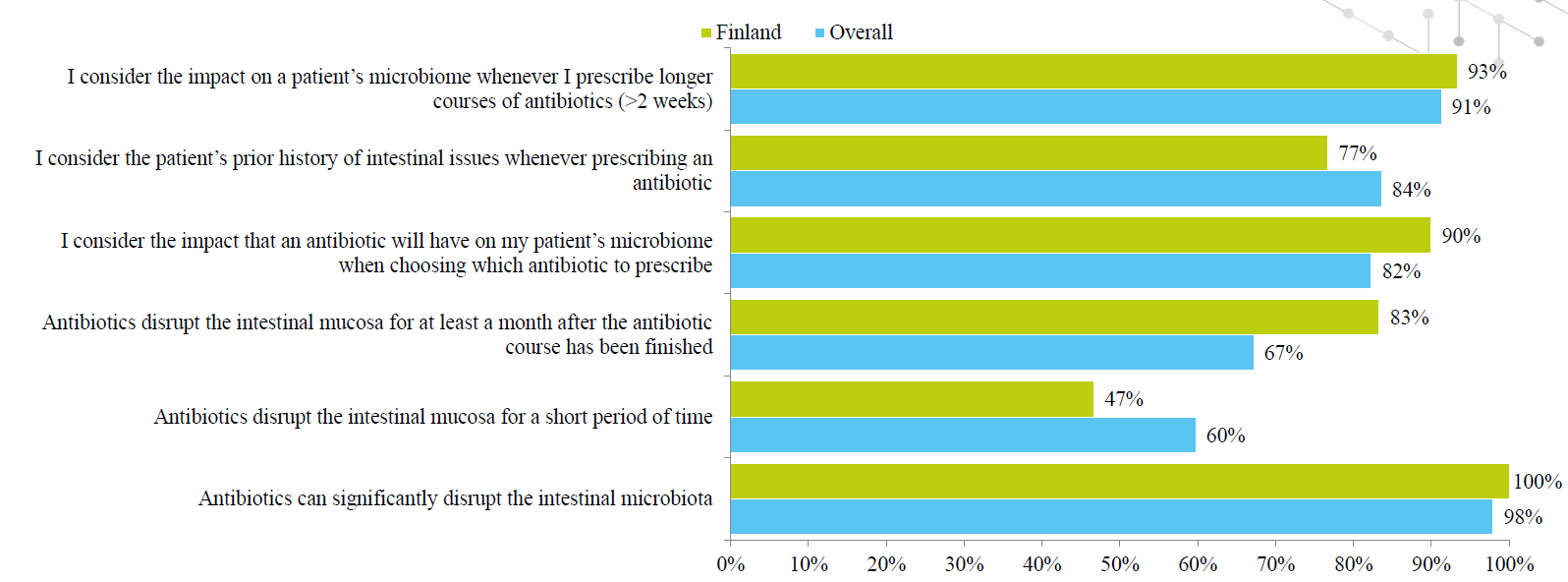


Thinking about antibiotics and the impact they can have on the microbiome, how do you respond to the following statements about probiotics?


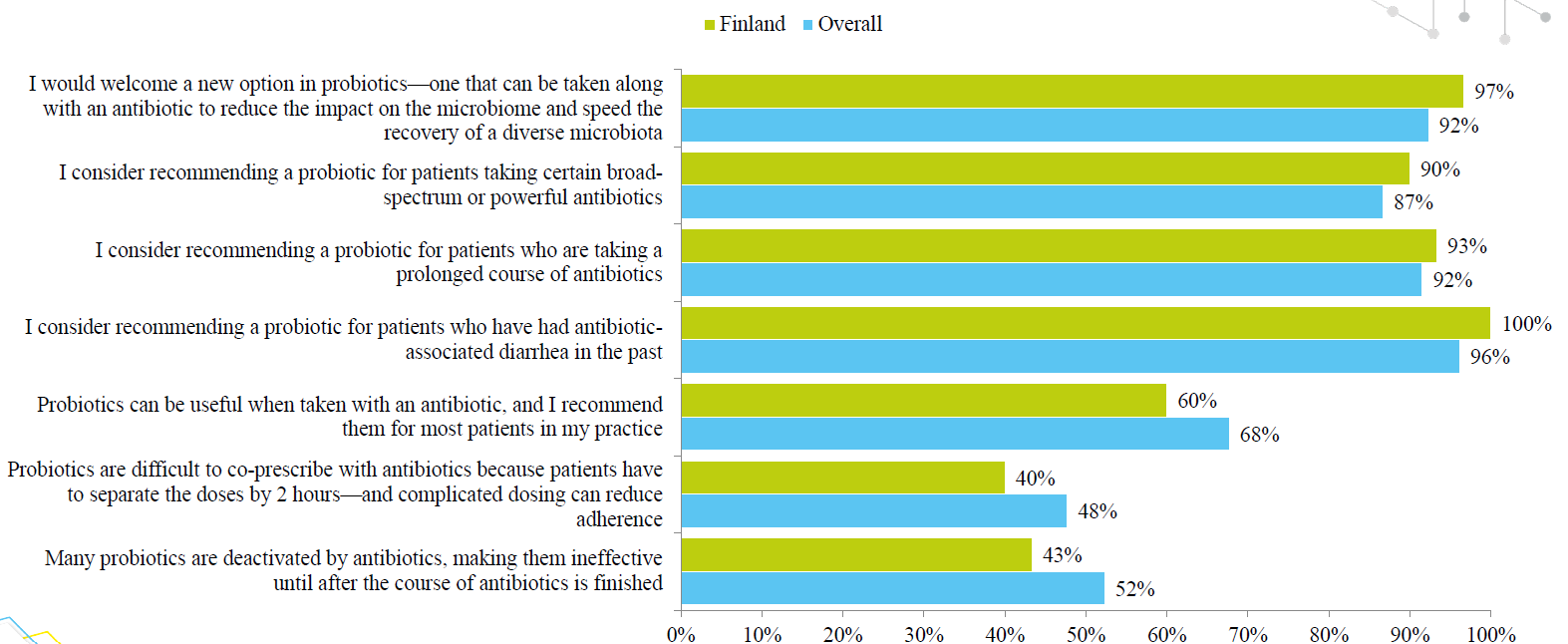


When prescribing antibiotics to adult patients today, for what percentage of patients do you also recommend probiotics?


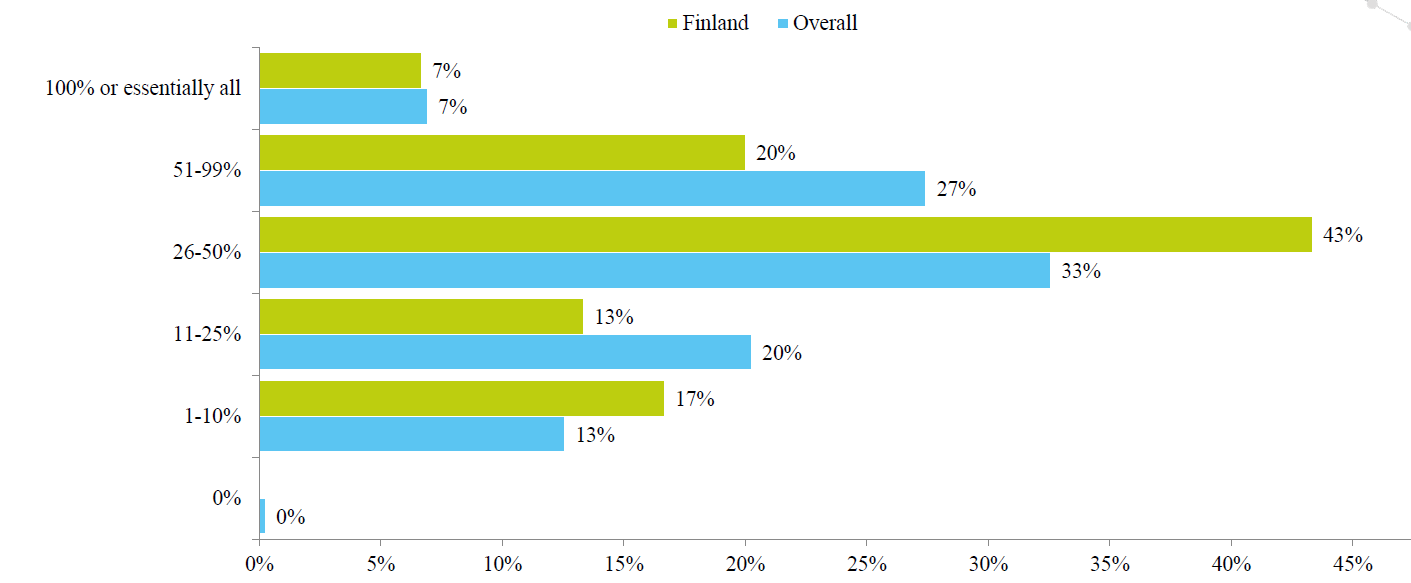


How would you respond to the following statements?


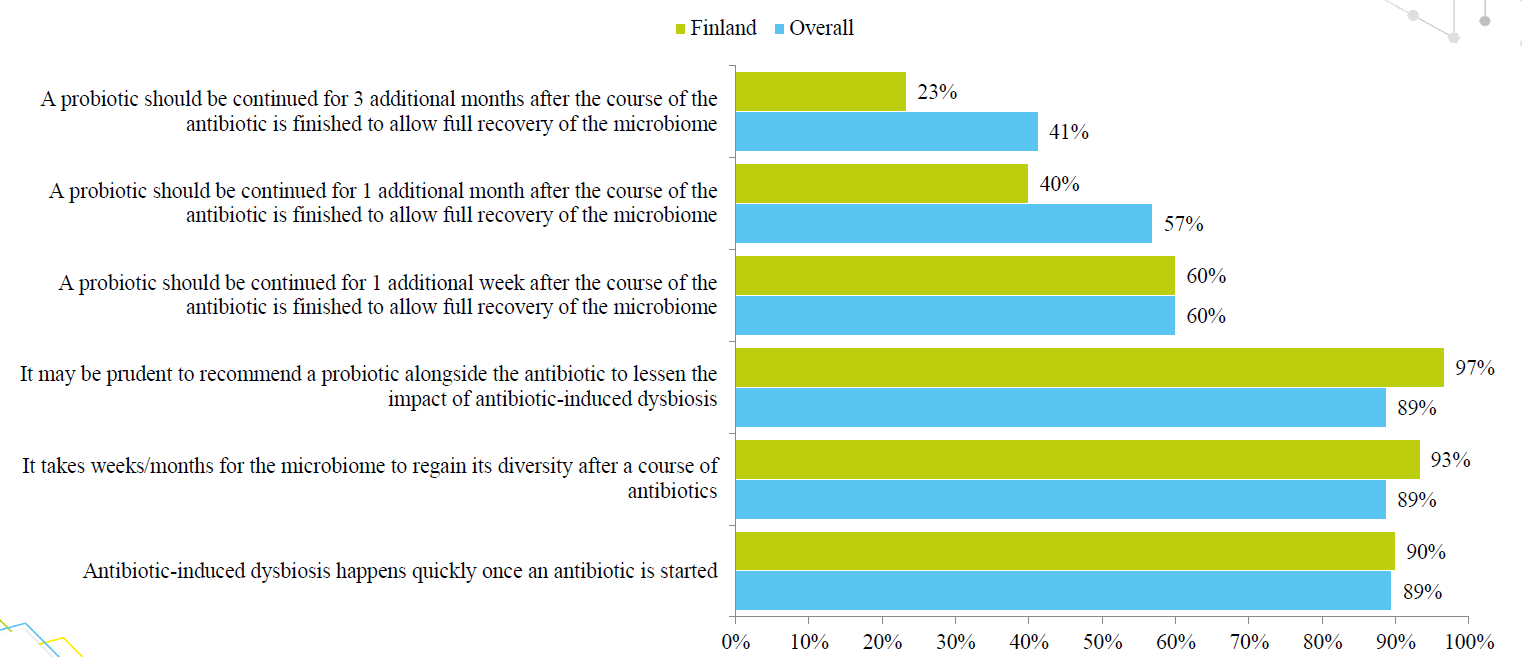


How would you respond to the following statements?


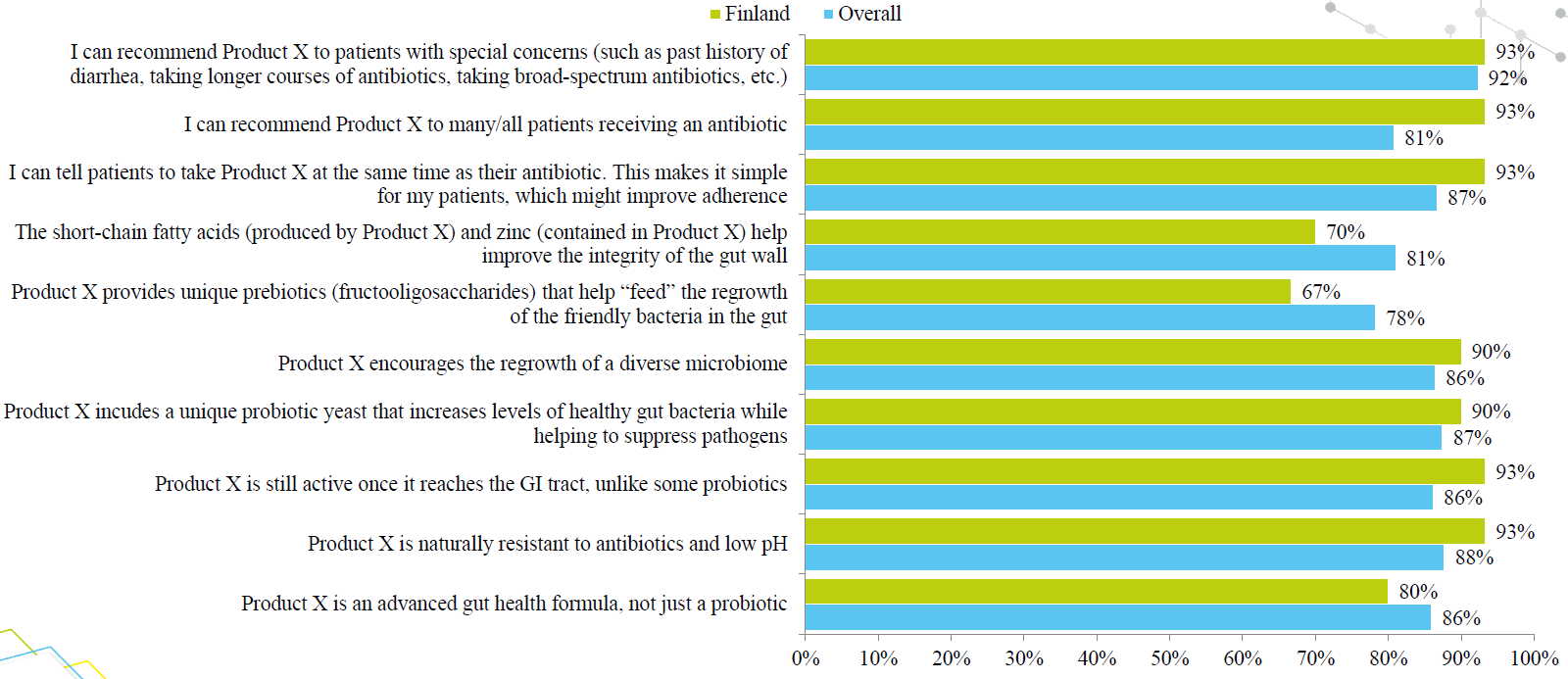


# France Report

In a typical week during cold and flu season, what percentage of the adult patients you see receive an antibiotic?


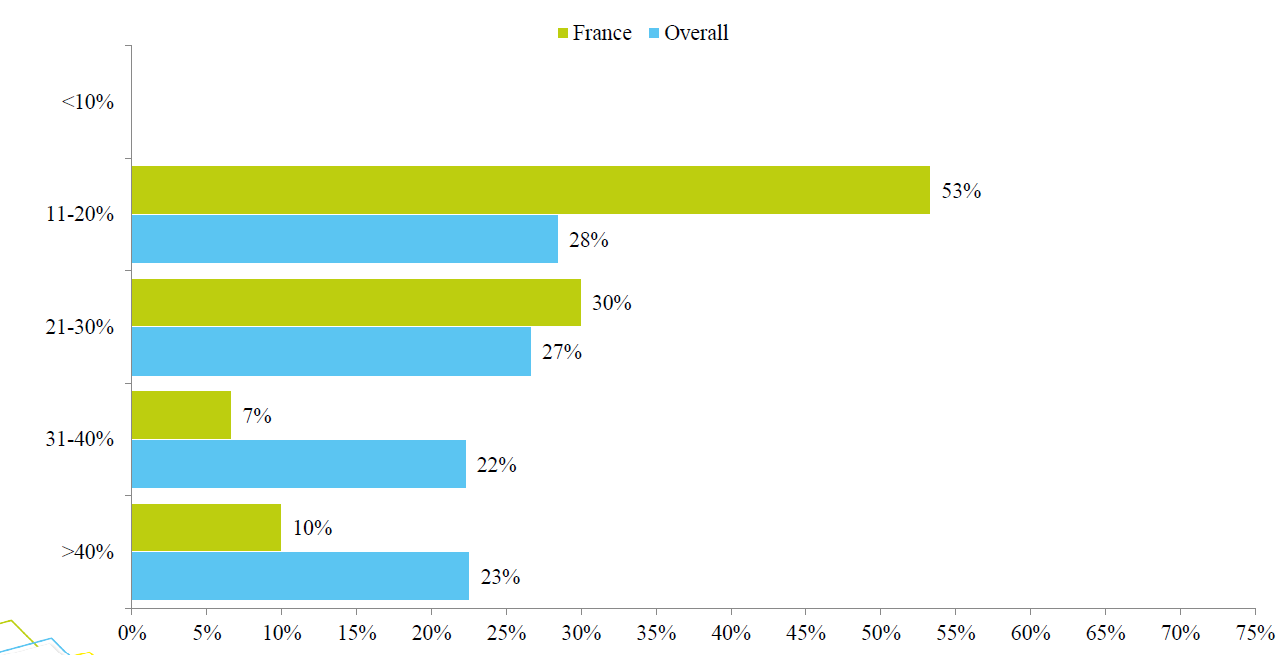


In a typical week, not during cold and flu season, what percentage of the adult patients you see receive an antibiotic?


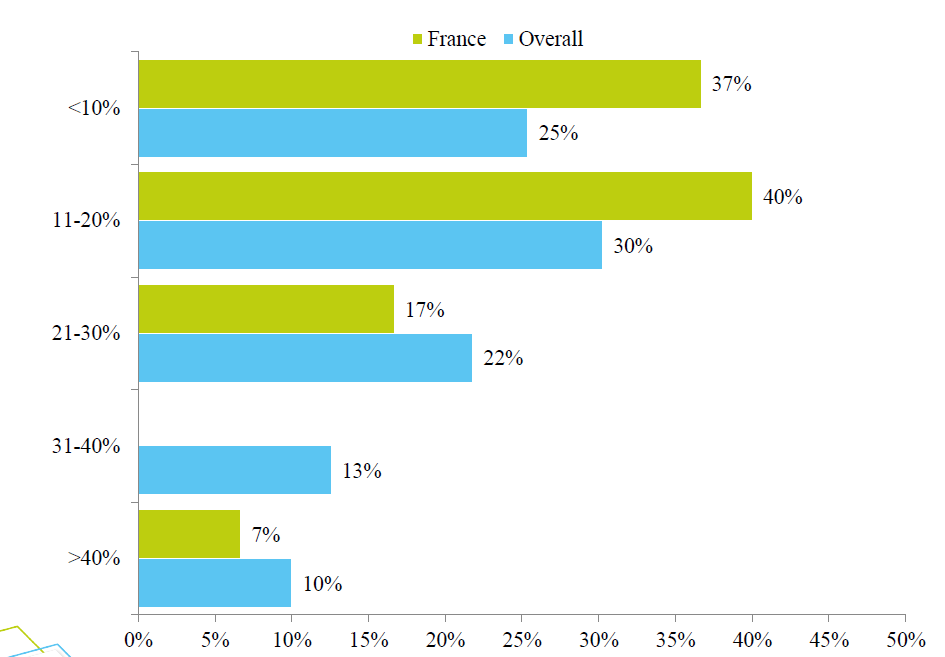


Do you see a role for probiotics when prescribing antibiotics to adult patients?


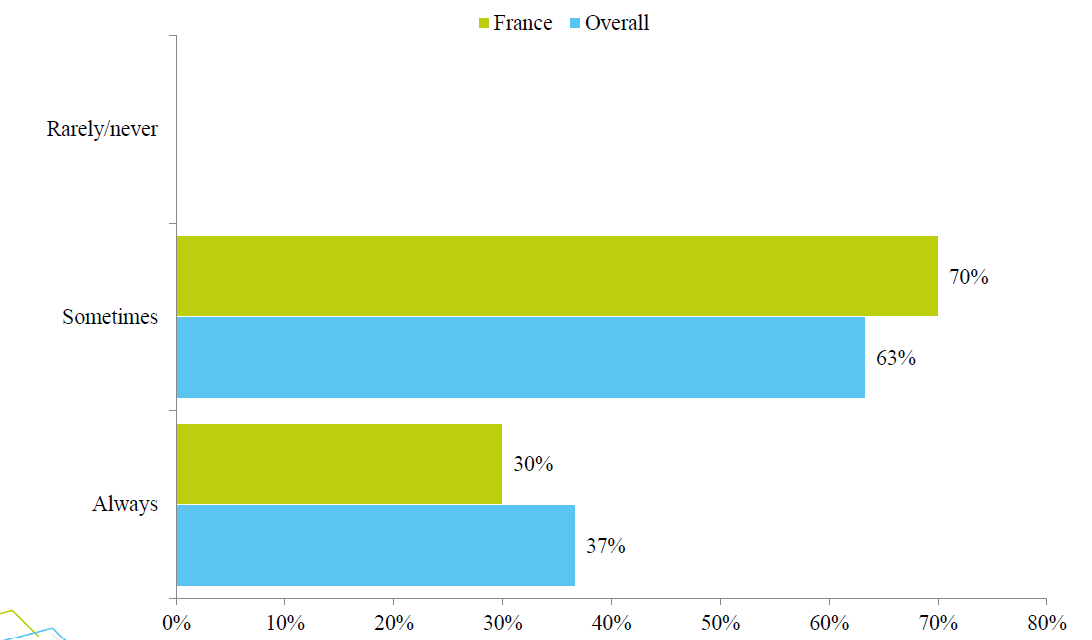


Thinking about antibiotics and the impact they can have on the microbiome, how would you respond to the following statements?


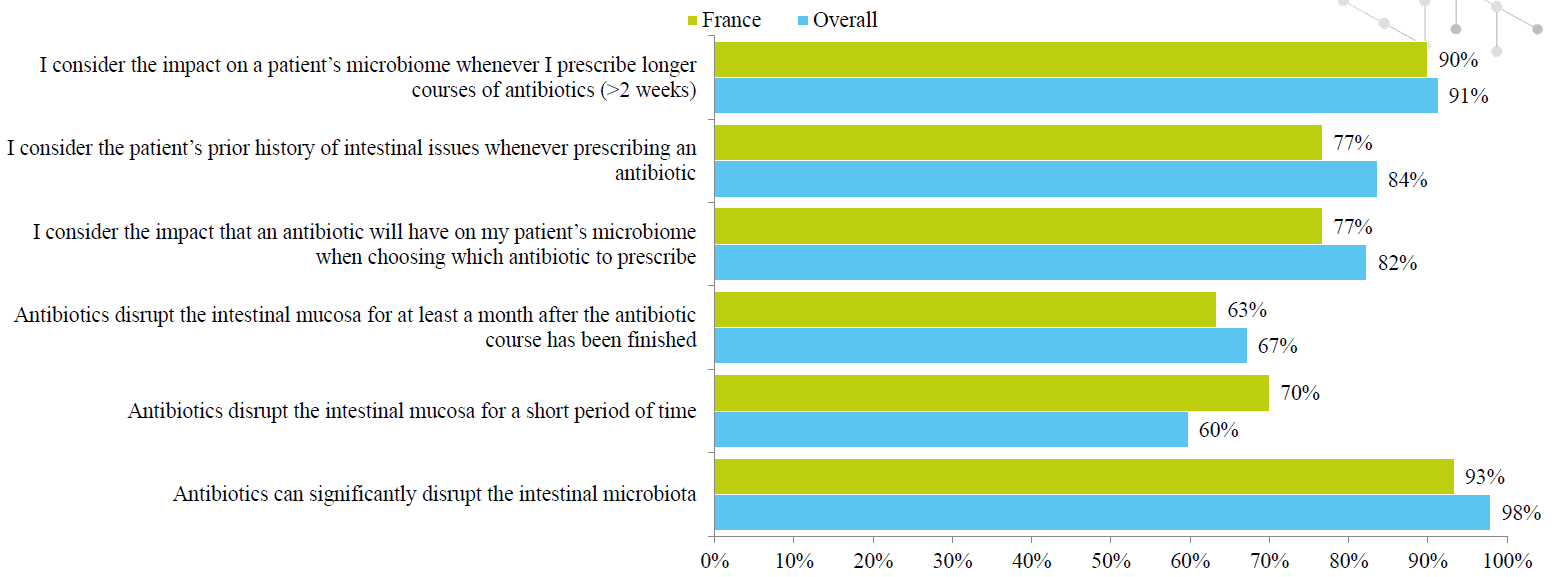


Thinking about antibiotics and the impact they can have on the microbiome, how do you respond to the following statements about probiotics?


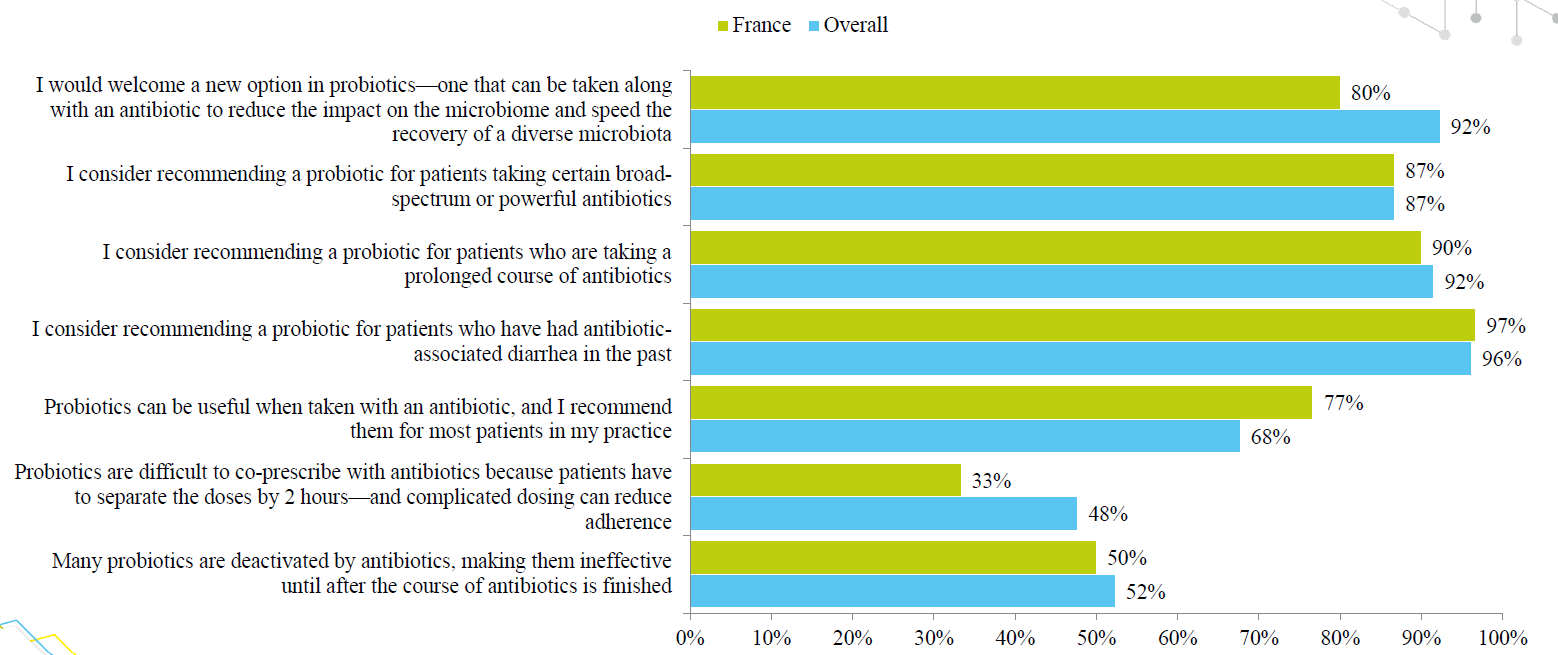


When prescribing antibiotics to adult patients today, for what percentage of patients do you also recommend probiotics?


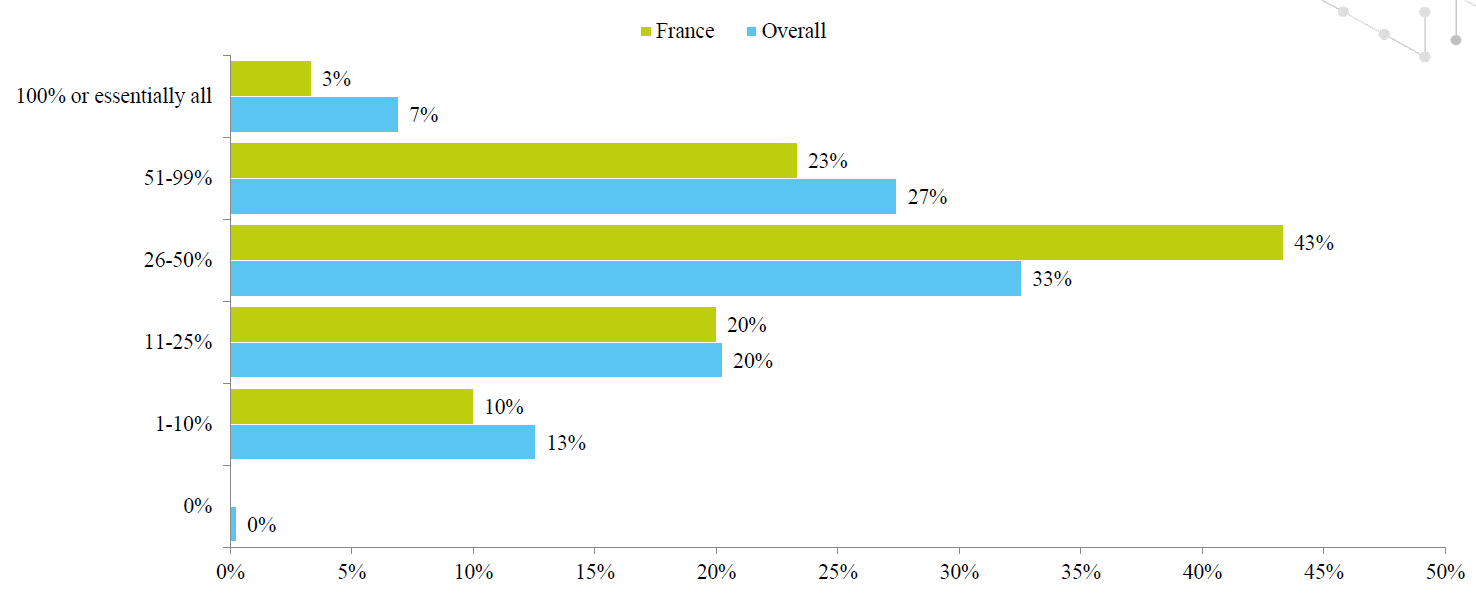


How would you respond to the following statements?


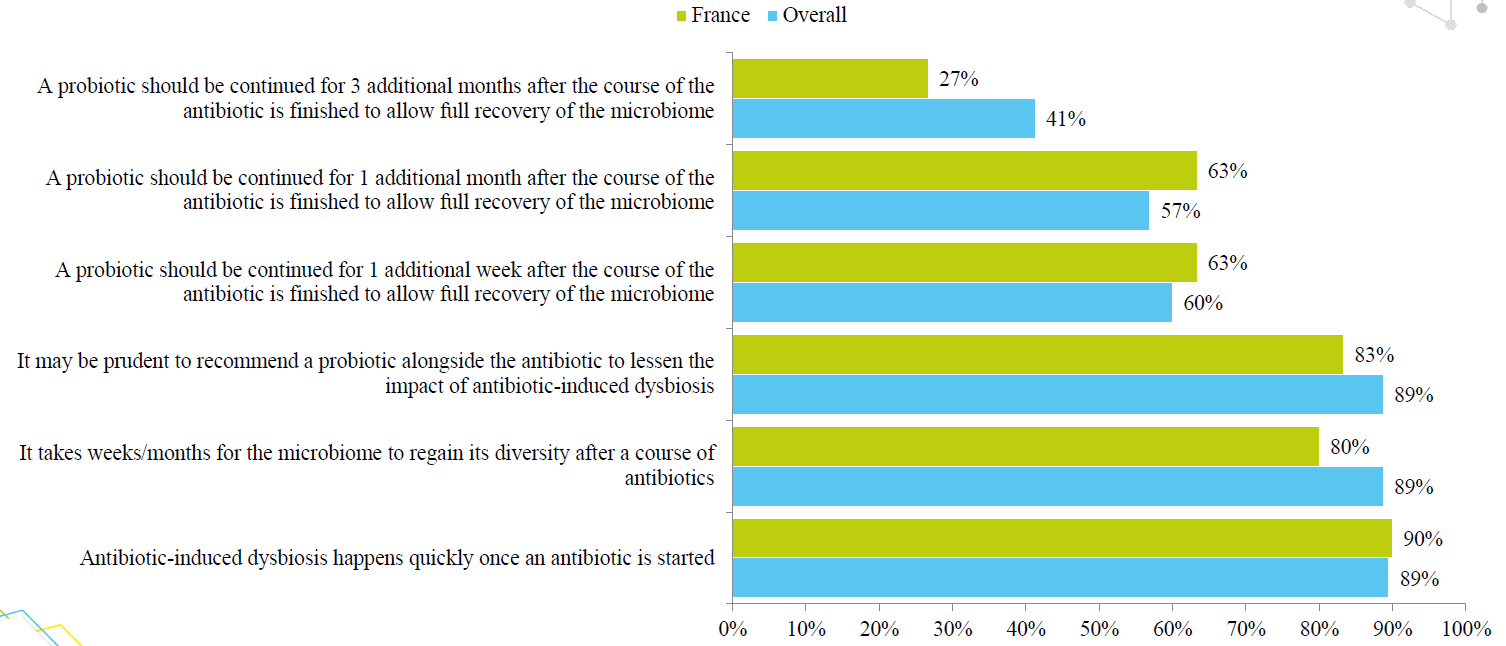


How would you respond to the following statements?


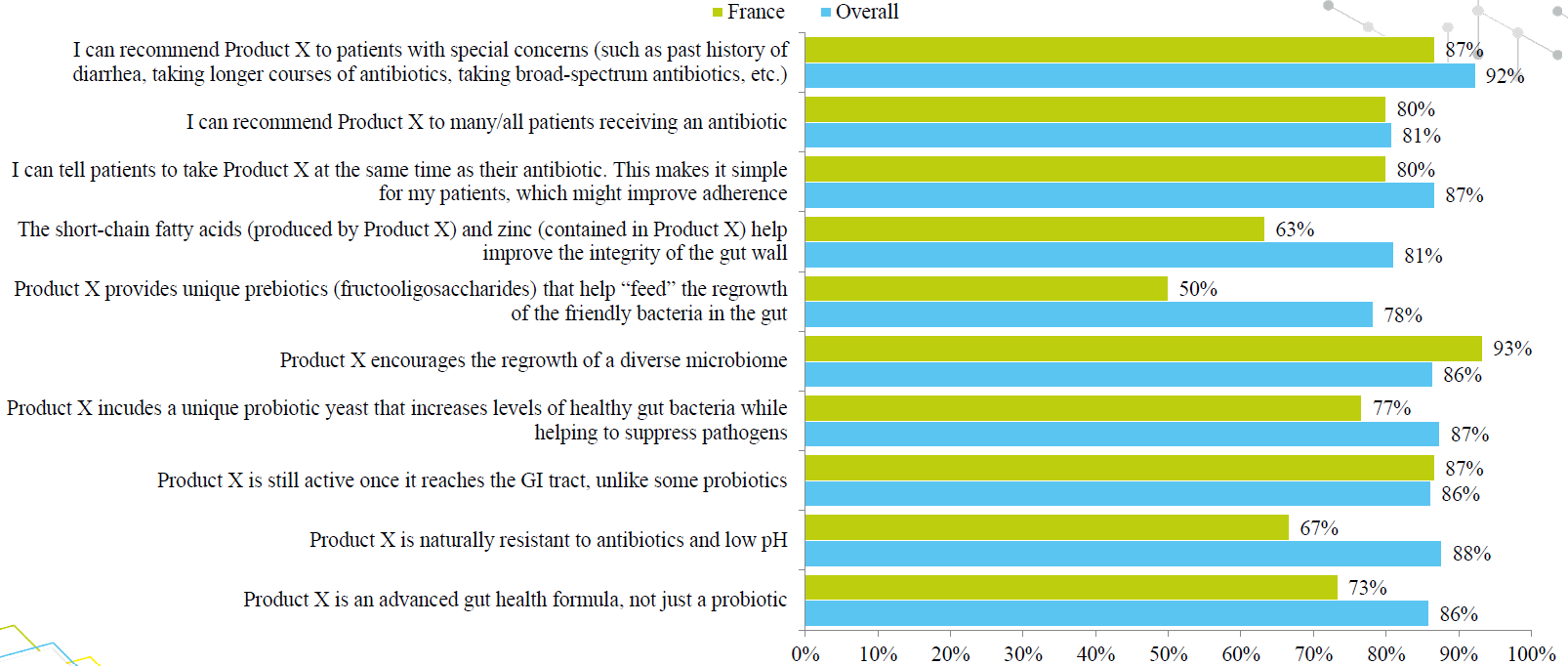


# Germany Report

In a typical week during cold and flu season, what percentage of the adult patients you see receive an antibiotic?


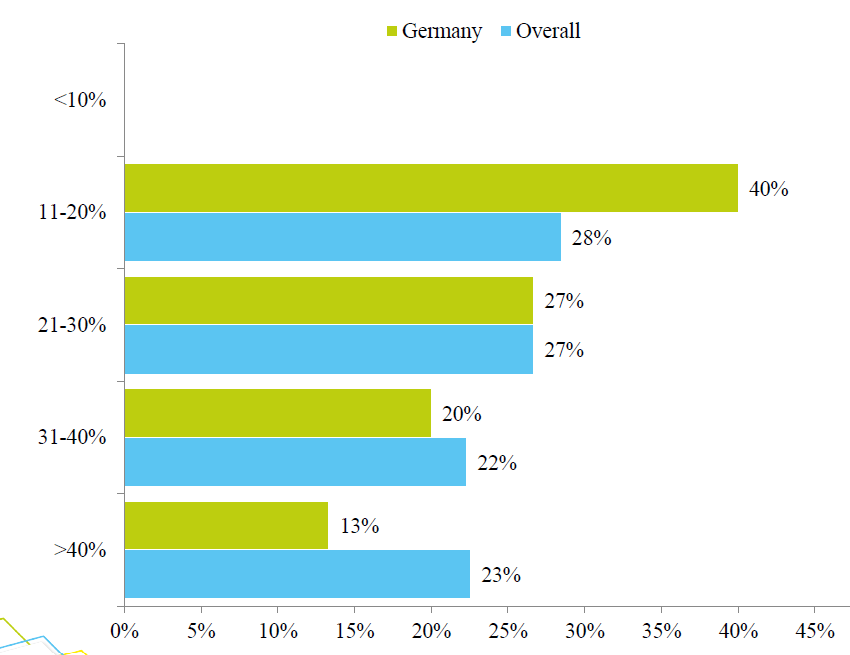


In a typical week, not during cold and flu season, what percentage of the adult patients you see receive an antibiotic?


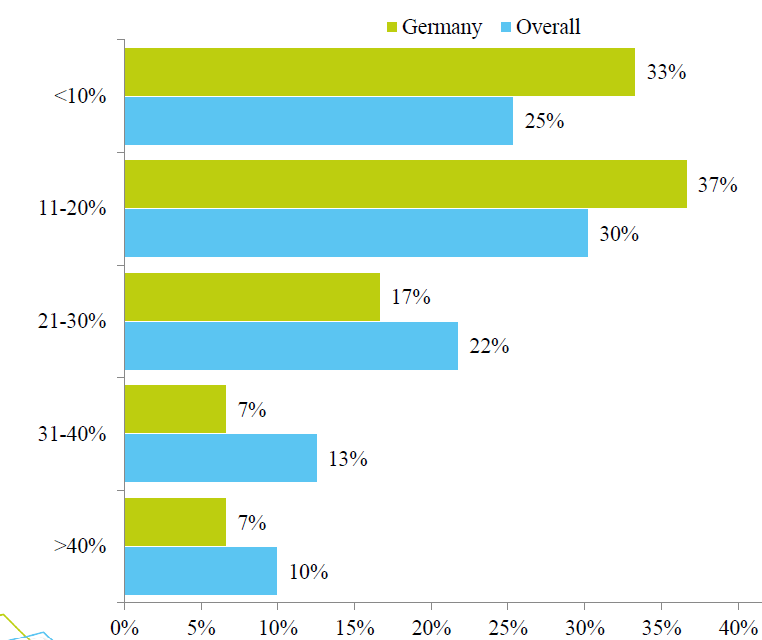


Do you see a role for probiotics when prescribing antibiotics to adult patients?


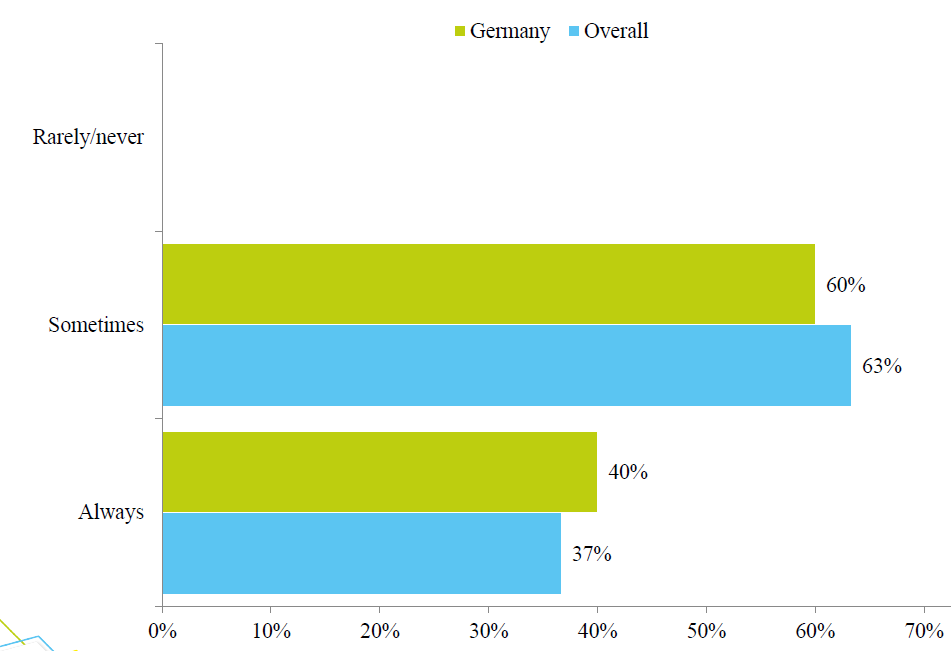


Thinking about antibiotics and the impact they can have on the microbiome, how would you respond to the following statements?


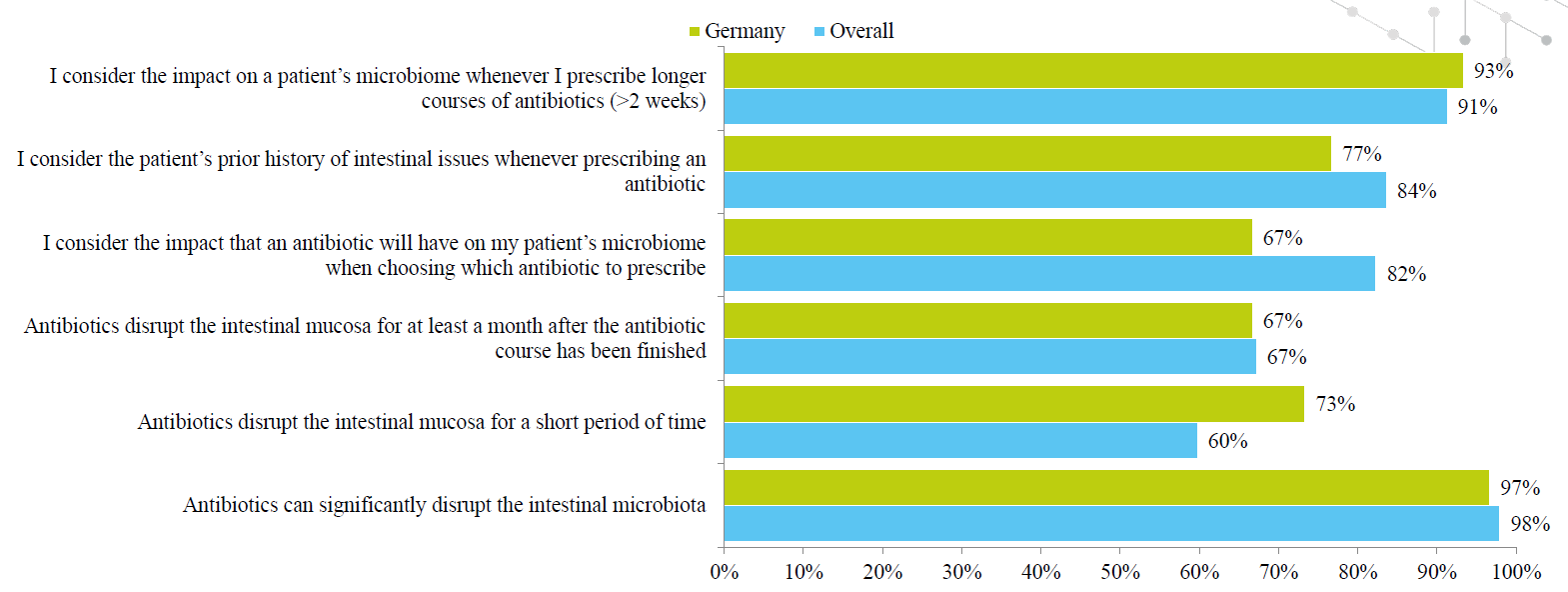


Thinking about antibiotics and the impact they can have on the microbiome, how do you respond to the following statements about probiotics?


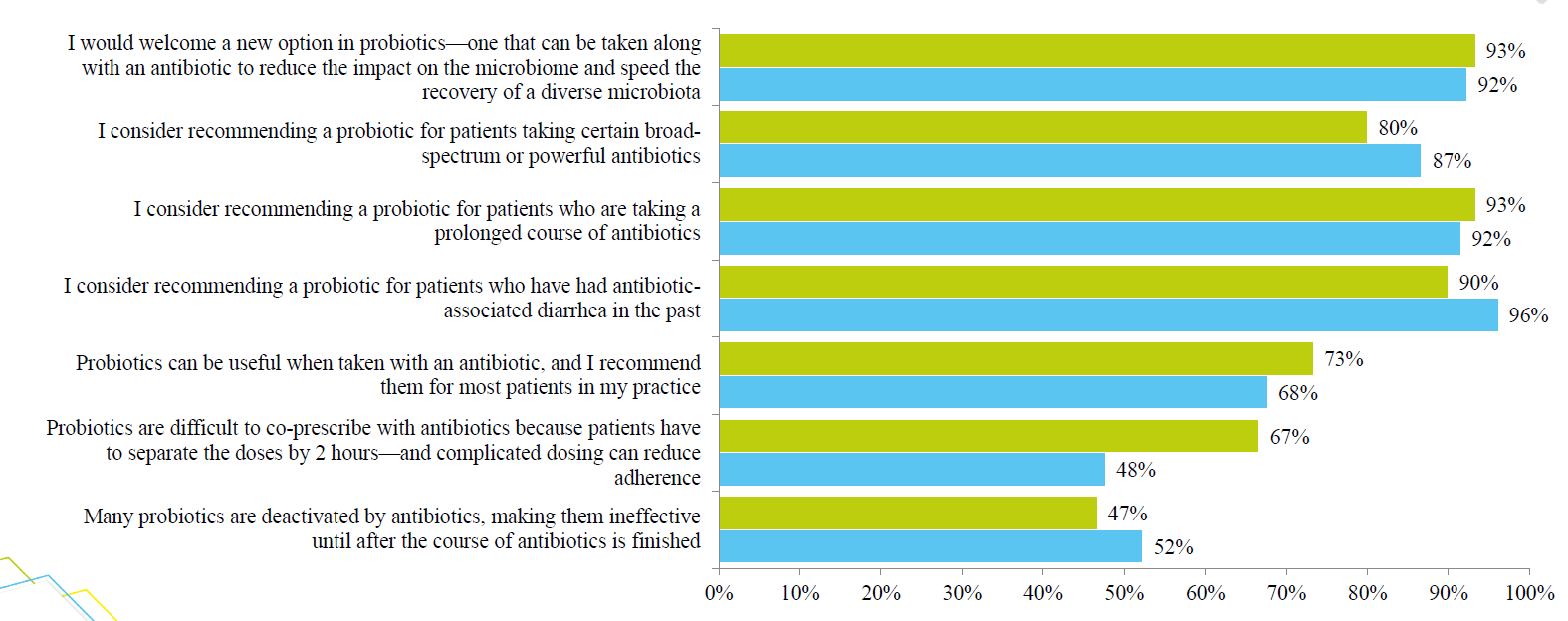


When prescribing antibiotics to adult patients today, for what percentage of patients do you also recommend probiotics?


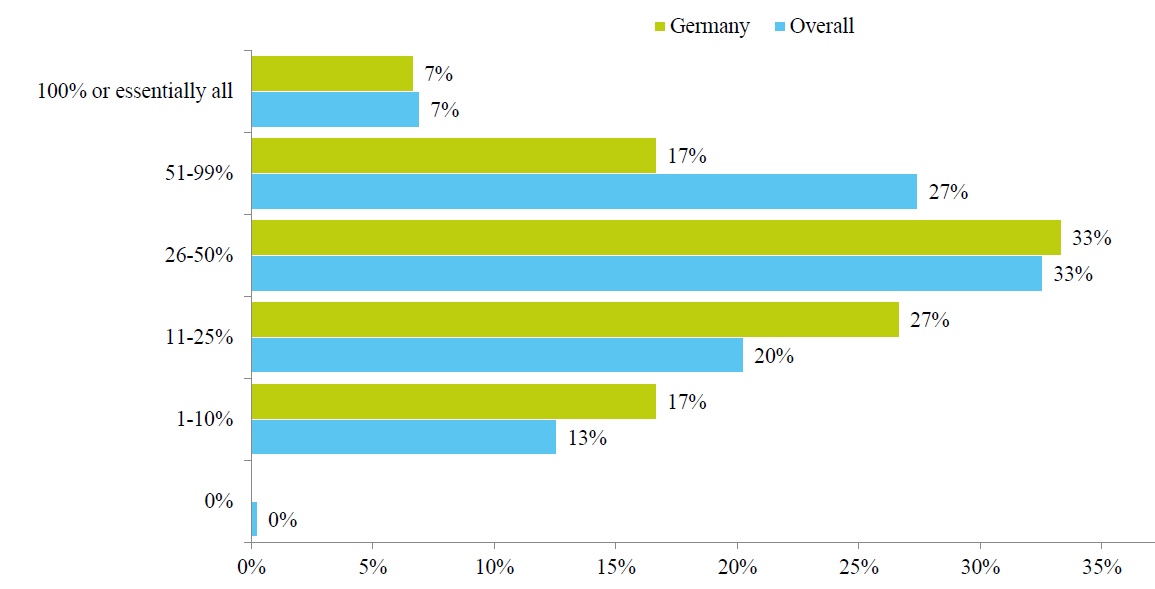


How would you respond to the following statements?


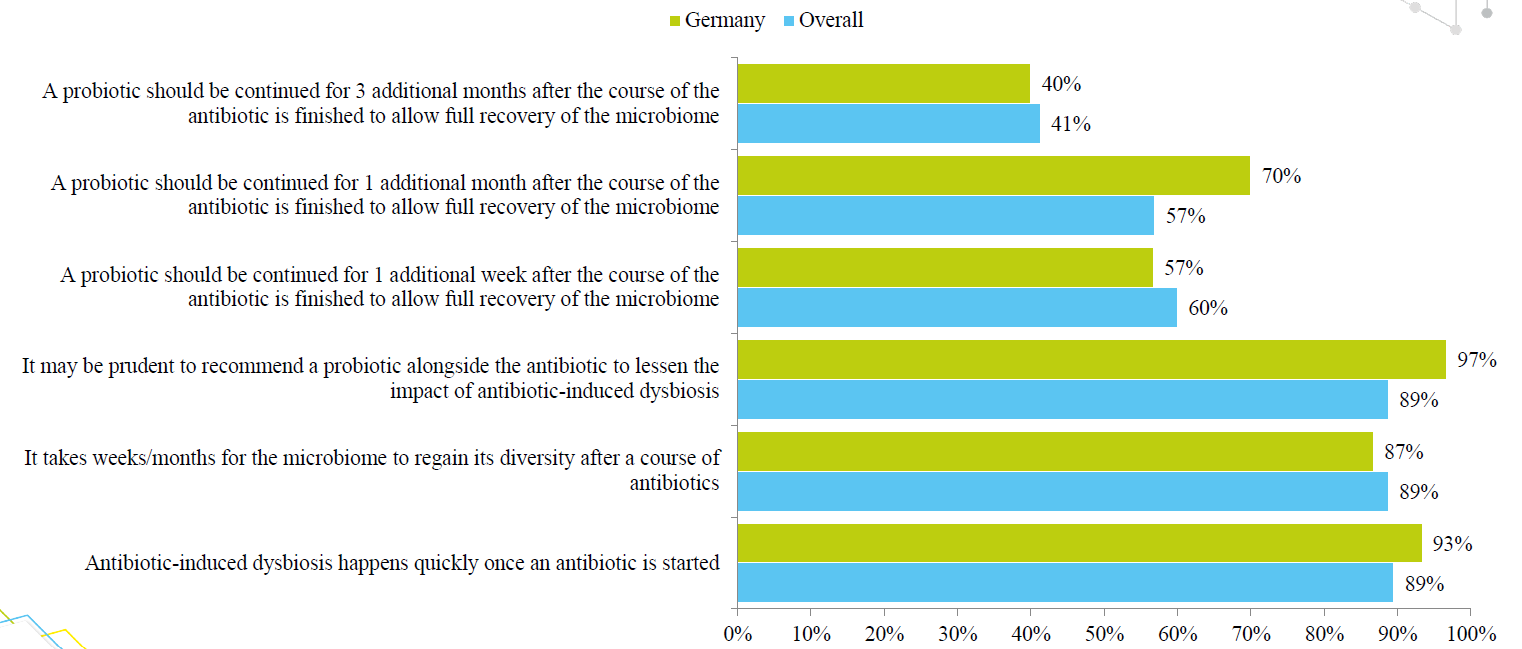


How would you respond to the following statements?


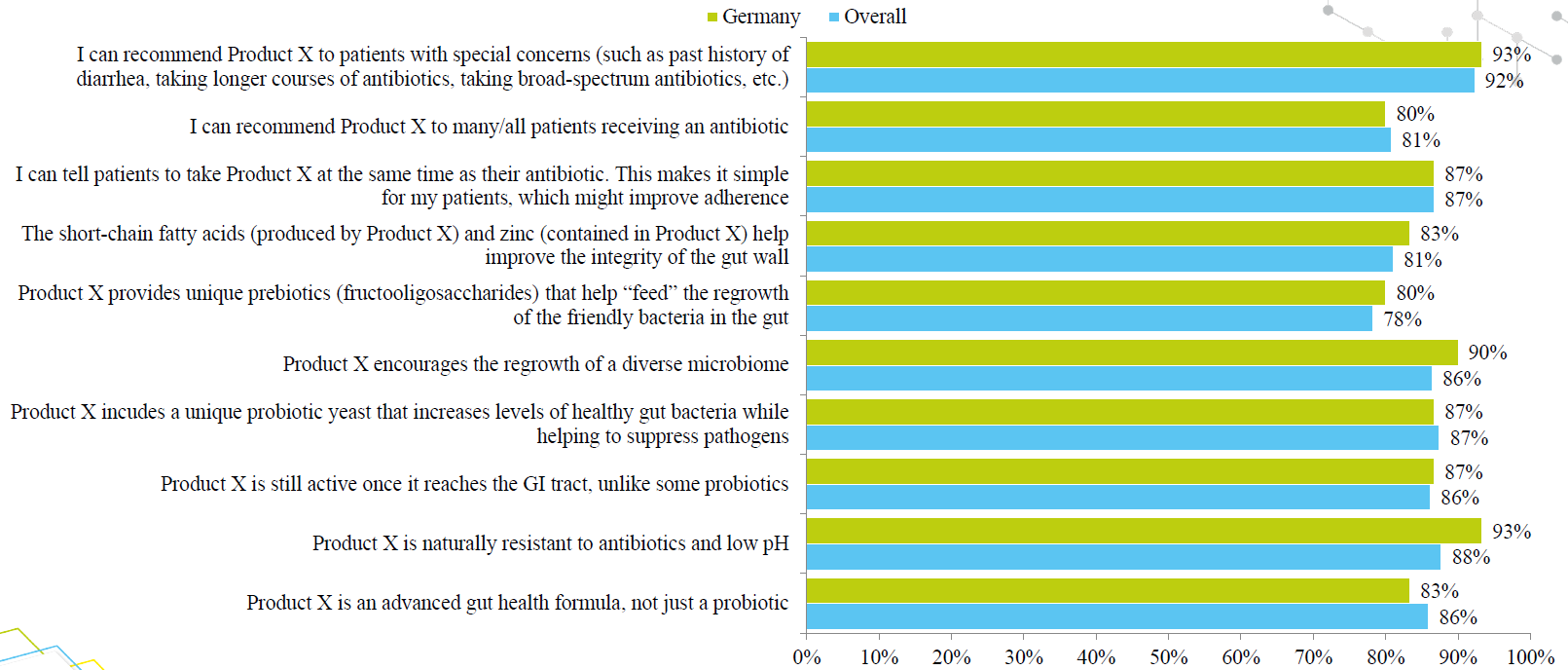


# Ireland Report

In a typical week during cold and flu season, what percentage of the adult patients you see receive an antibiotic?


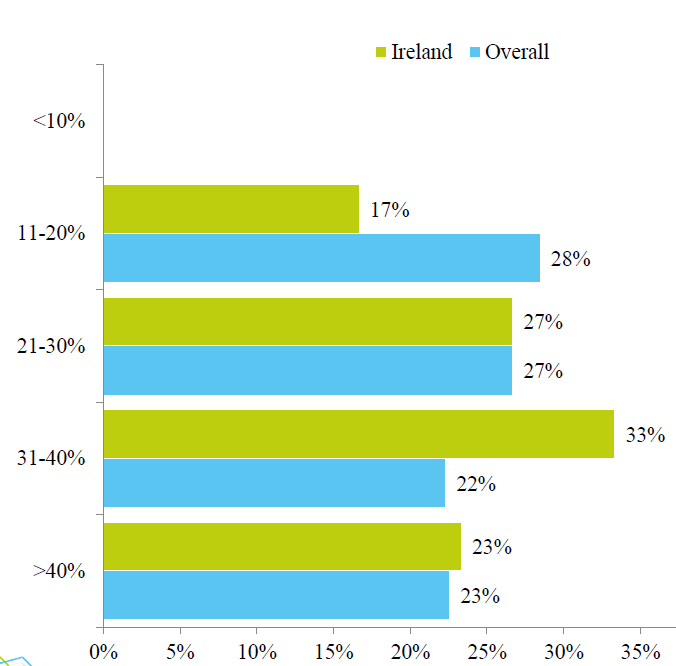


In a typical week, not during cold and flu season, what percentage of the adult patients you see receive an antibiotic?


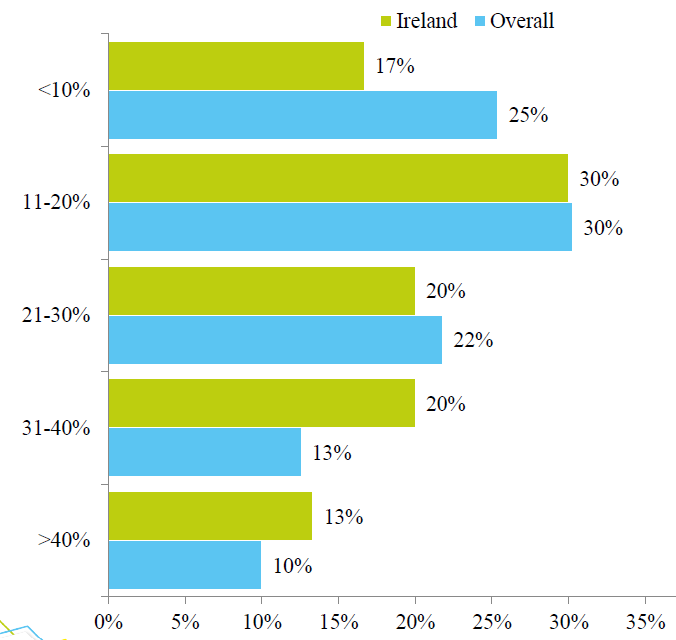


Do you see a role for probiotics when prescribing antibiotics to adult patients?


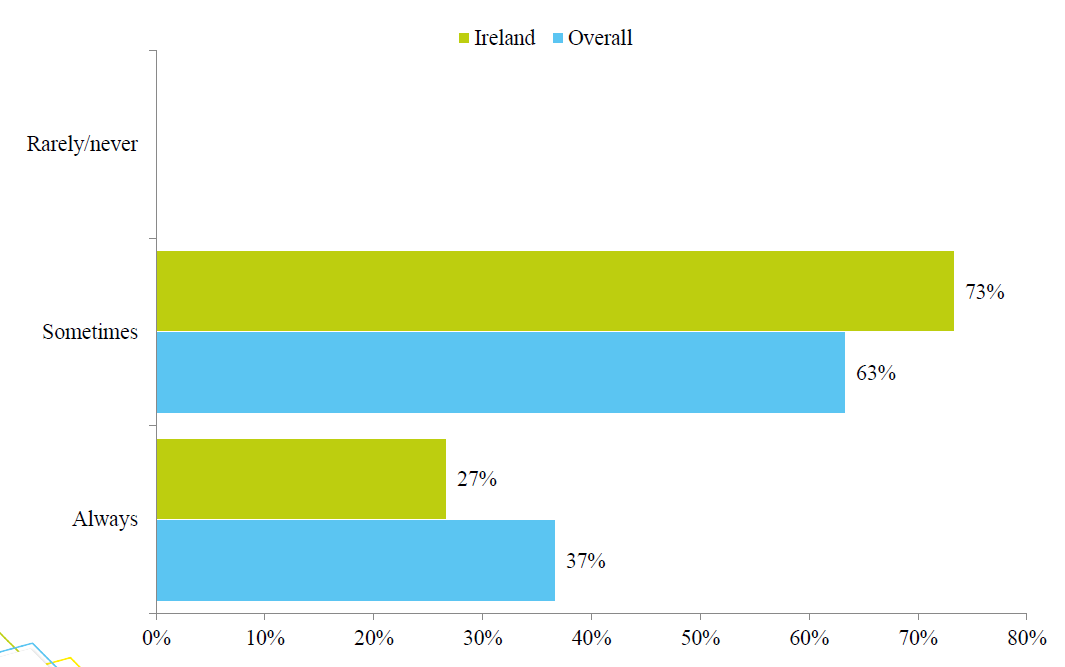


Thinking about antibiotics and the impact they can have on the microbiome, how would you respond to the following statements?


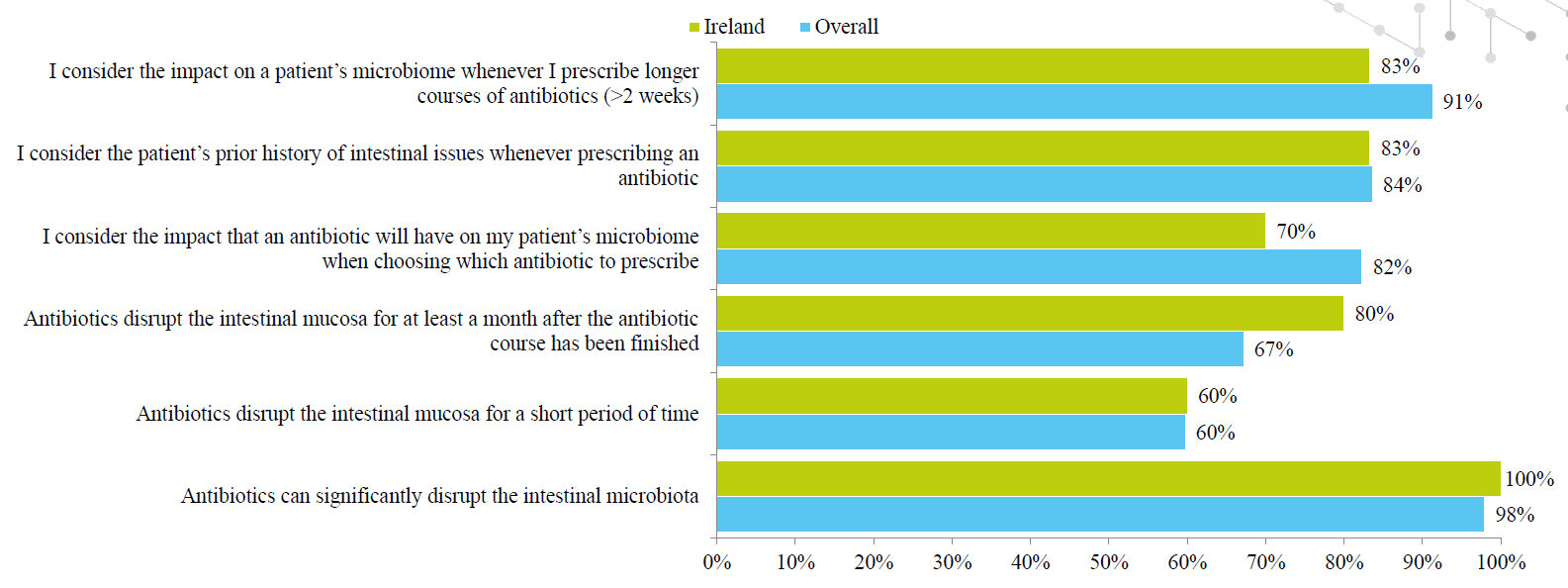


Thinking about antibiotics and the impact they can have on the microbiome, how do you respond to the following statements about probiotics?


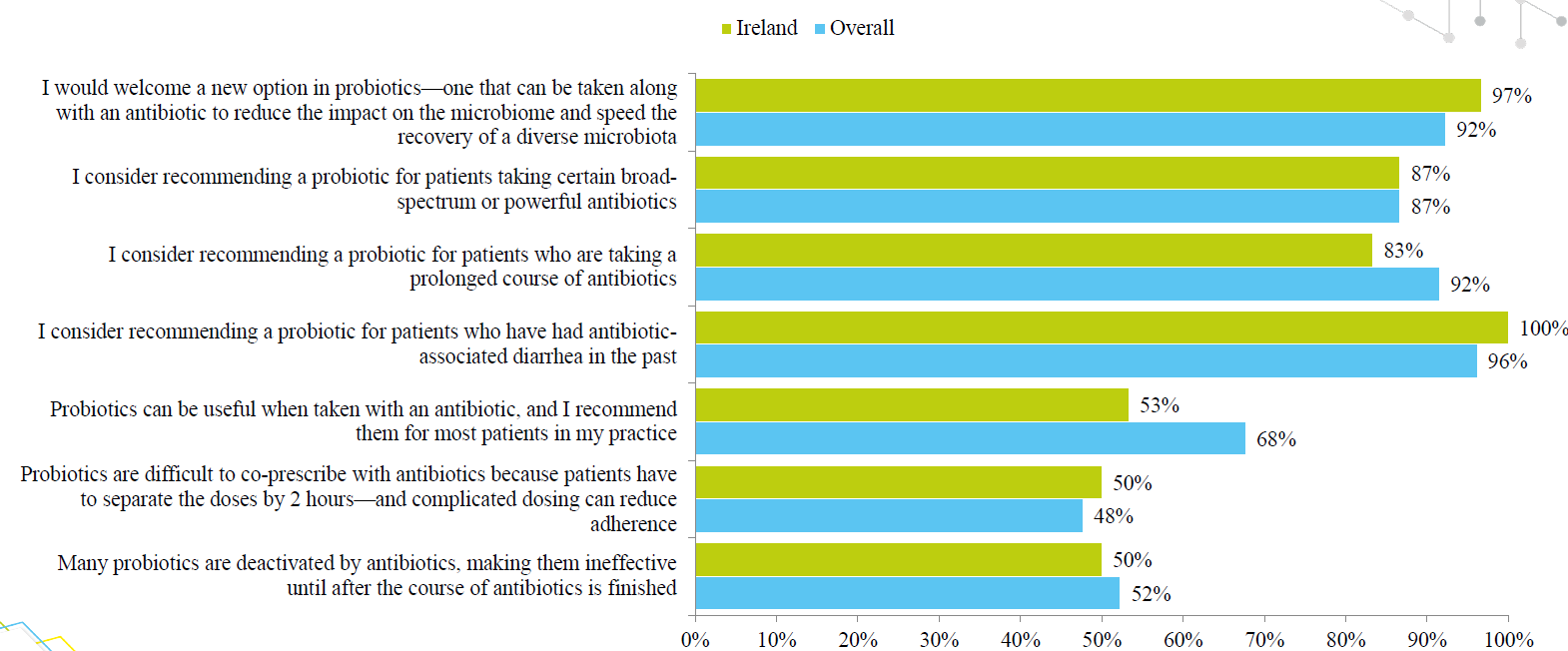


When prescribing antibiotics to adult patients today, for what percentage of patients do you also recommend probiotics?


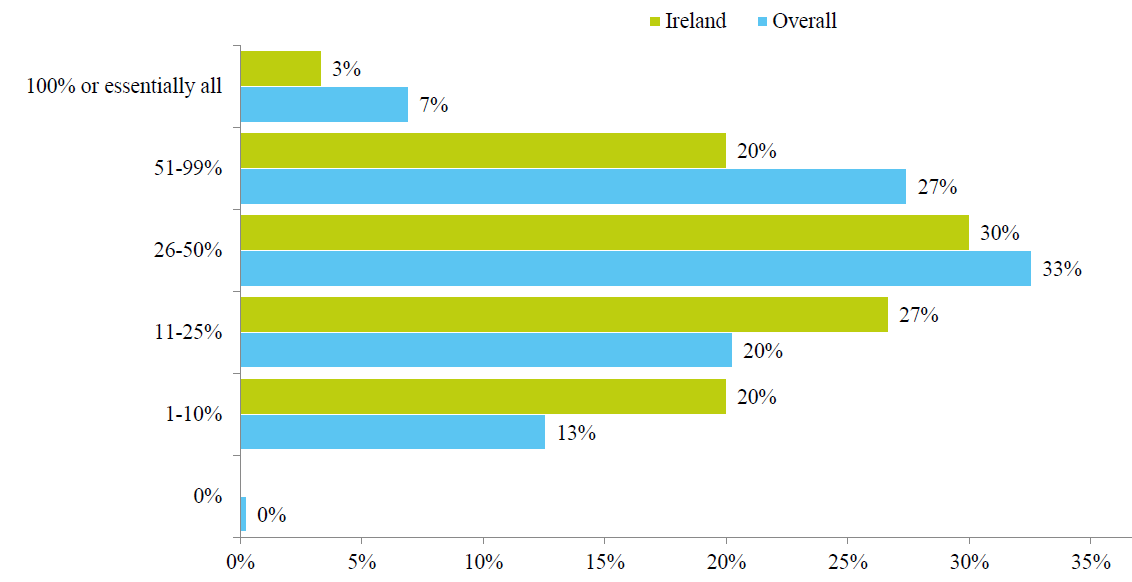


How would you respond to the following statements?


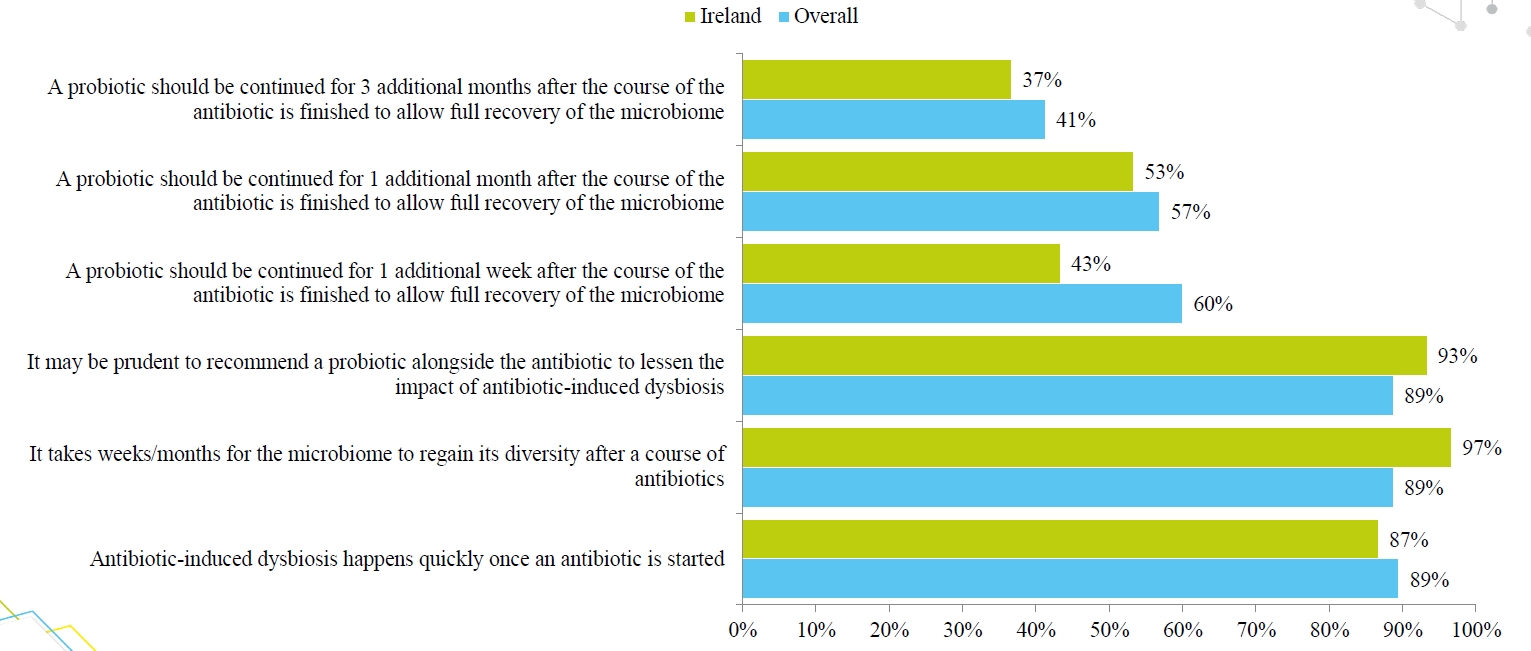


How would you respond to the following statements?


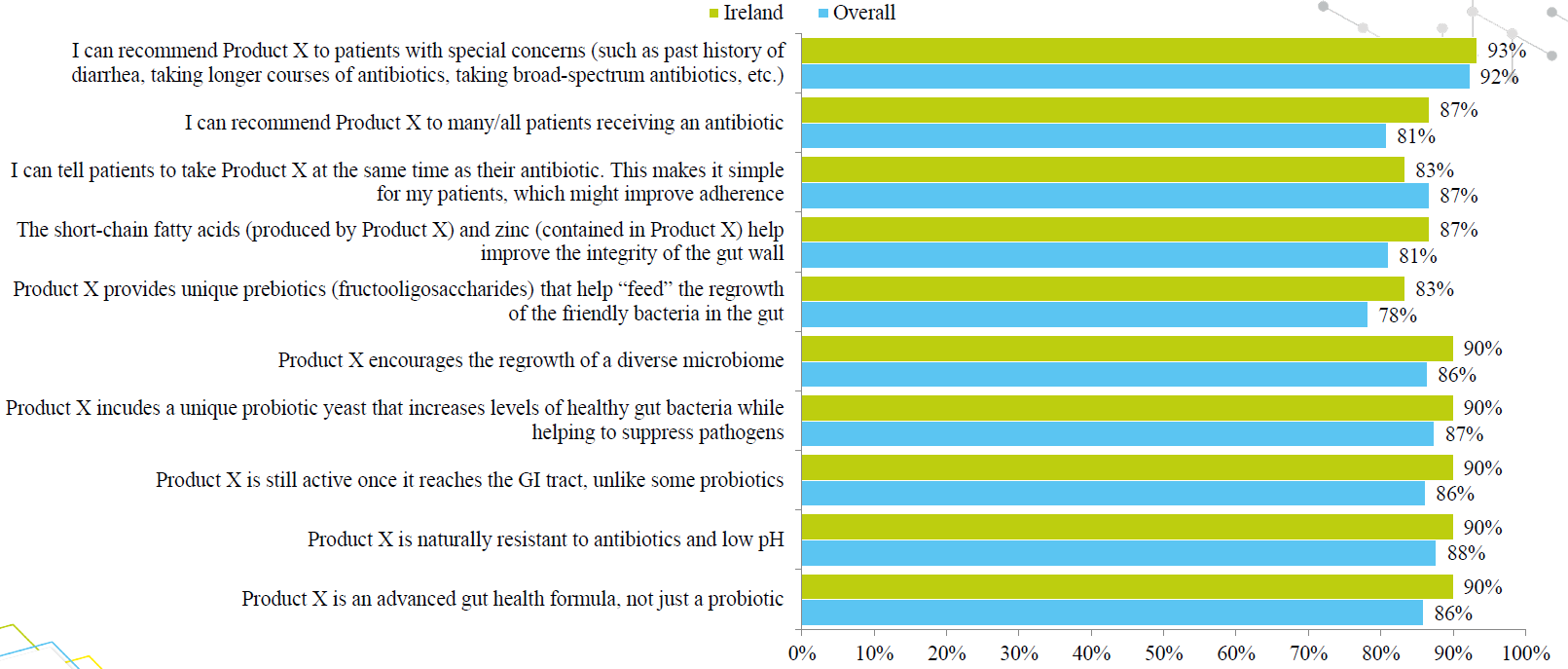


# Italy Report

In a typical week during cold and flu season, what percentage of the adult patients you see receive an antibiotic?


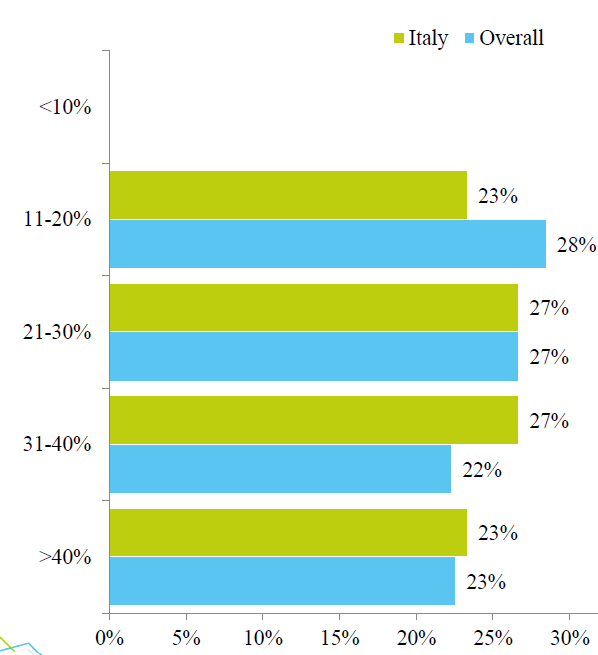


In a typical week, not during cold and flu season, what percentage of the adult patients you see receive an antibiotic?


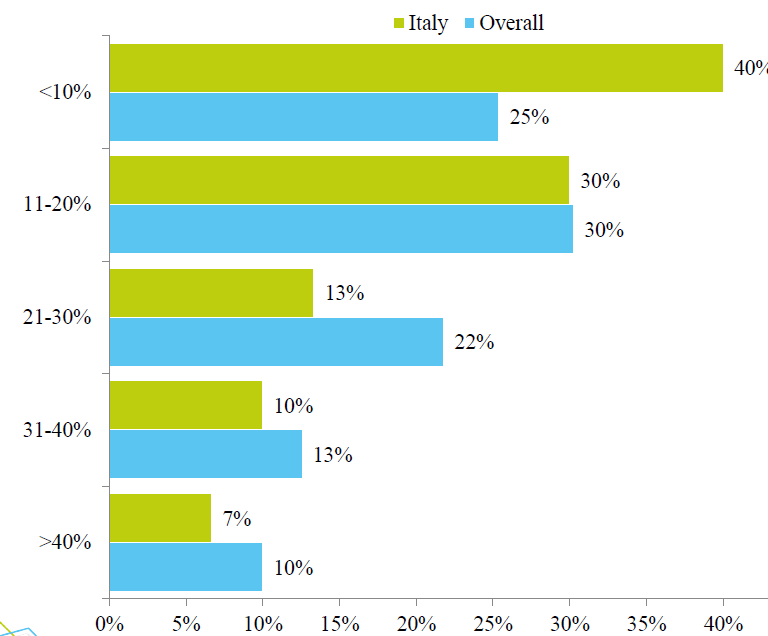


Do you see a role for probiotics when prescribing antibiotics to adult patients?


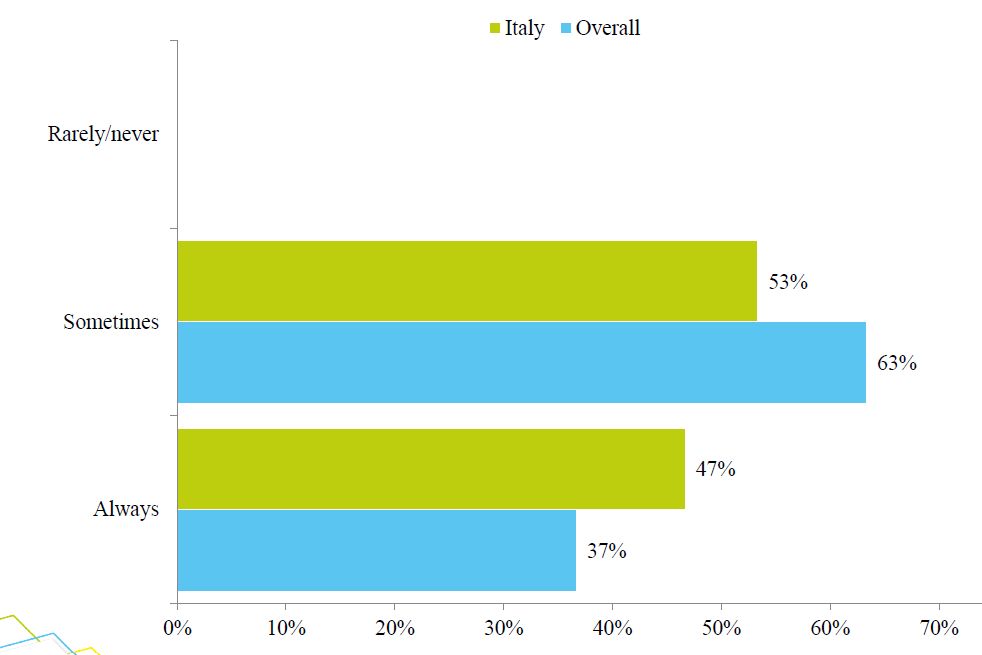


Thinking about antibiotics and the impact they can have on the microbiome, how would you respond to the following statements?


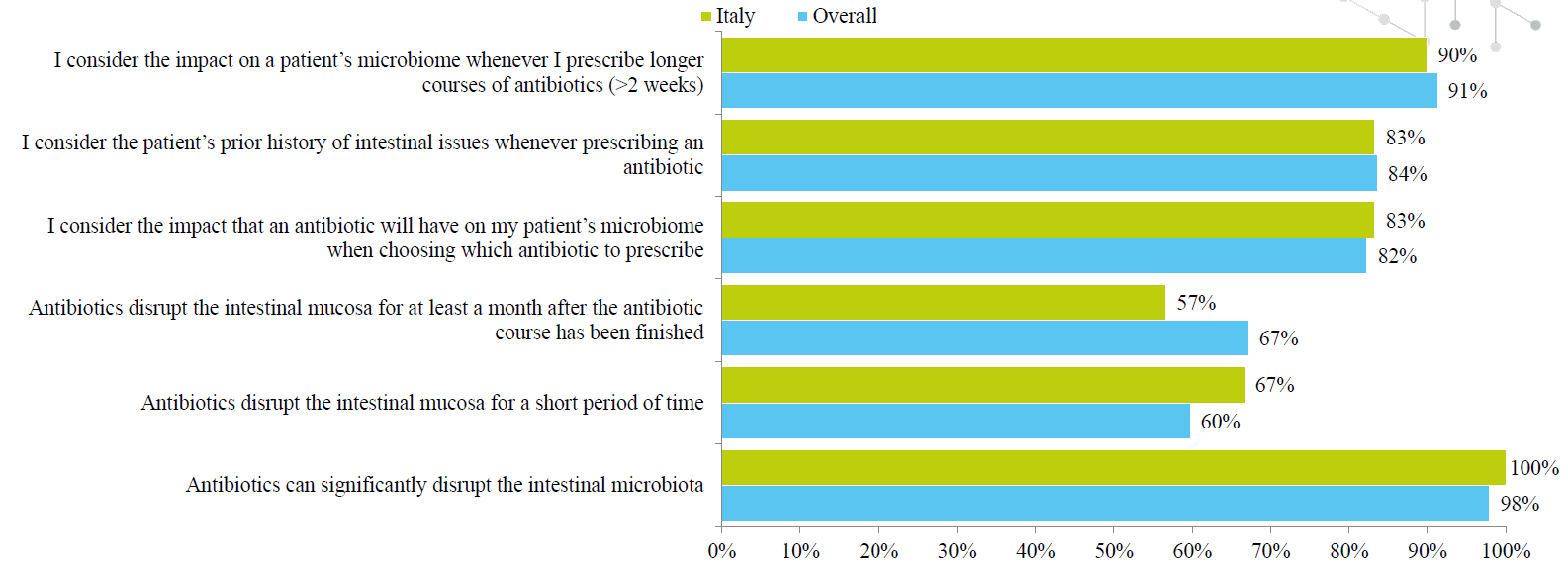


Thinking about antibiotics and the impact they can have on the microbiome, how do you respond to the following statements about probiotics?


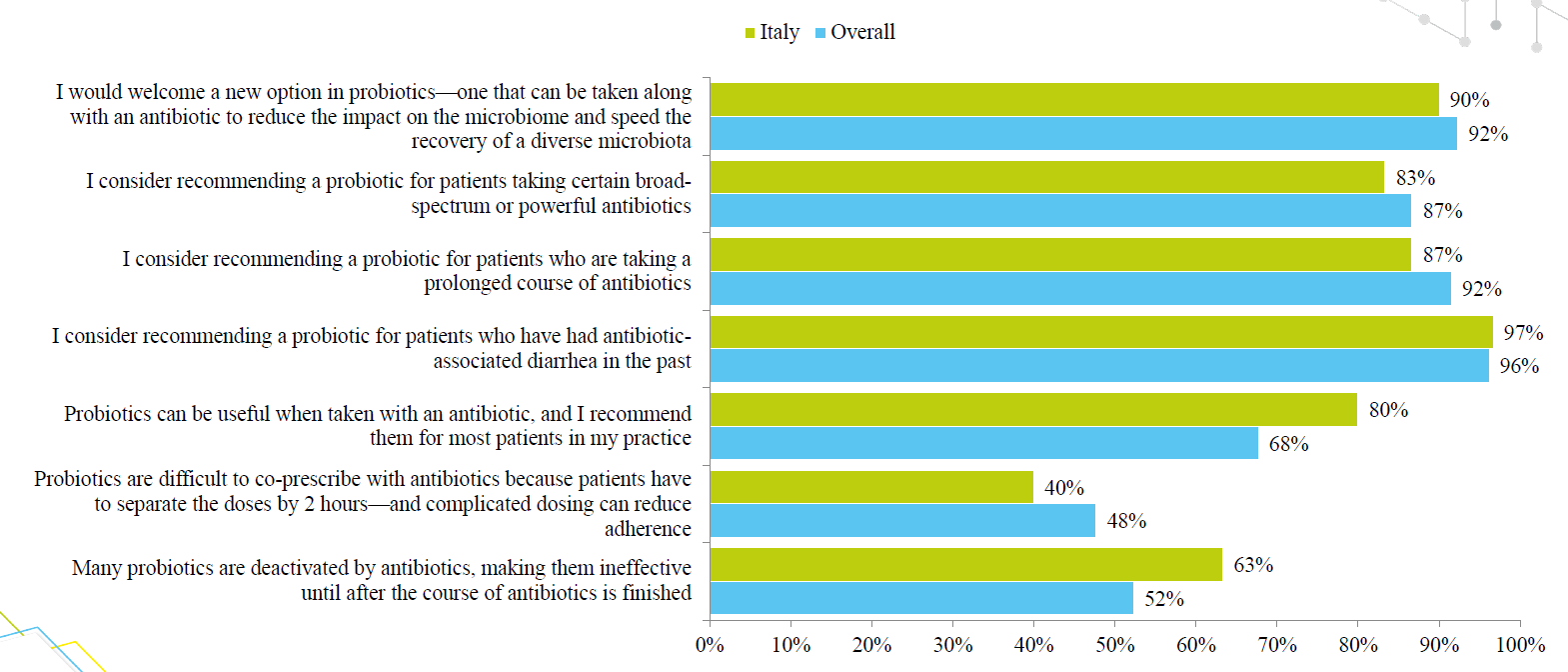


When prescribing antibiotics to adult patients today, for what percentage of patients do you also recommend probiotics?


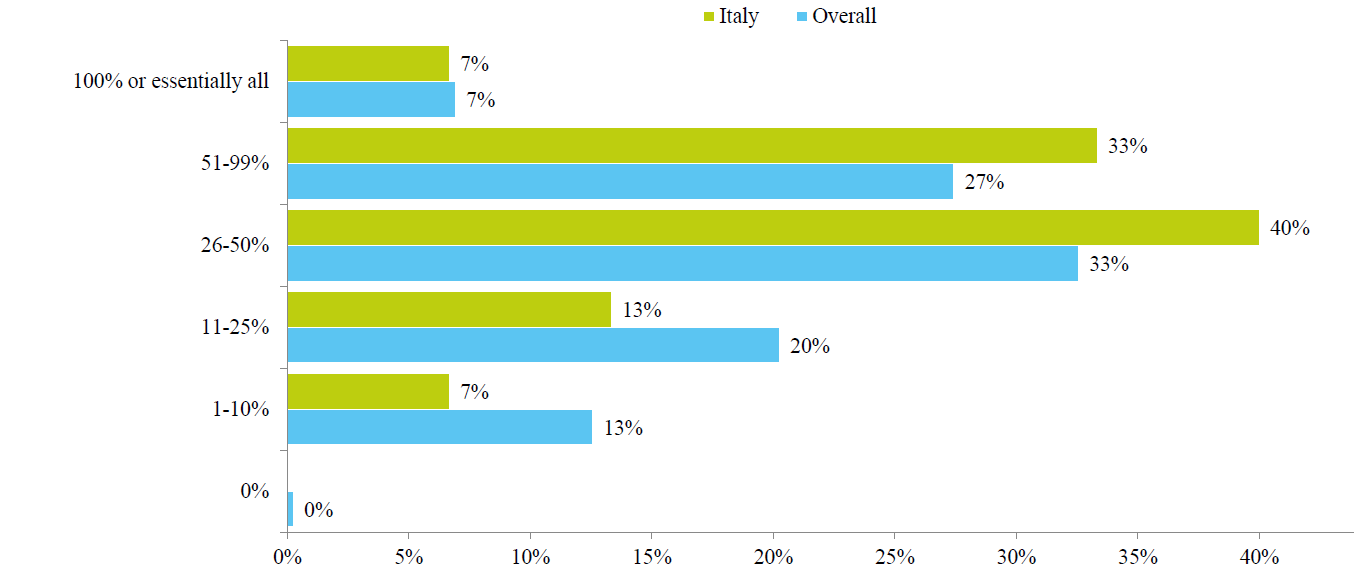


How would you respond to the following statements?


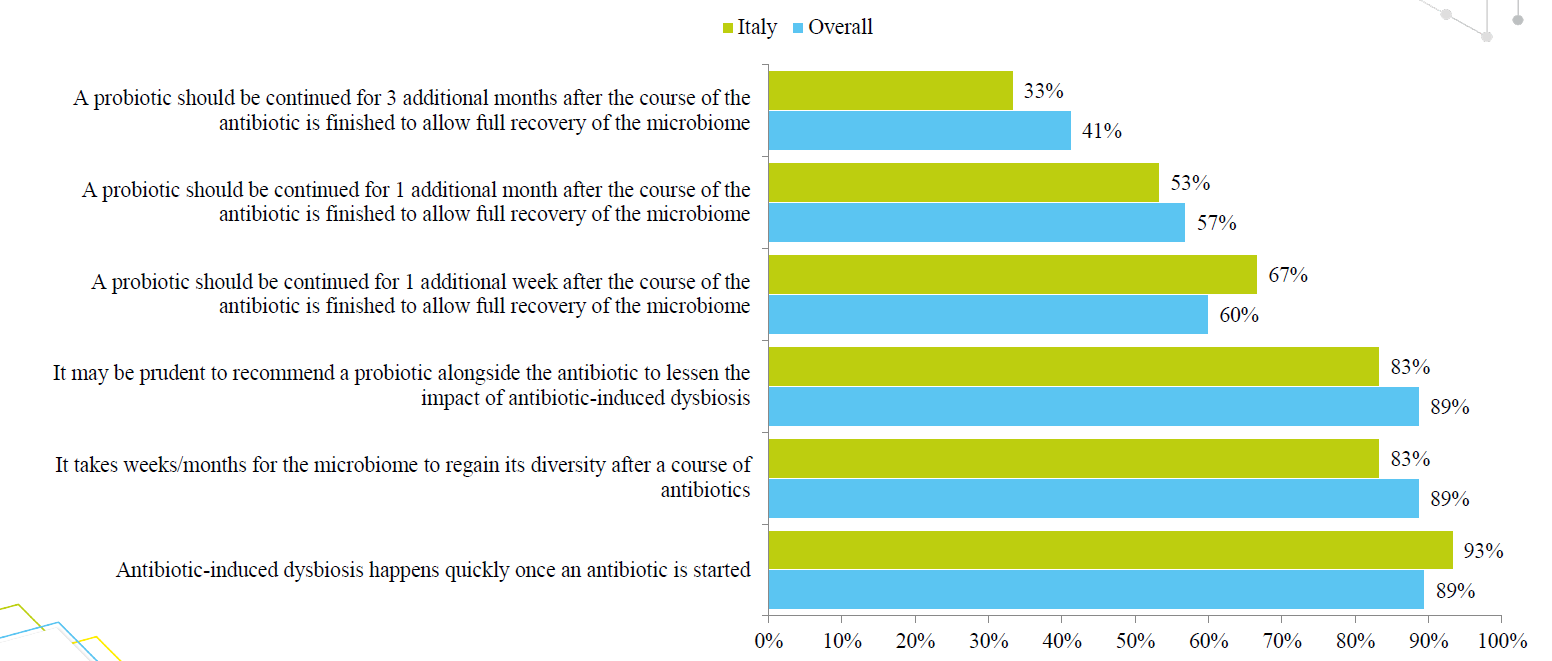


How would you respond to the following statements?


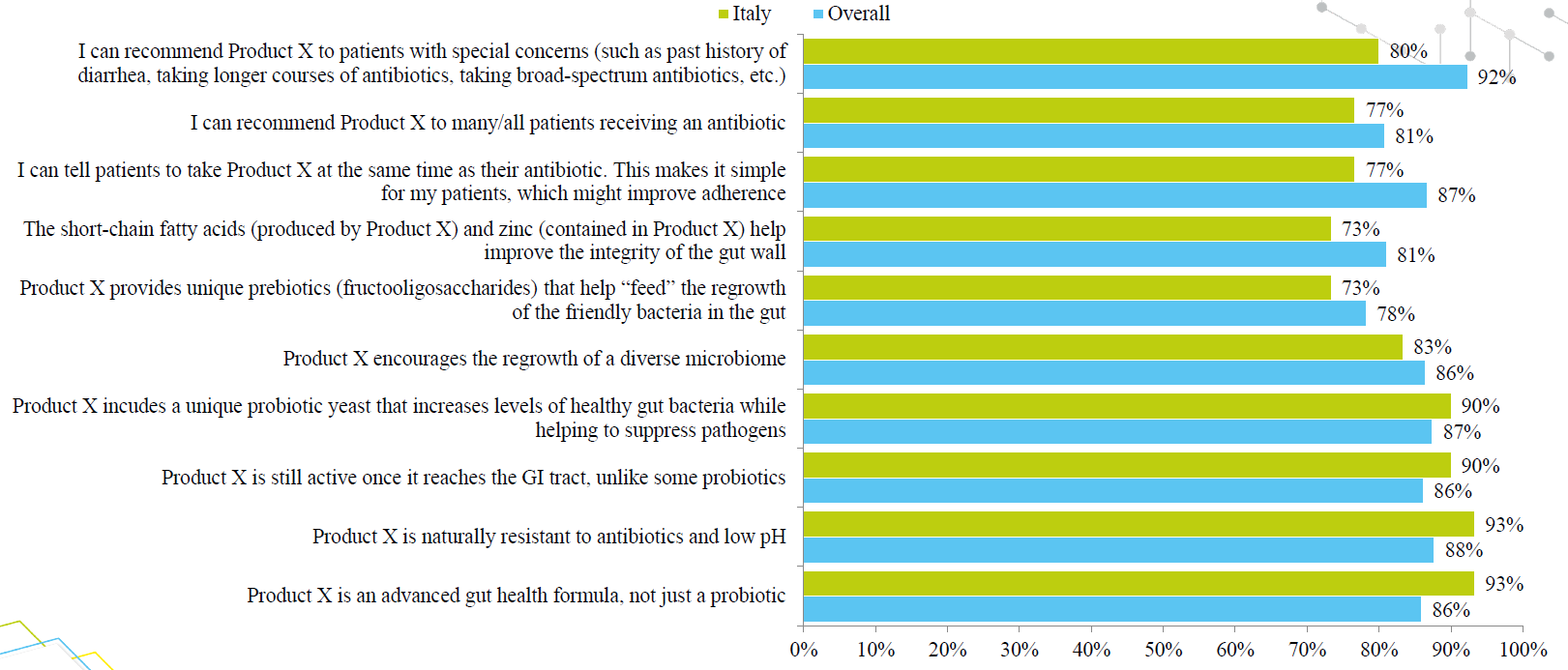


# Lithuania Report

In a typical week during cold and flu season, what percentage of the adult patients you see receive an antibiotic?


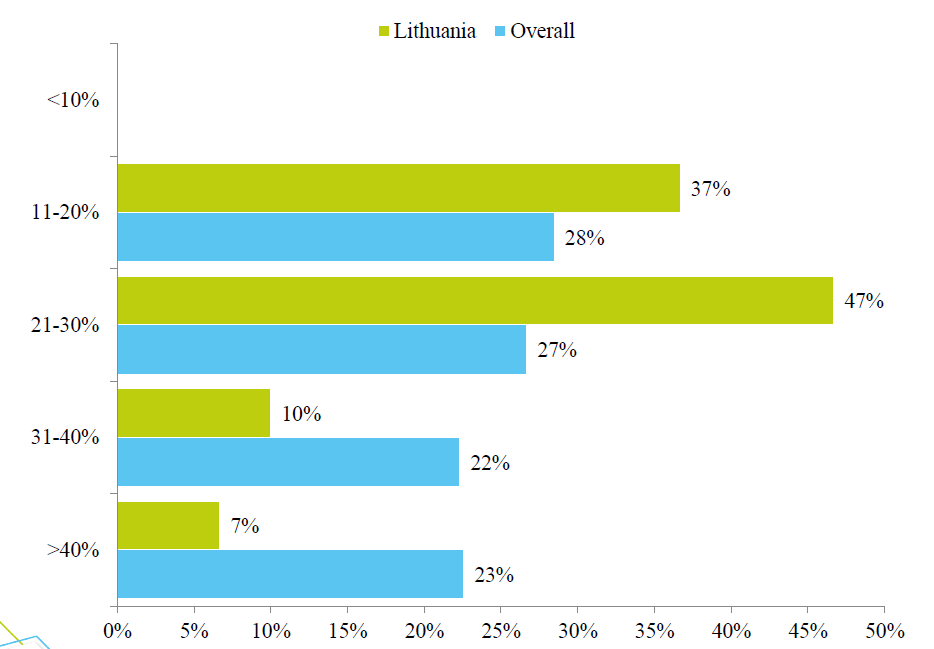


In a typical week, not during cold and flu season, what percentage of the adult patients you see receive an antibiotic?


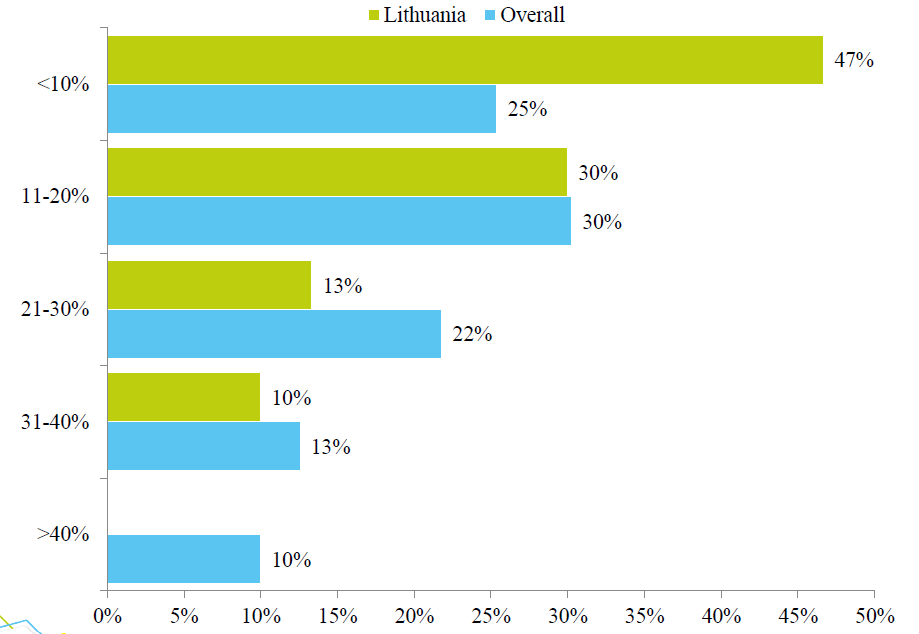


Do you see a role for probiotics when prescribing antibiotics to adult patients?


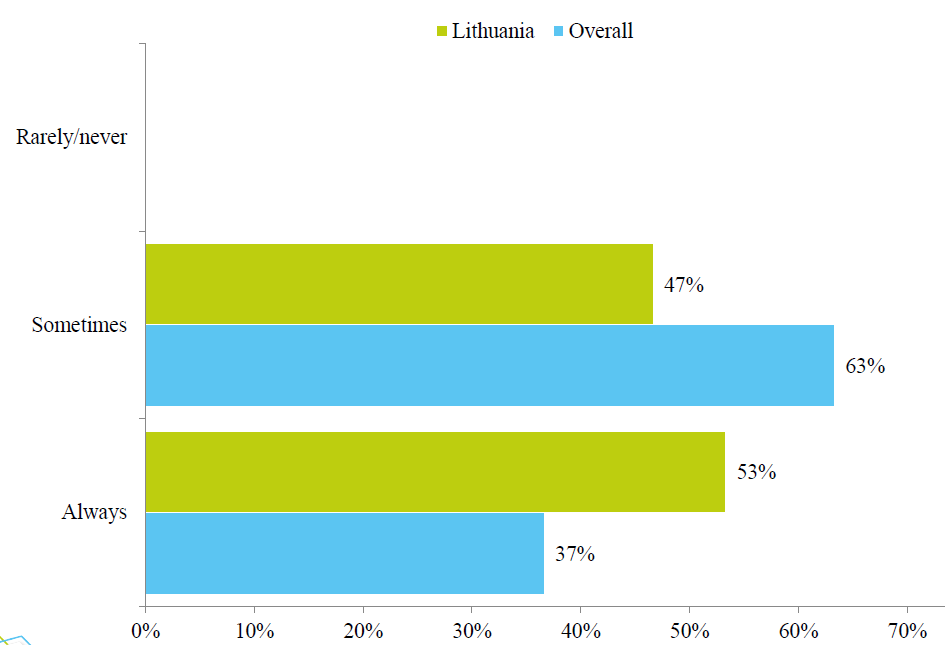


Thinking about antibiotics and the impact they can have on the microbiome, how would you respond to the following statements?


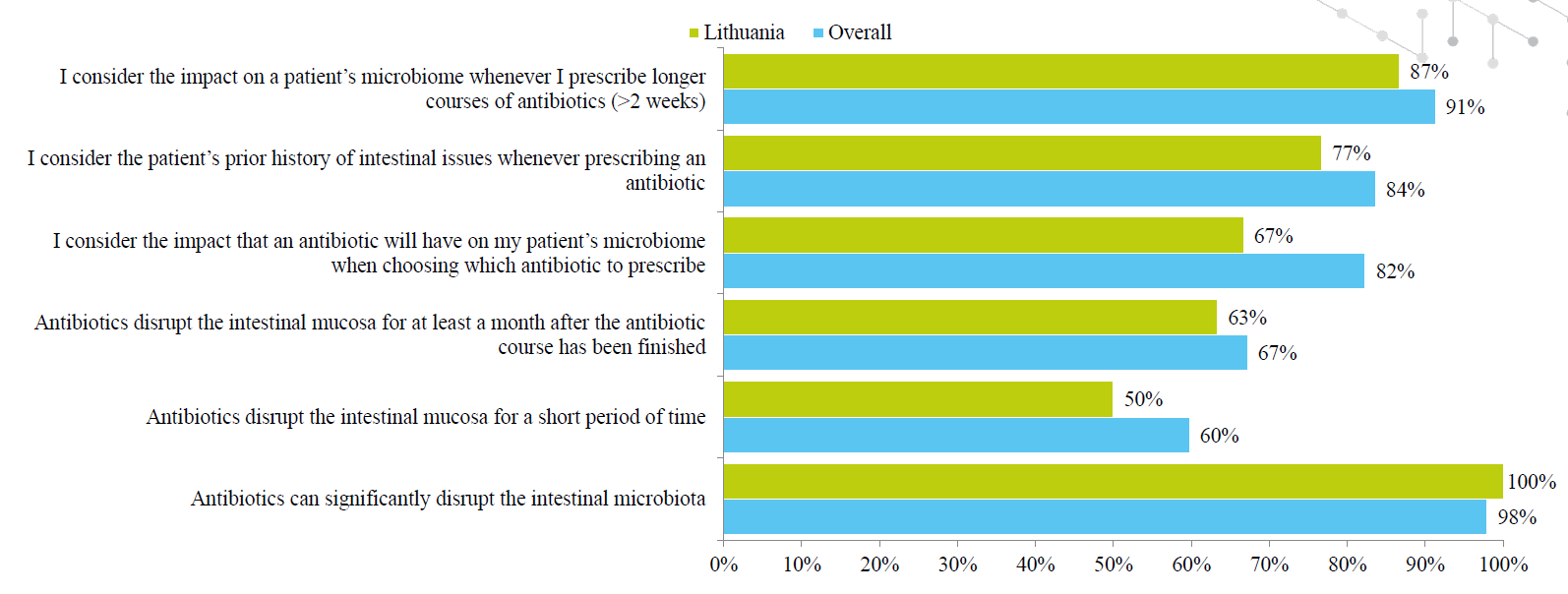


Thinking about antibiotics and the impact they can have on the microbiome, how do you respond to the following statements about probiotics?


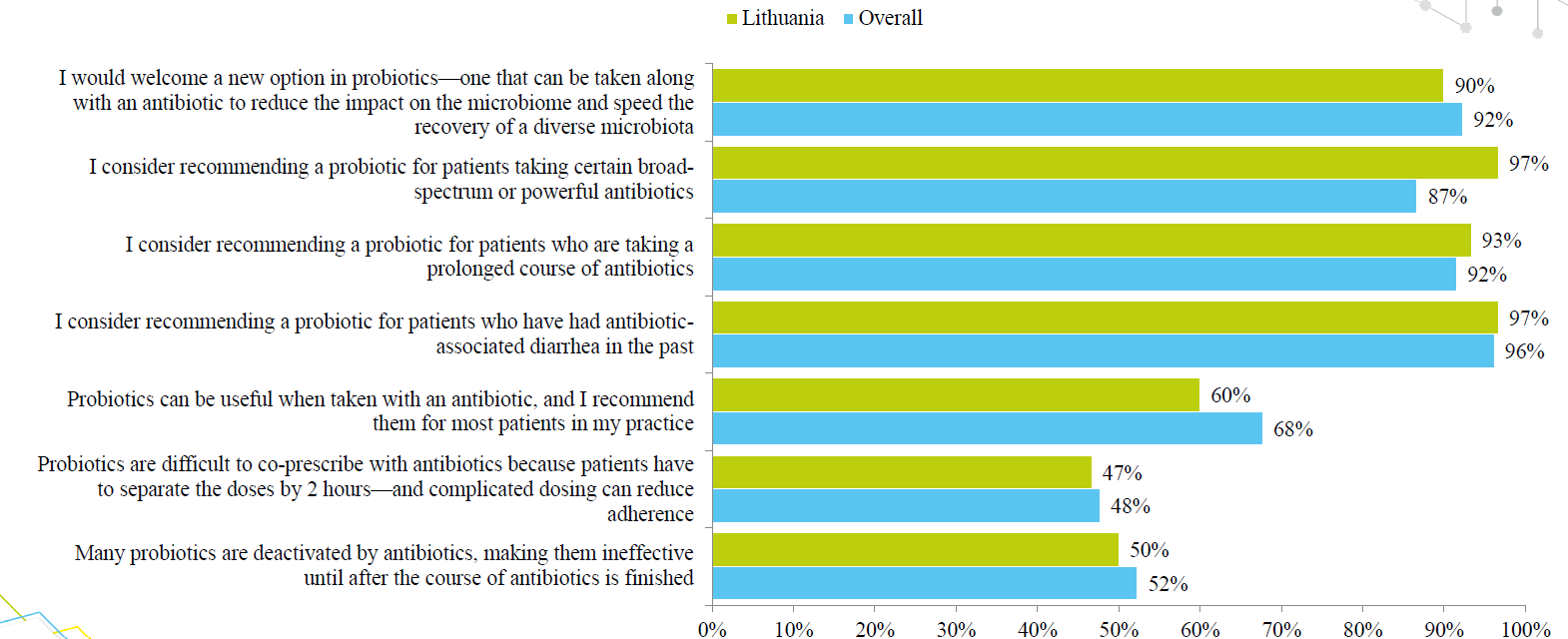


When prescribing antibiotics to adult patients today, for what percentage of patients do you also recommend probiotics?


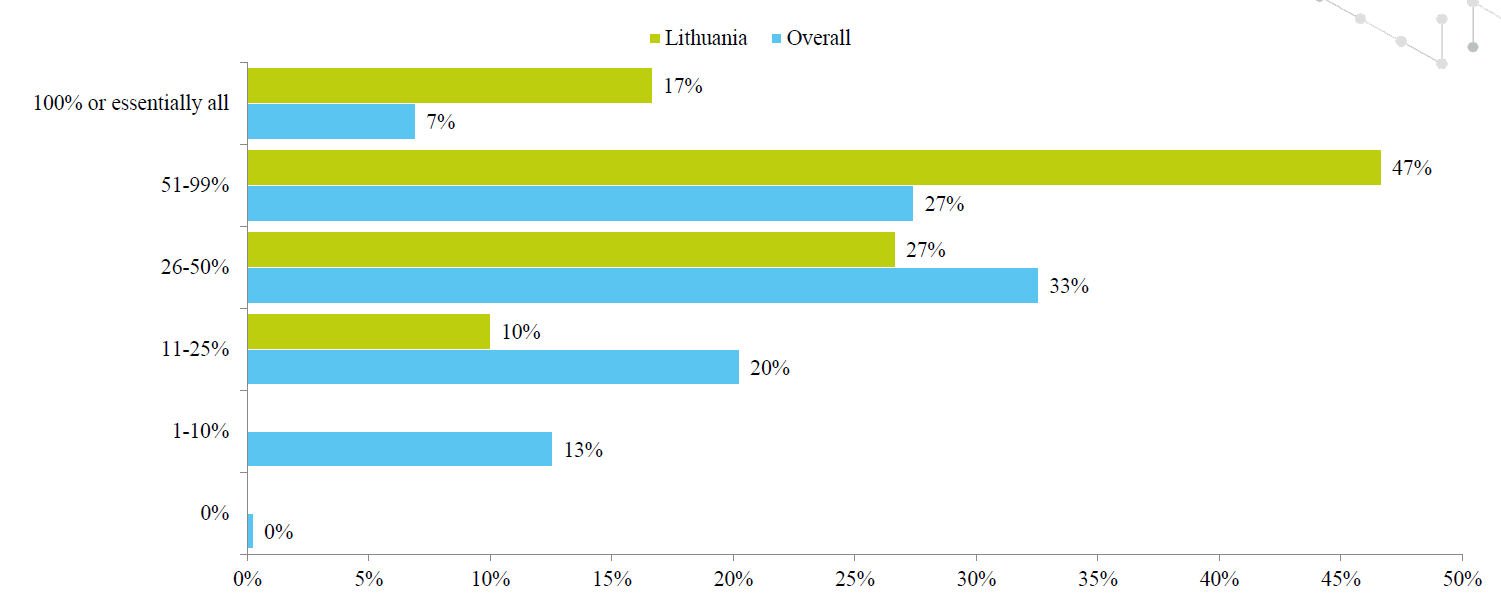


How would you respond to the following statements?


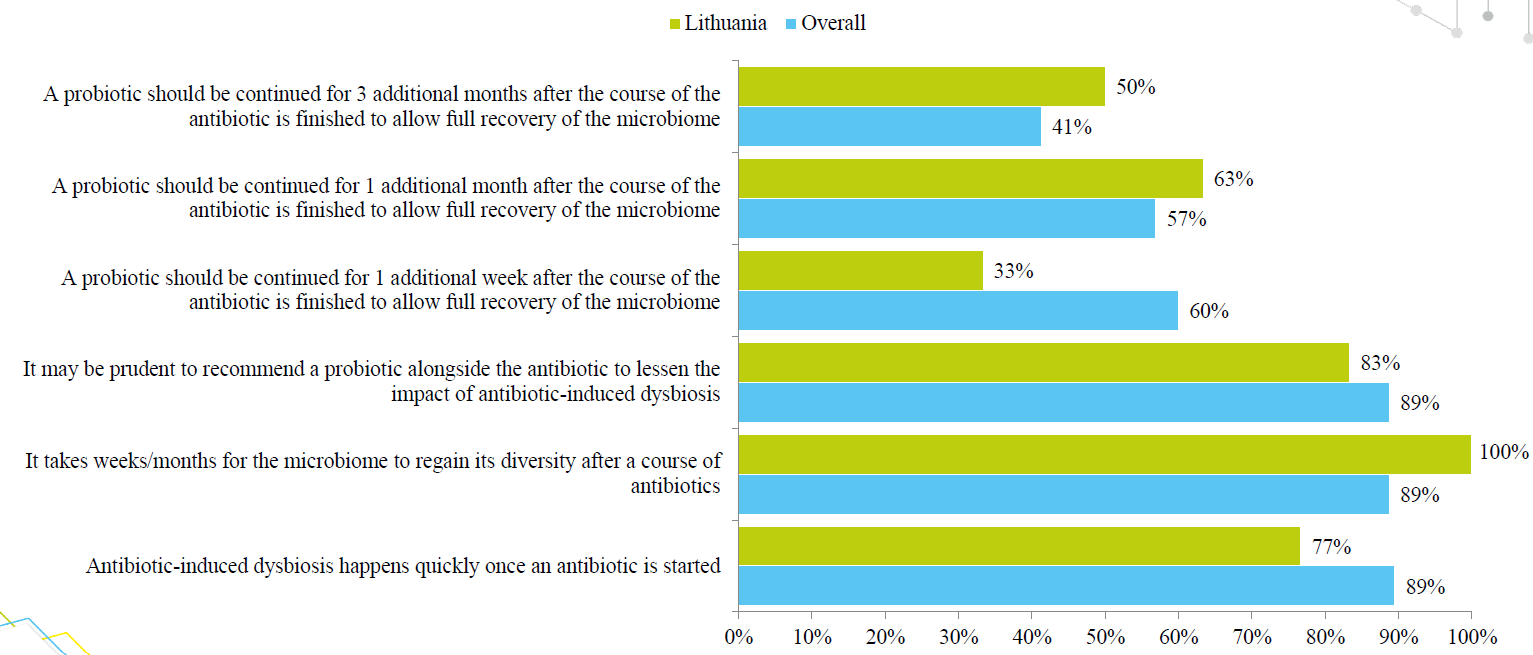


How would you respond to the following statements?


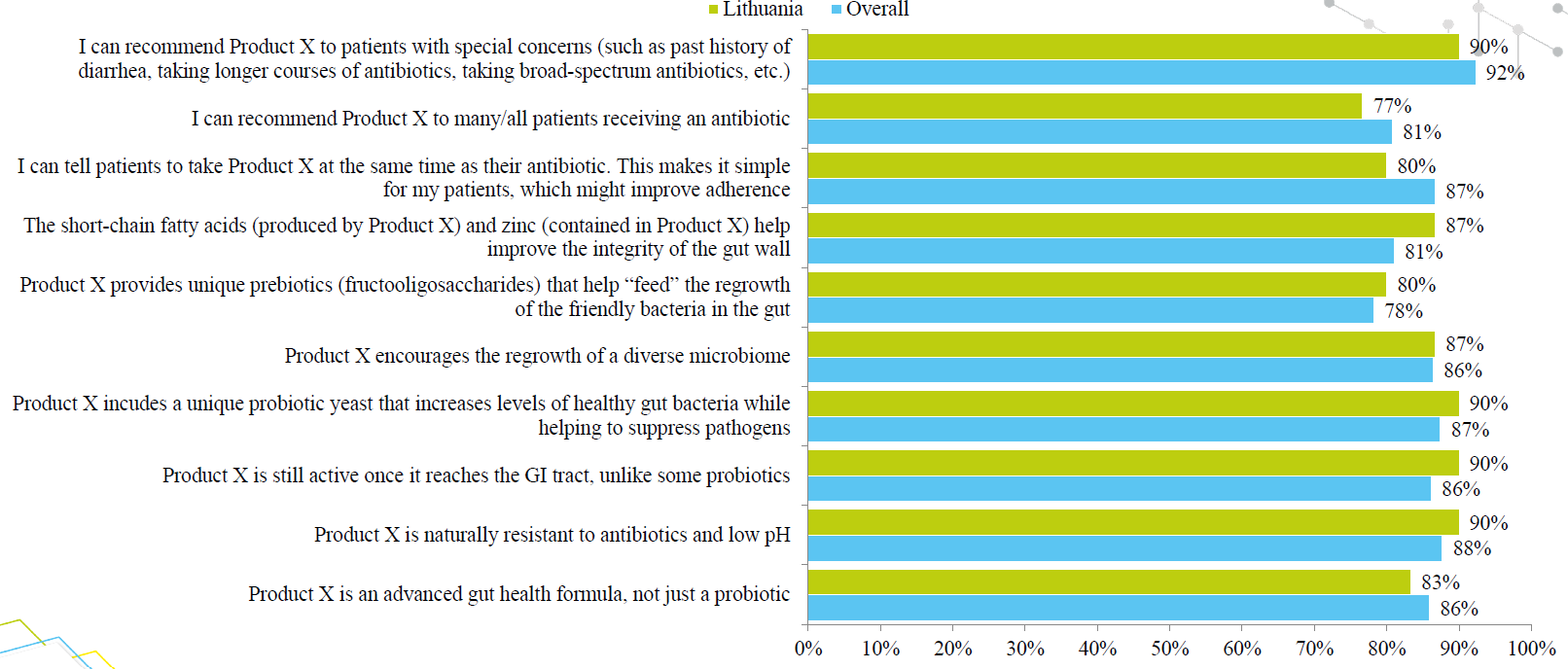


# Mexico Report

In a typical week during cold and flu season, what percentage of the adult patients you see receive an antibiotic?


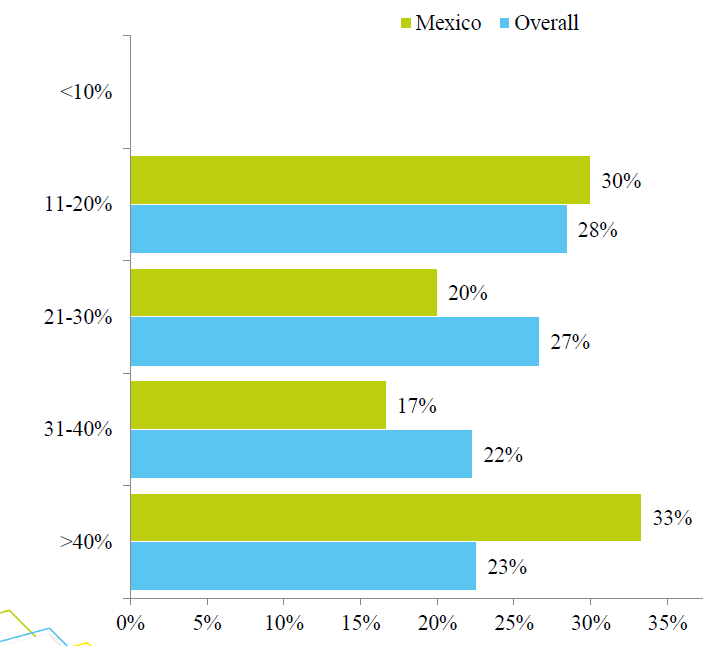


In a typical week, not during cold and flu season, what percentage of the adult patients you see receive an antibiotic?


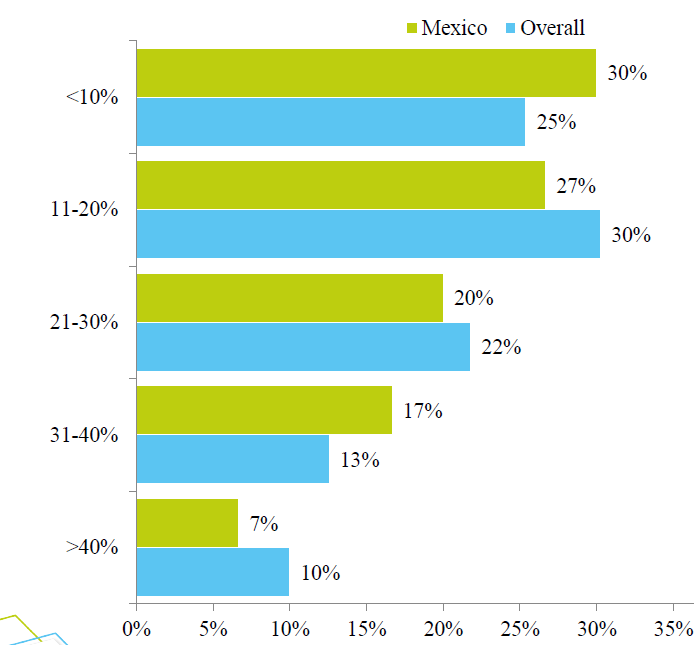


Do you see a role for probiotics when prescribing antibiotics to adult patients?


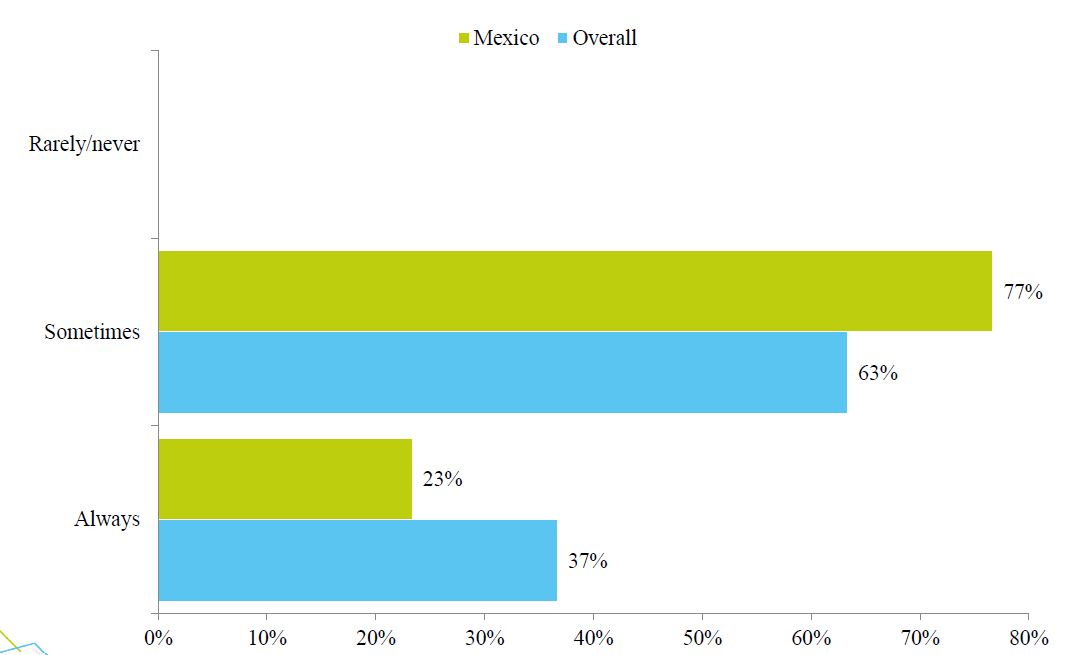


Thinking about antibiotics and the impact they can have on the microbiome, how would you respond to the following statements?


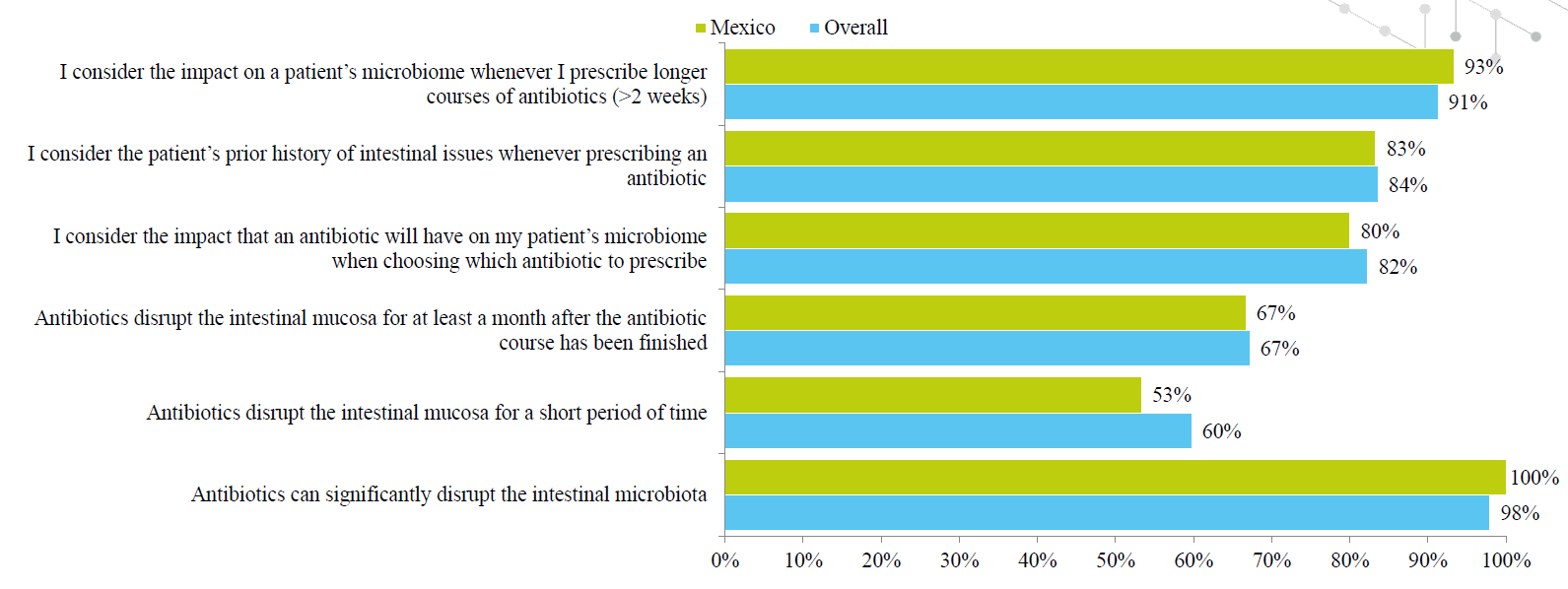


Thinking about antibiotics and the impact they can have on the microbiome, how do you respond to the following statements about probiotics?

When prescribing antibiotics to adult patients today, for what percentage of patients do you also recommend probiotics?


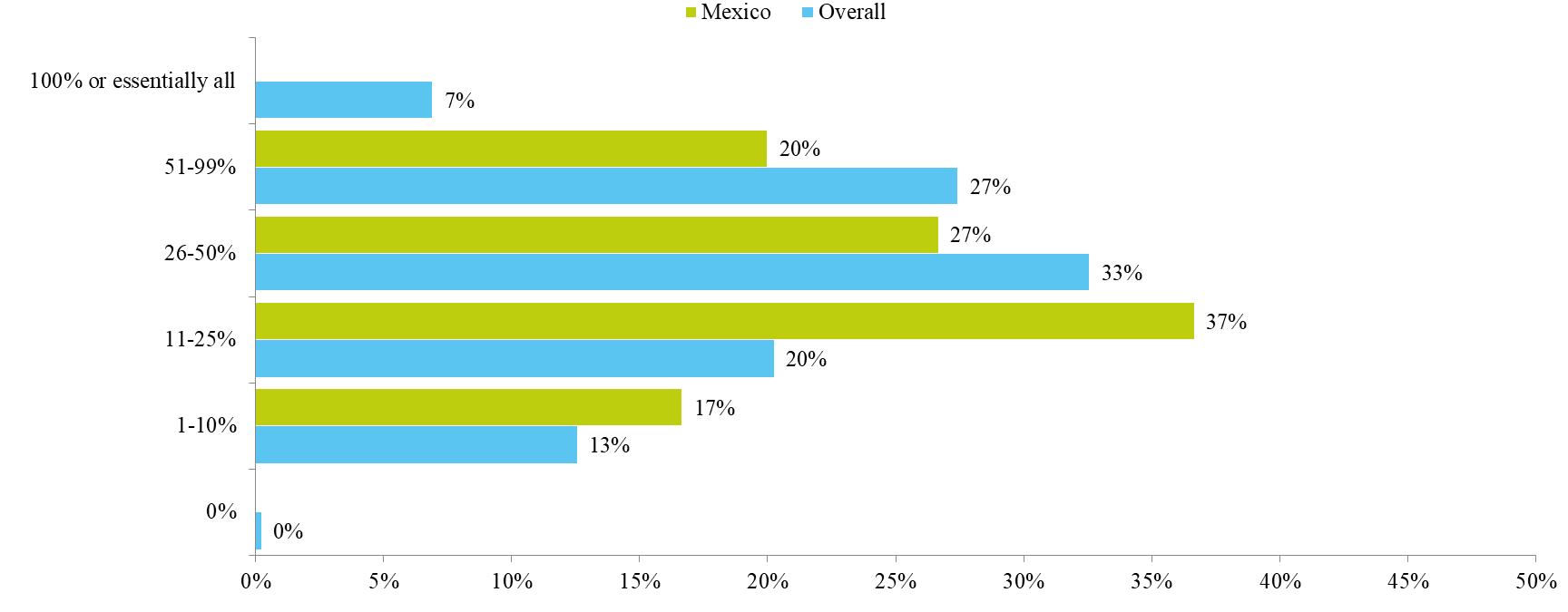


How would you respond to the following statements?


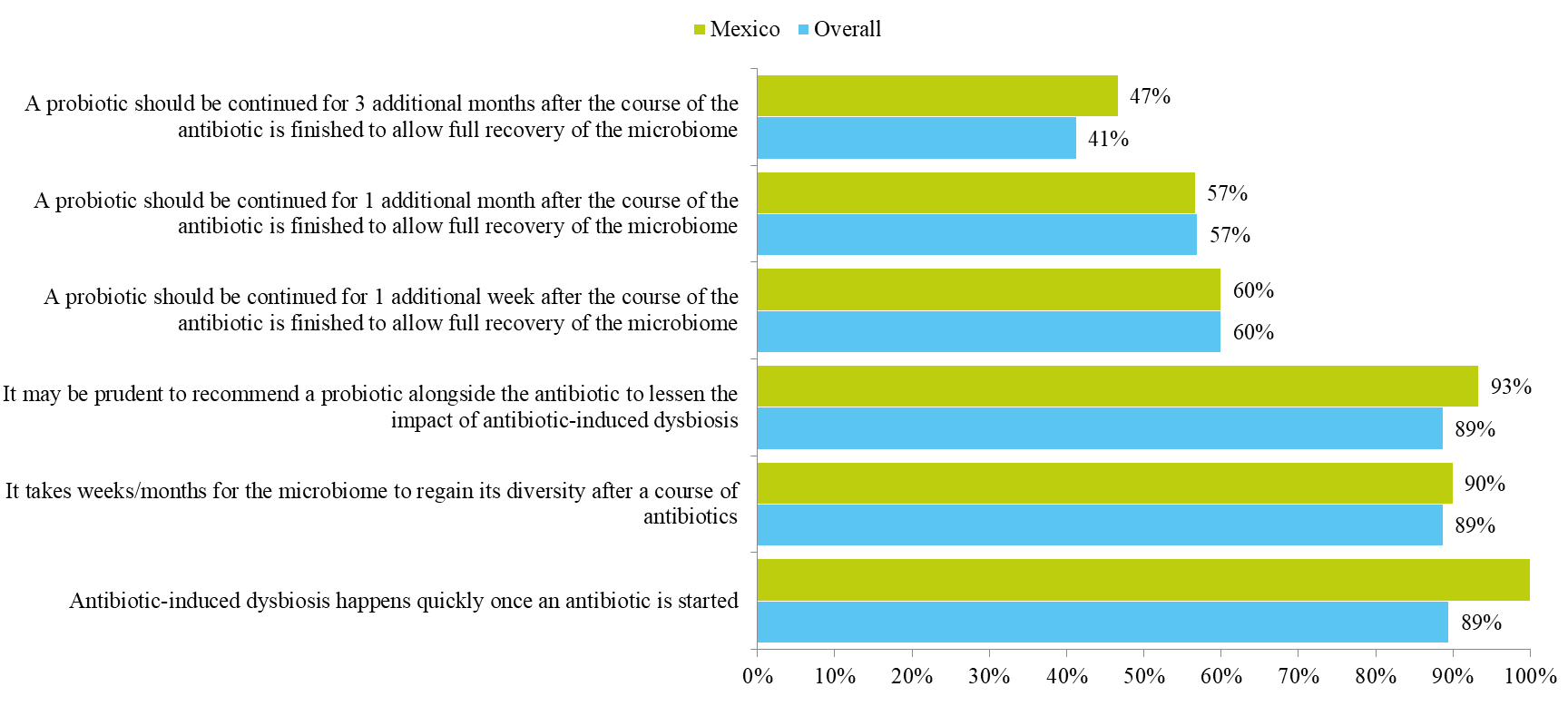


How would you respond to the following statements?


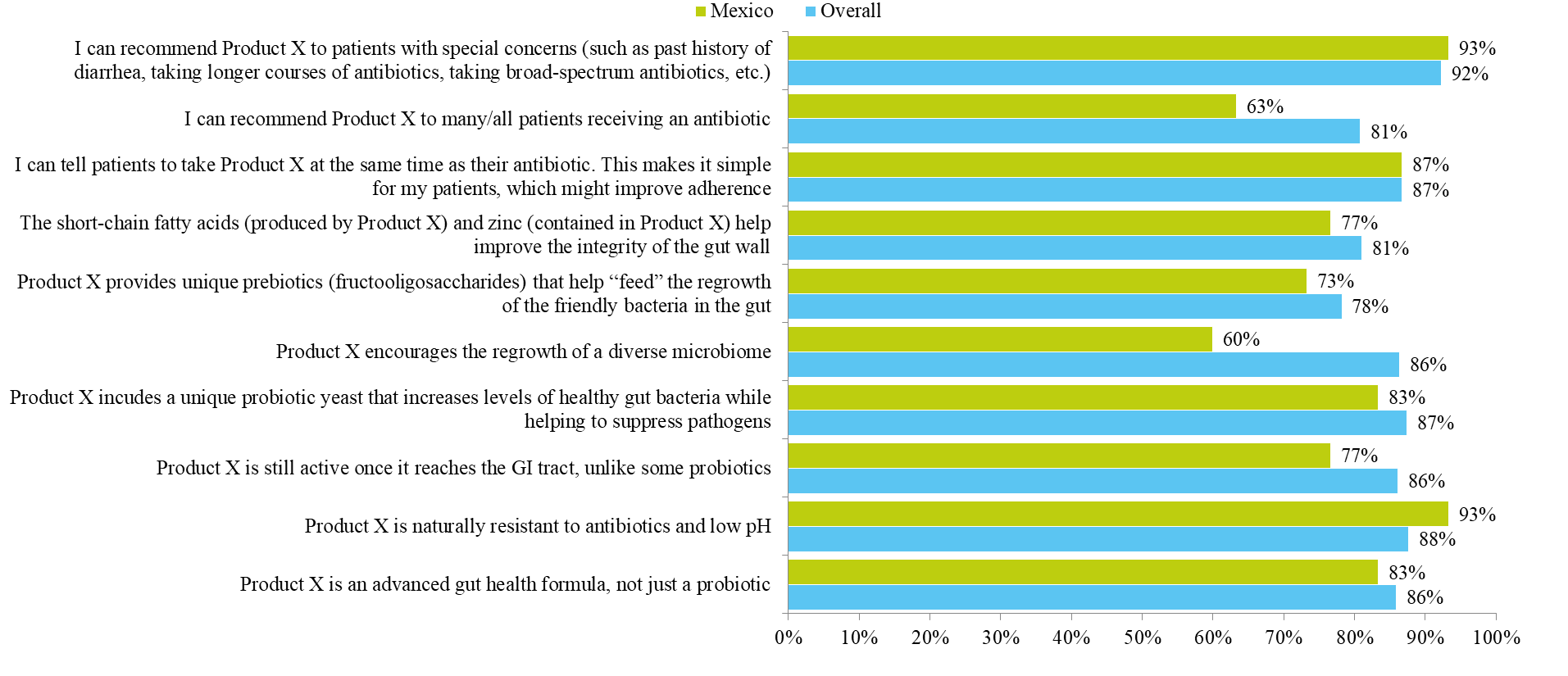


# Peru Report

In a typical week during cold and flu season, what percentage of the adult patients you see receive an antibiotic?


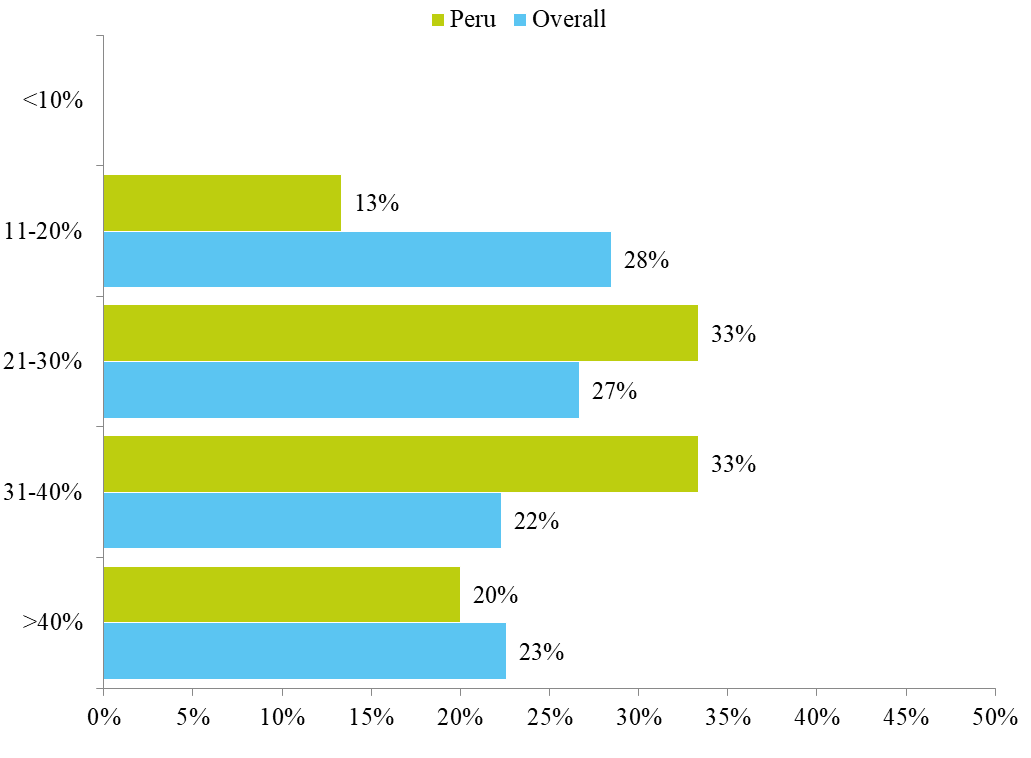


In a typical week, not during cold and flu season, what percentage of the adult patients you see receive an antibiotic?


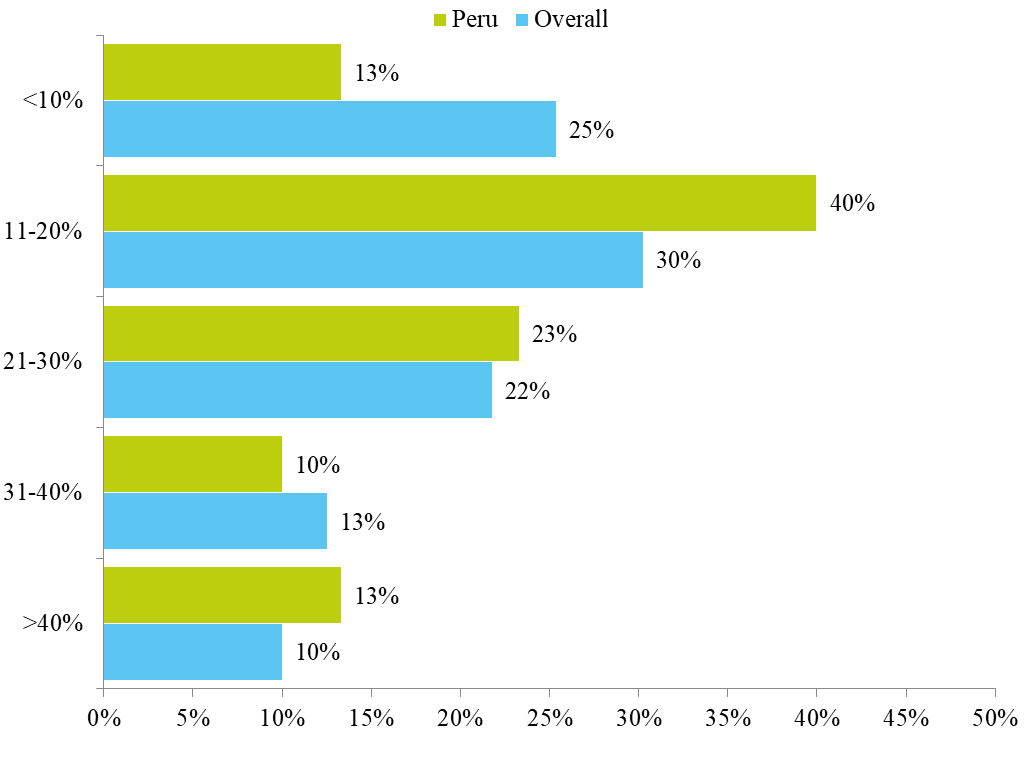


Do you see a role for probiotics when prescribing antibiotics to adult patients?


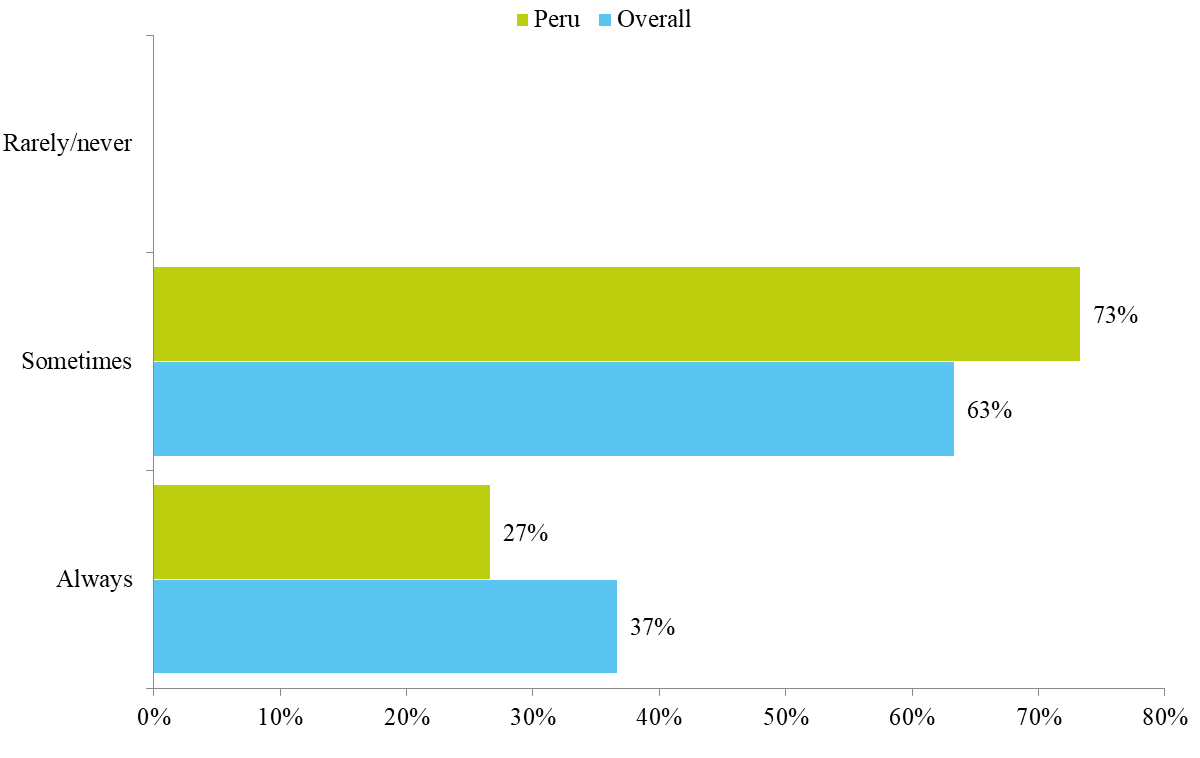


Thinking about antibiotics and the impact they can have on the microbiome, how would you respond to the following statements?


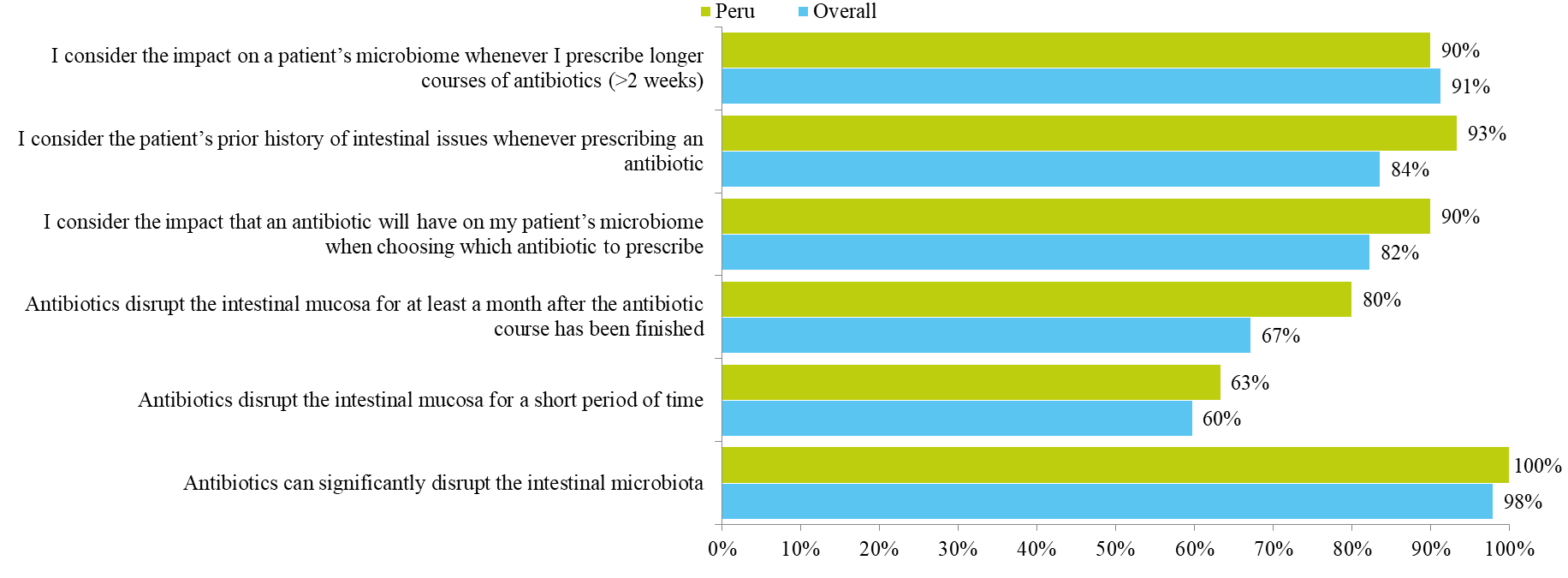


Thinking about antibiotics and the impact they can have on the microbiome, how do you respond to the following statements about probiotics?


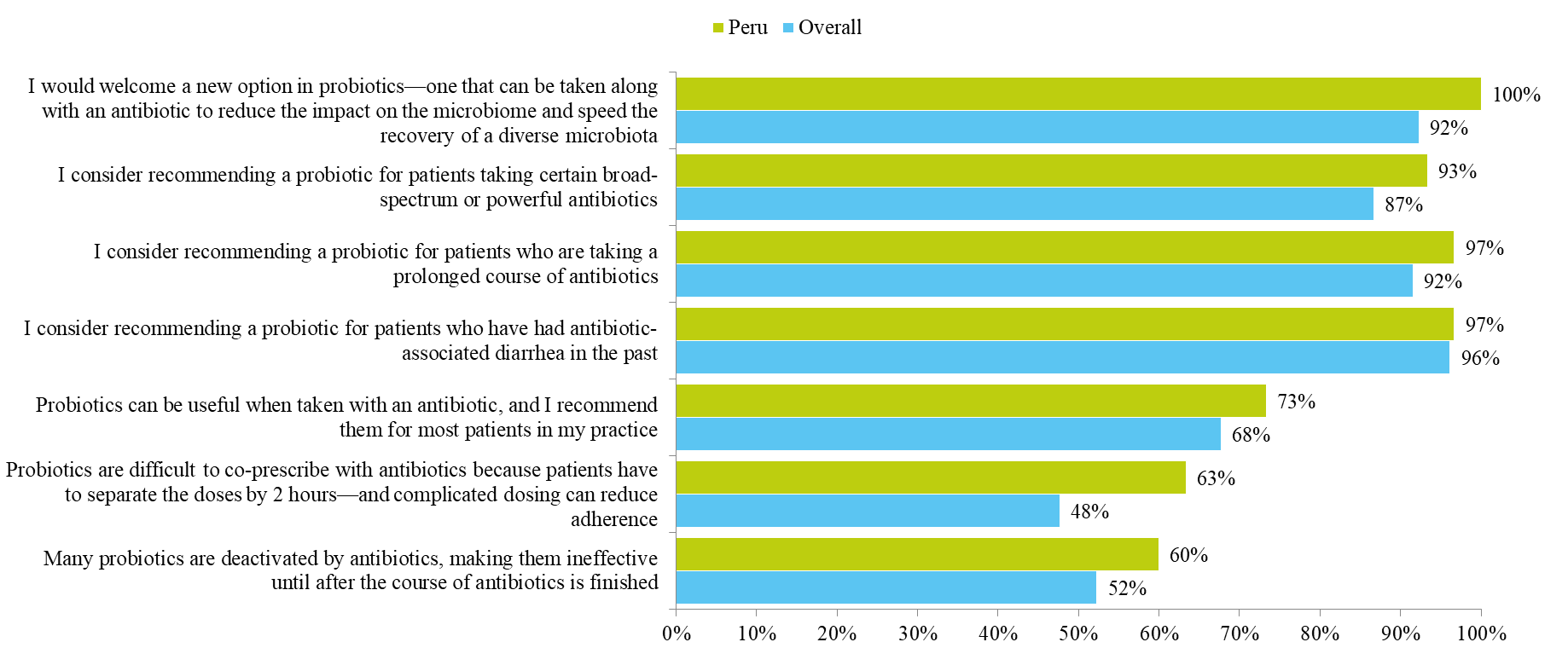


When prescribing antibiotics to adult patients today, for what percentage of patients do you also recommend probiotics?


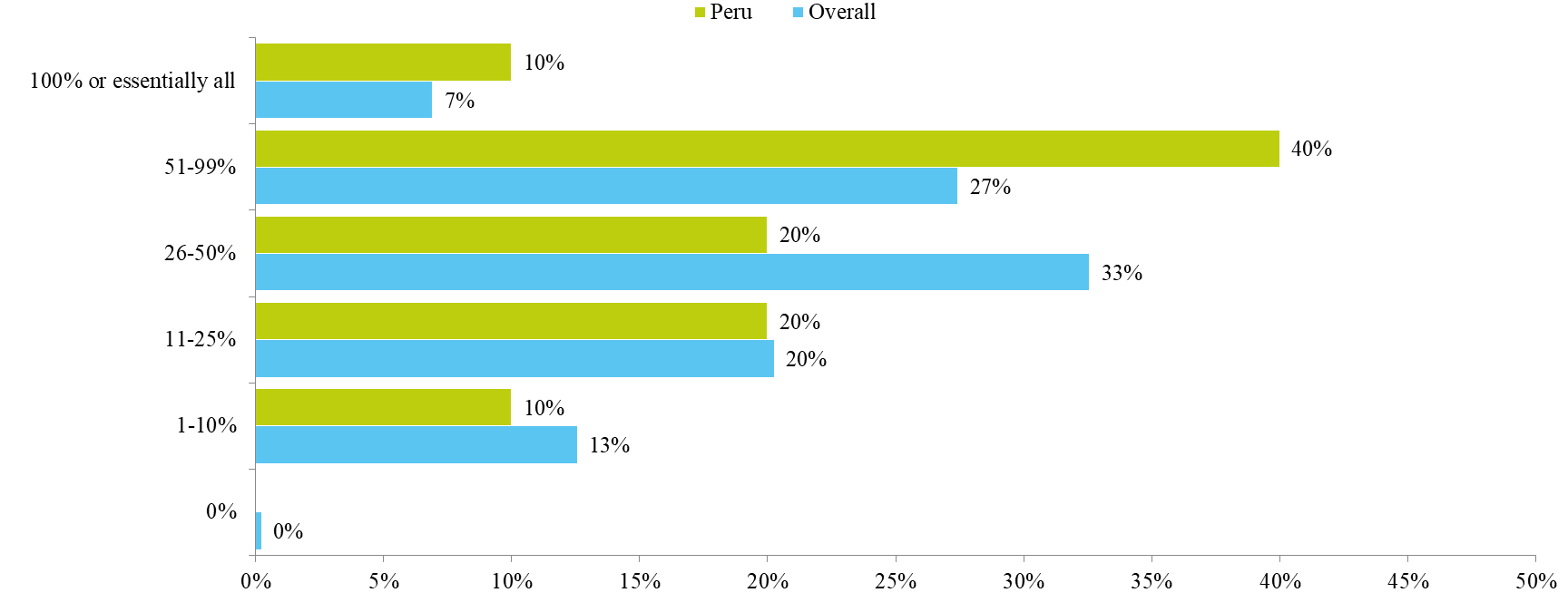


How would you respond to the following statements?


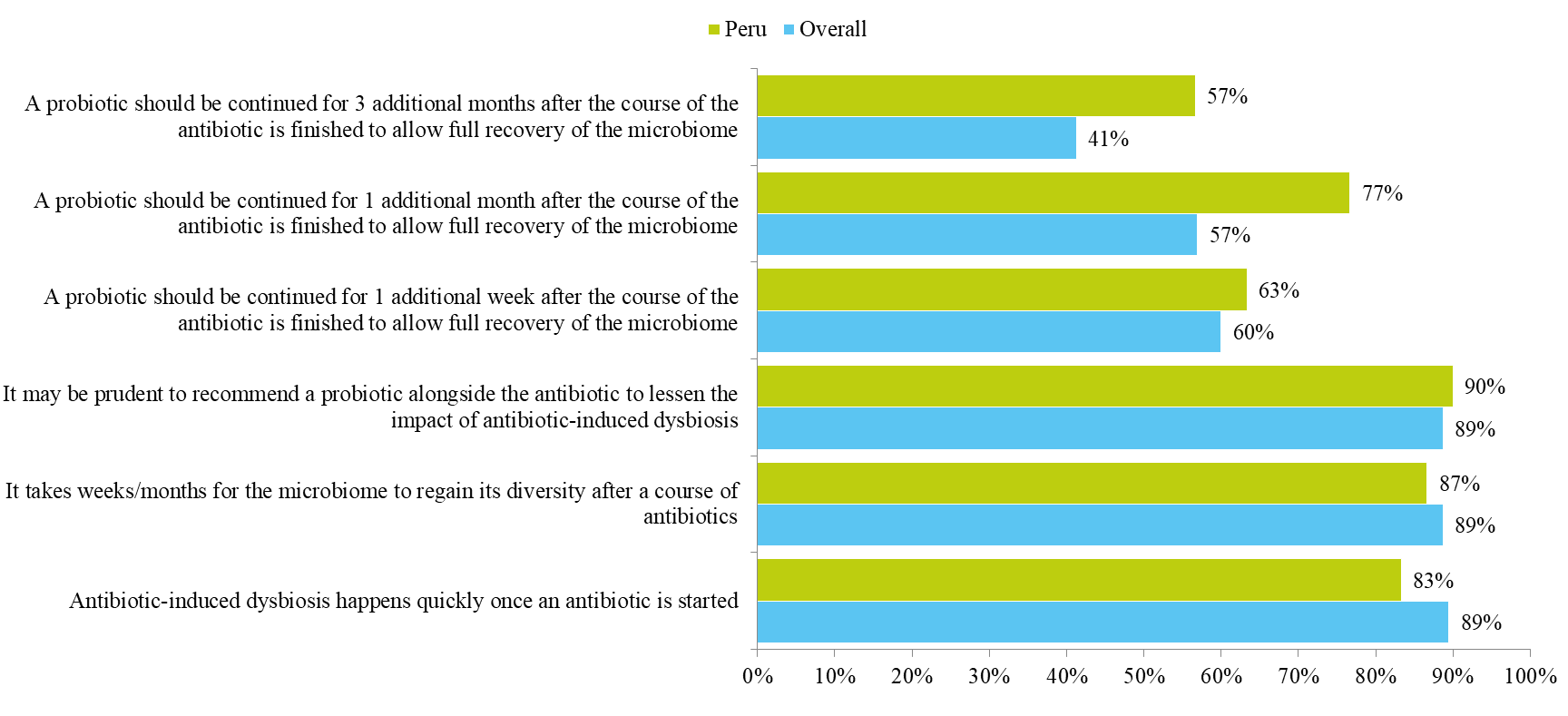


How would you respond to the following statements?


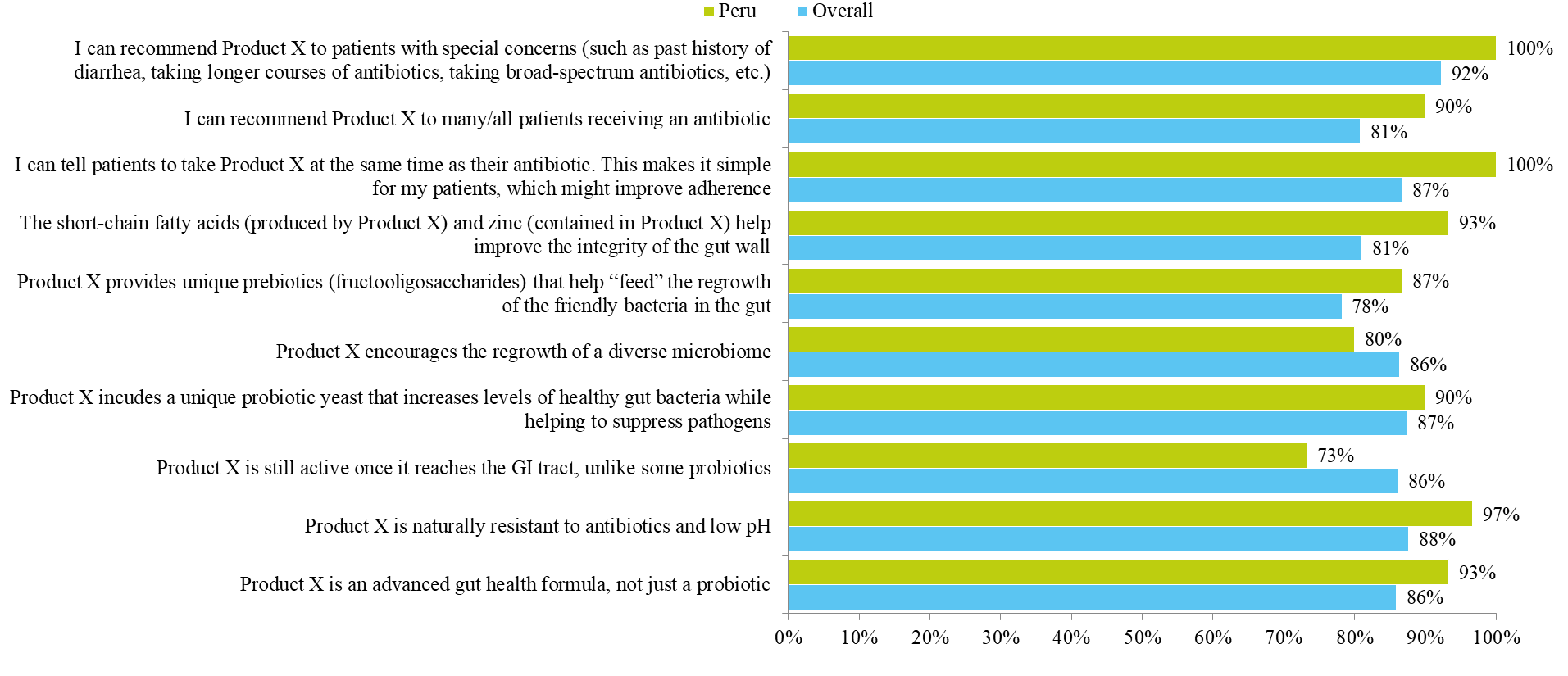


# Saudi Arabia Report

In a typical week during cold and flu season, what percentage of the adult patients you see receive an antibiotic?


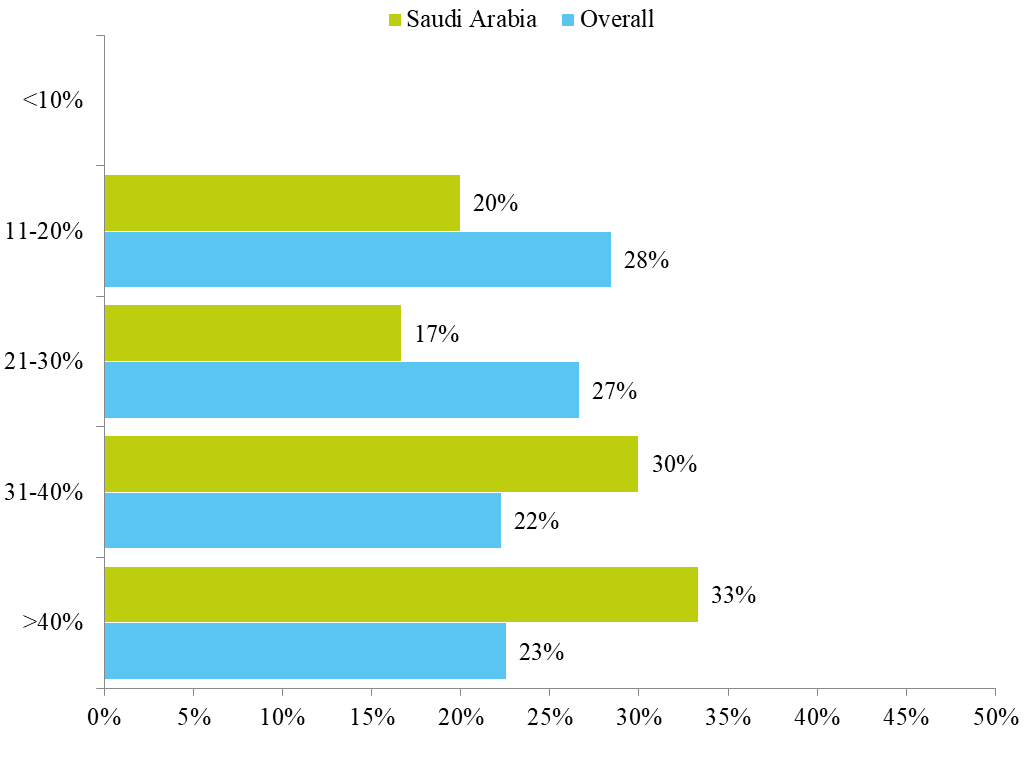


In a typical week, not during cold and flu season, what percentage of the adult patients you see receive an antibiotic?


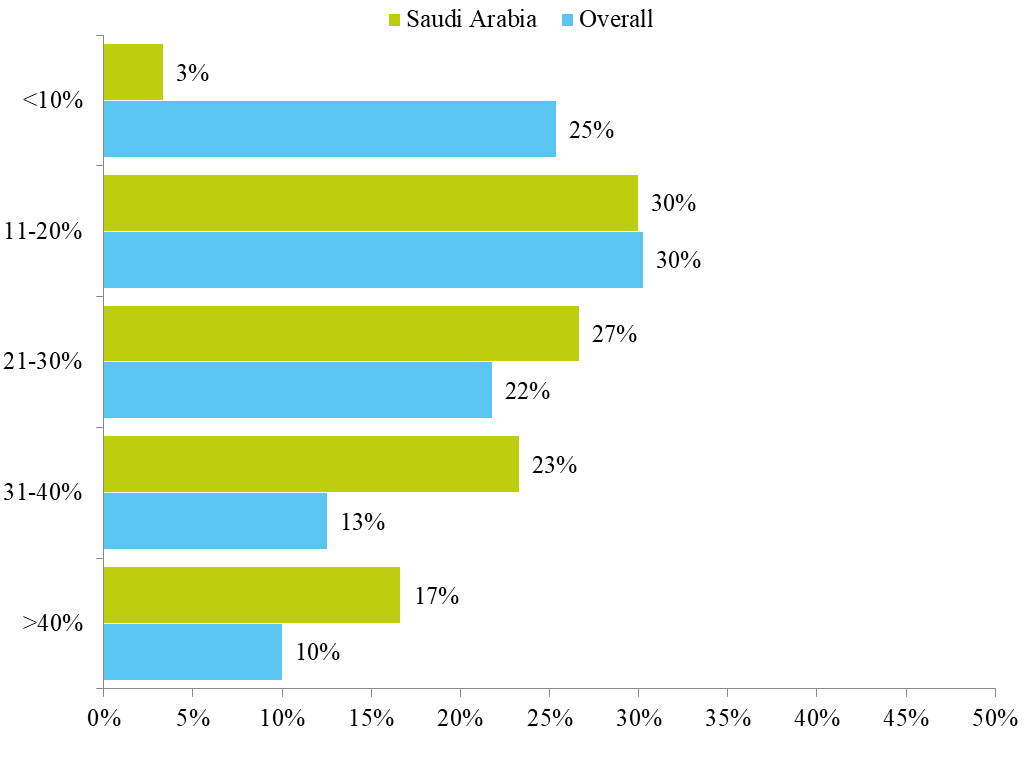


Do you see a role for probiotics when prescribing antibiotics to adult patients?


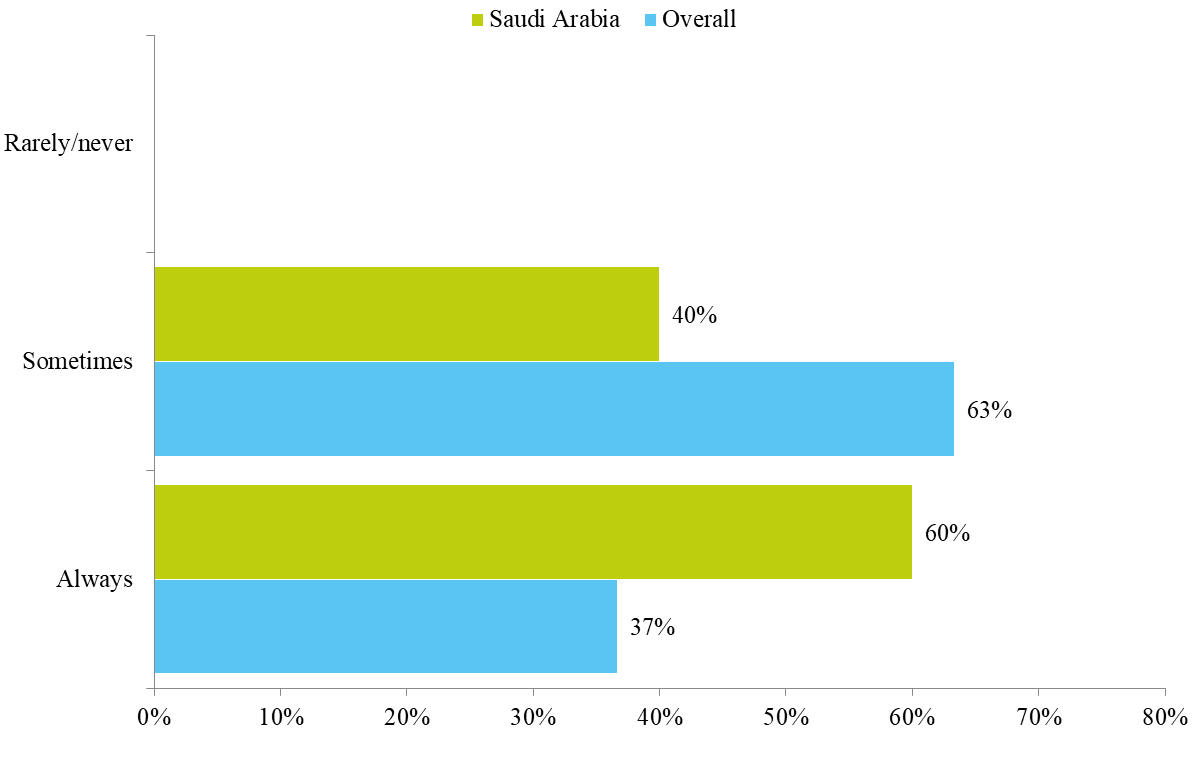


Thinking about antibiotics and the impact they can have on the microbiome, how would you respond to the following statements?


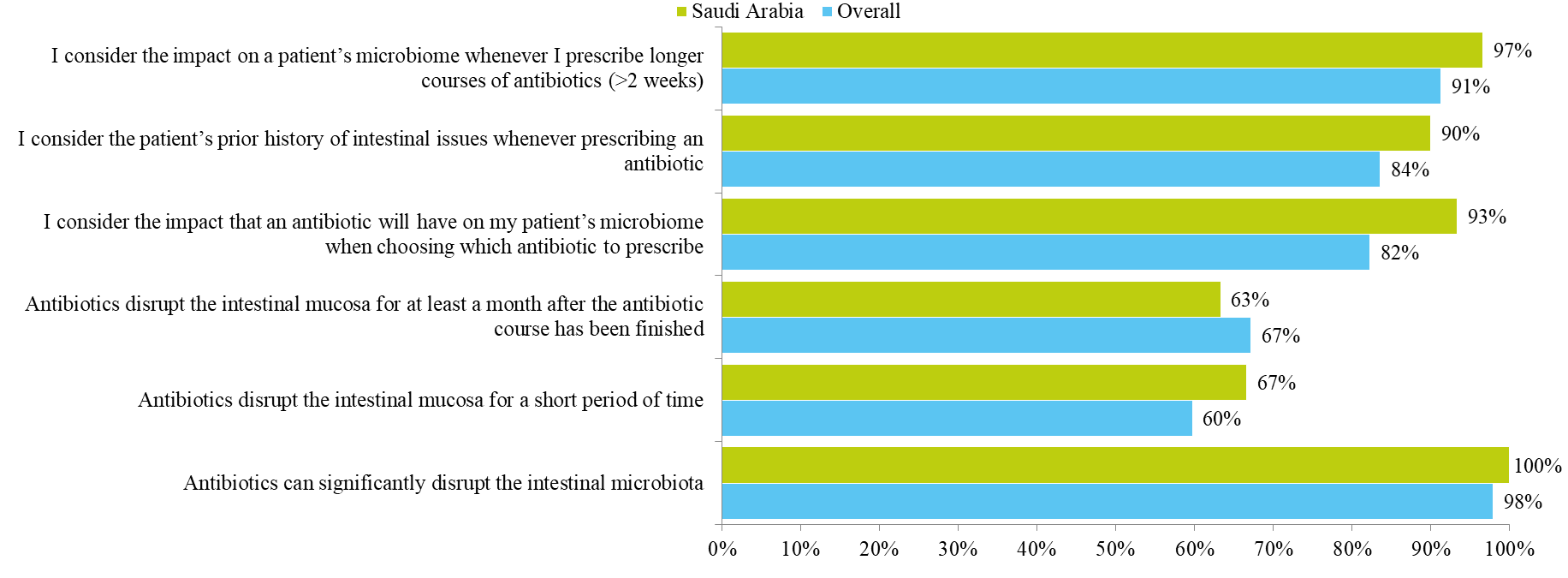


Thinking about antibiotics and the impact they can have on the microbiome, how do you respond to the following statements about probiotics?


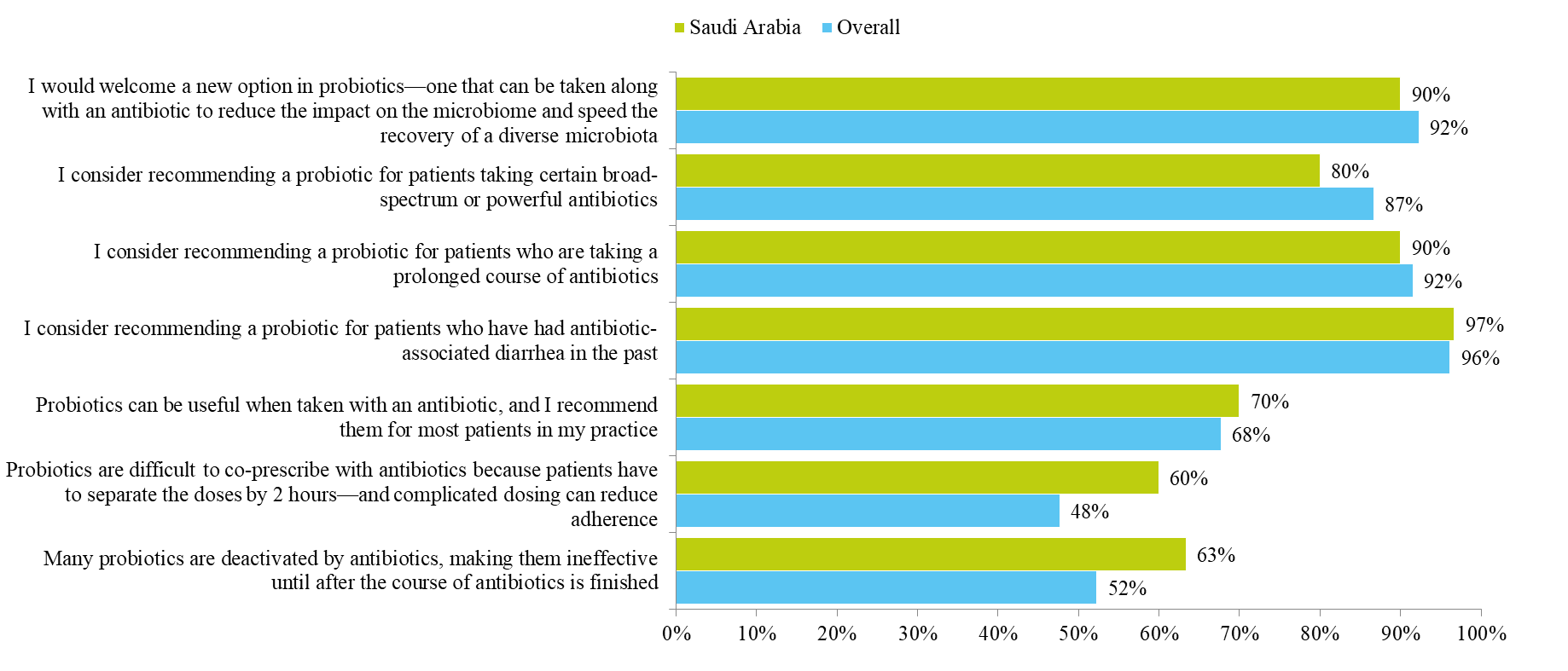


When prescribing antibiotics to adult patients today, for what percentage of patients do you also recommend probiotics?


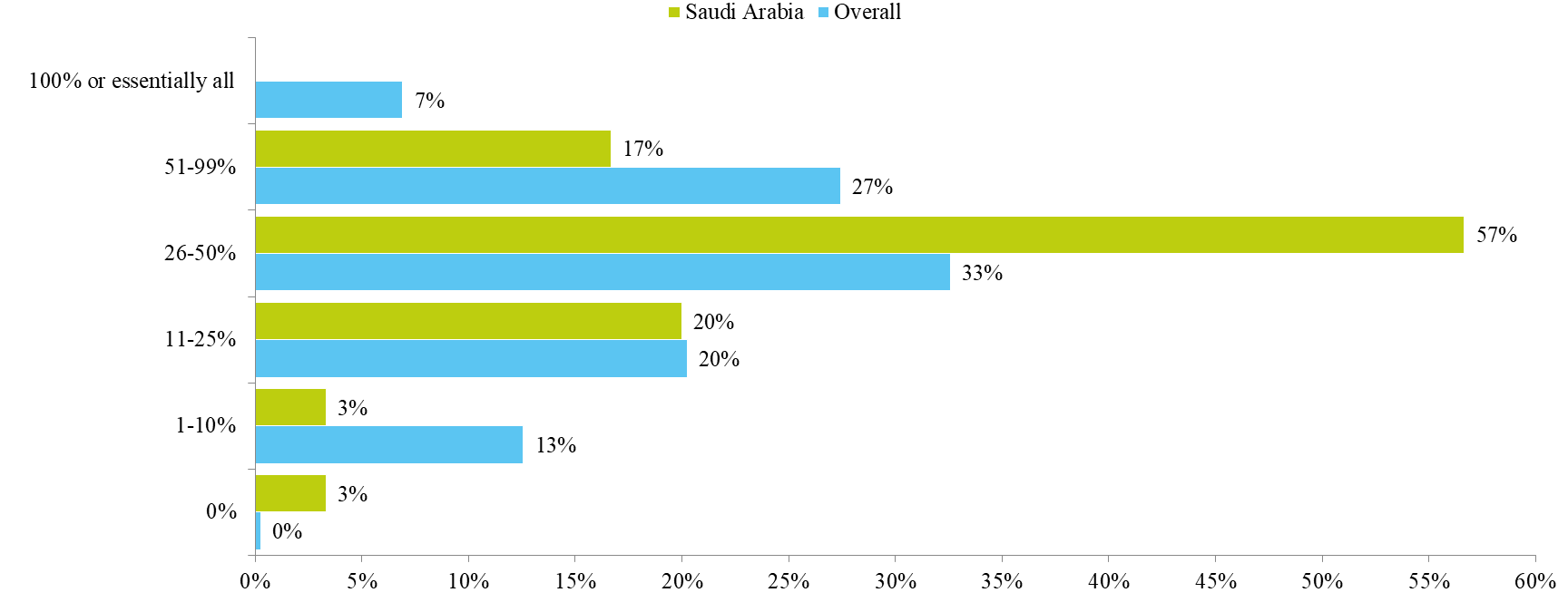


How would you respond to the following statements?


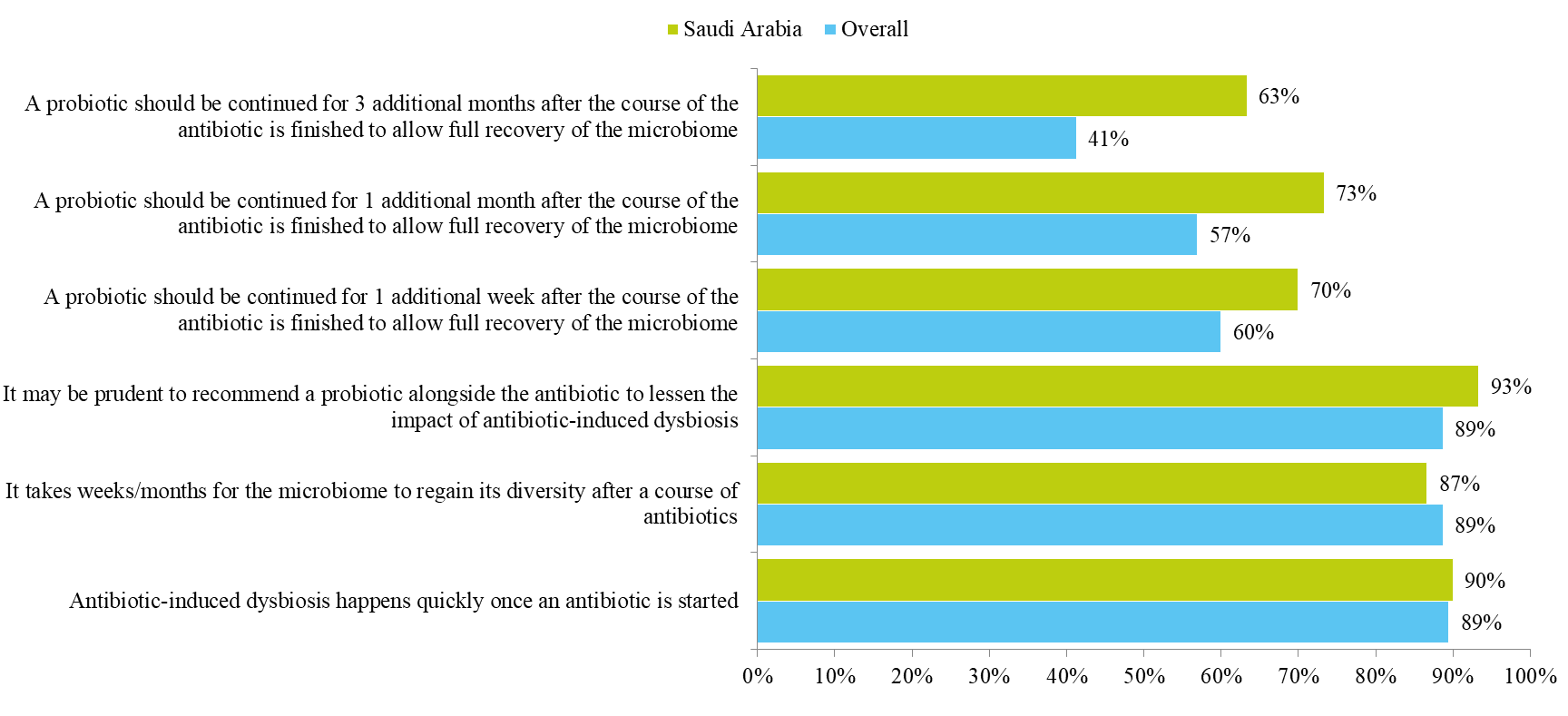


How would you respond to the following statements?


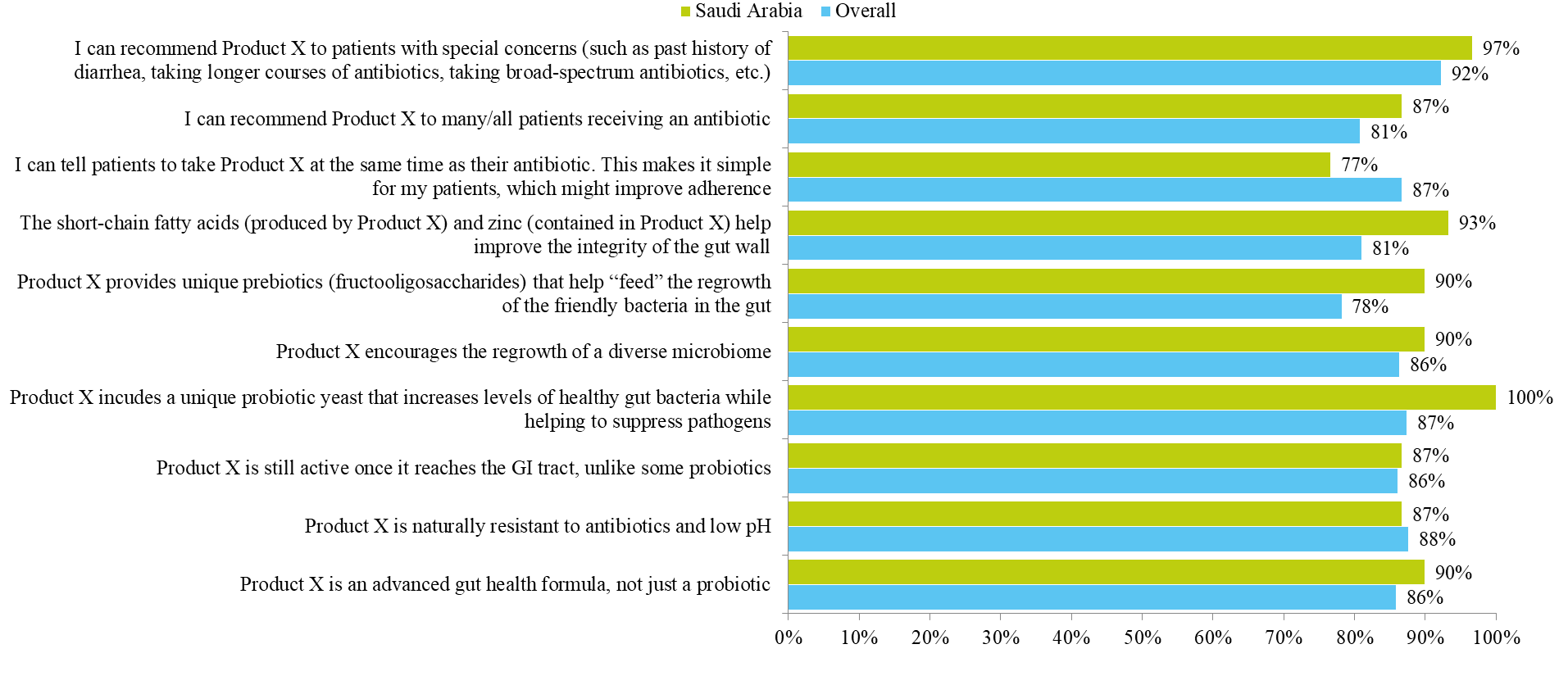


# South Africa Report

In a typical week during cold and flu season, what percentage of the adult patients you see receive an antibiotic?


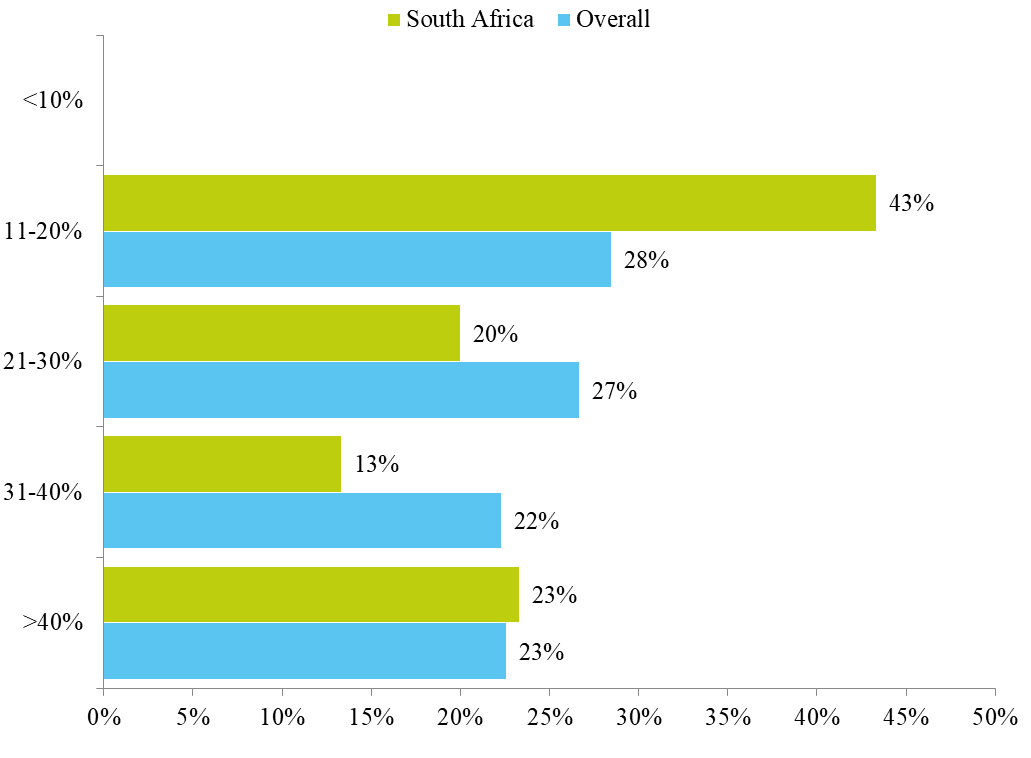


In a typical week, not during cold and flu season, what percentage of the adult patients you see receive an antibiotic?


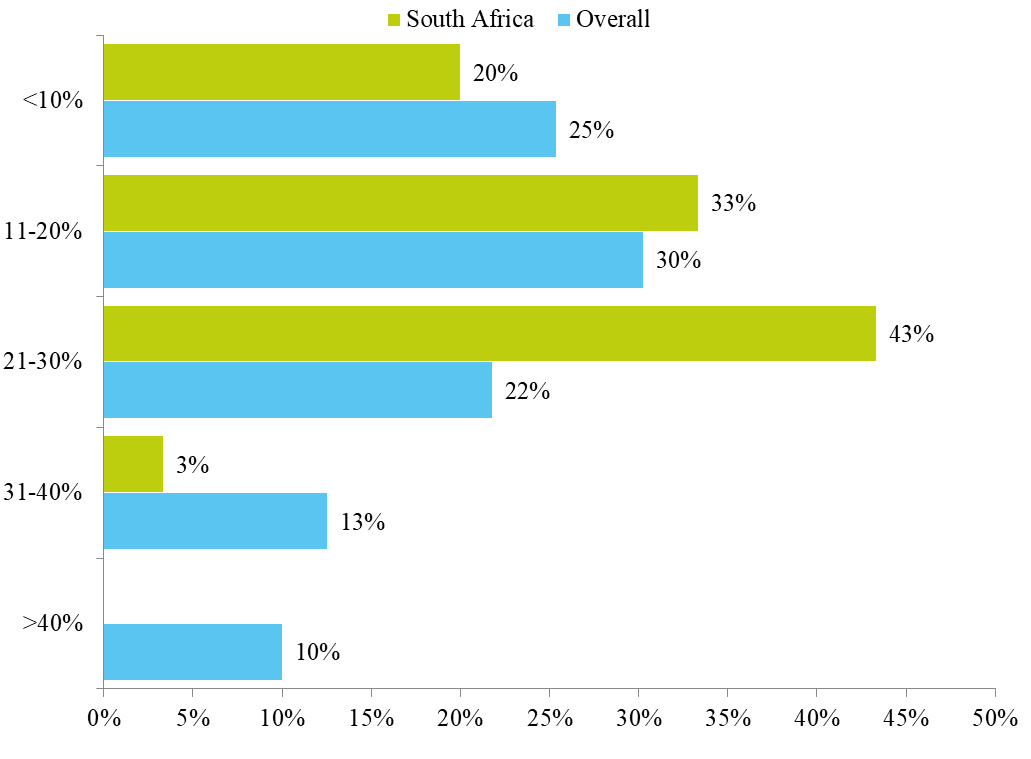


Do you see a role for probiotics when prescribing antibiotics to adult patients?


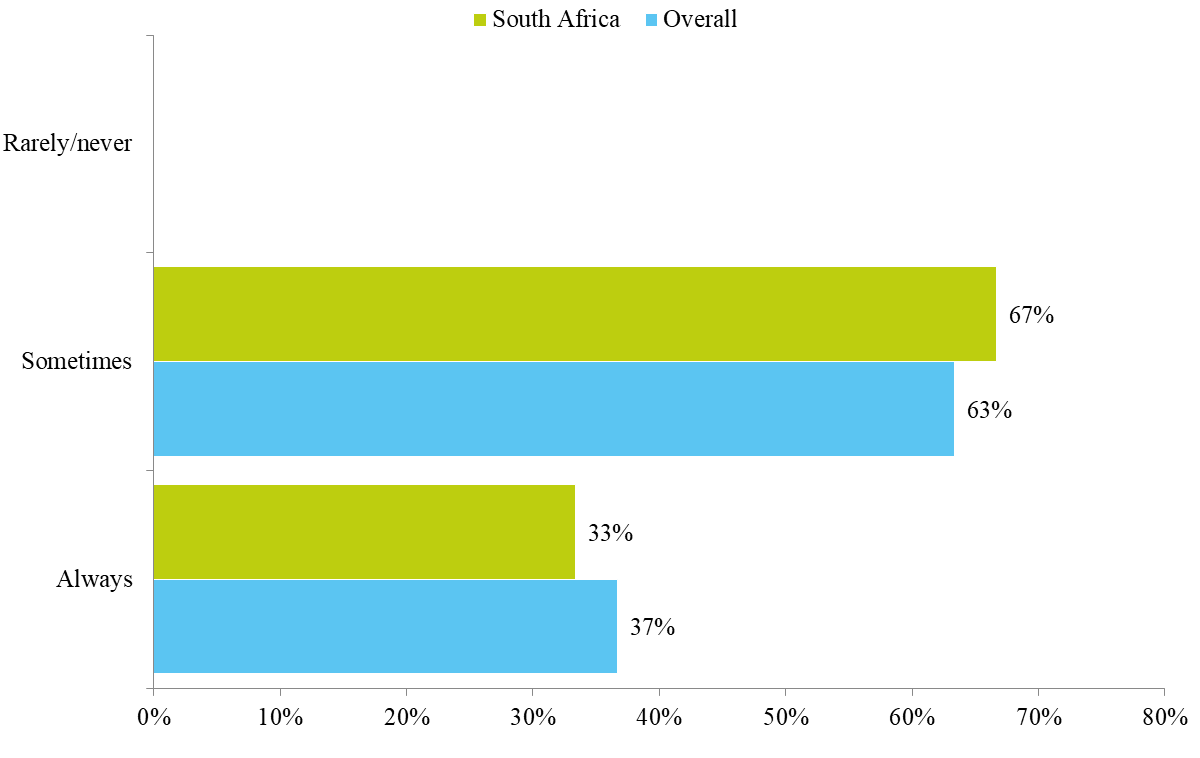


Thinking about antibiotics and the impact they can have on the microbiome, how would you respond to the following statements?


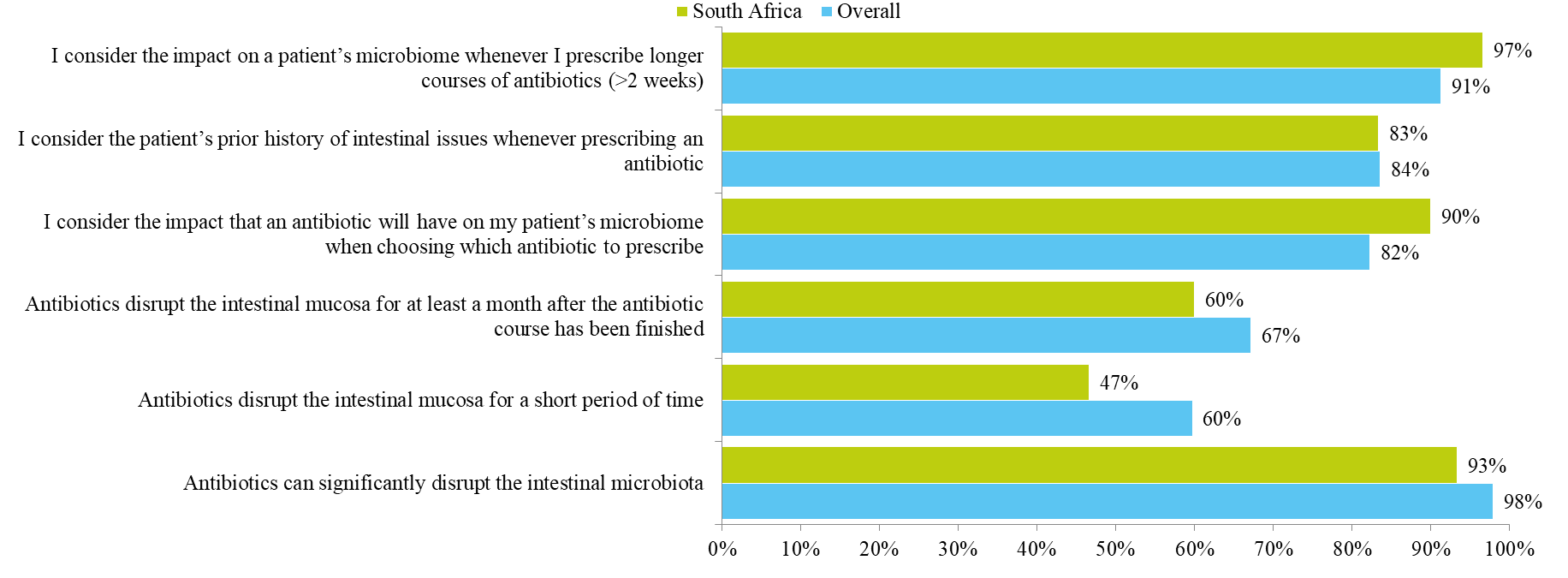


Thinking about antibiotics and the impact they can have on the microbiome, how do you respond to the following statements about probiotics?


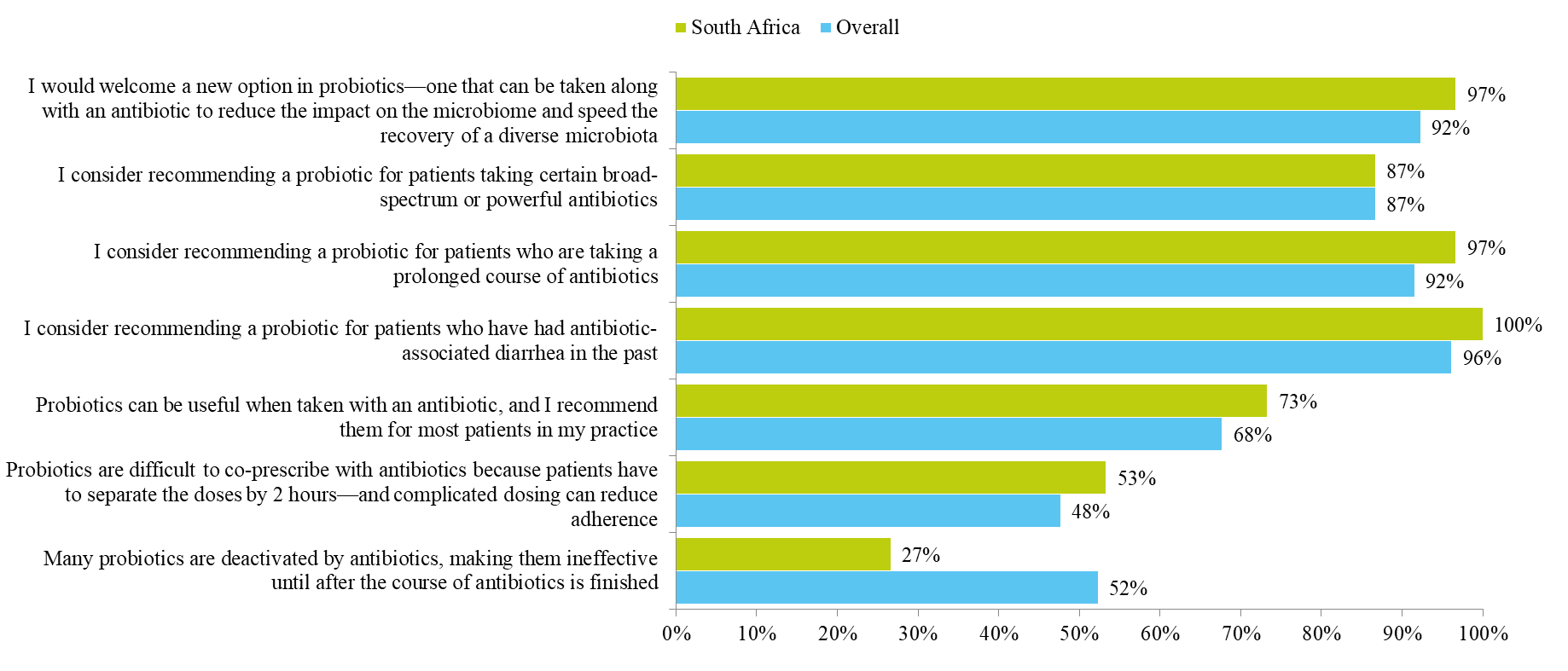


When prescribing antibiotics to adult patients today, for what percentage of patients do you also recommend probiotics?


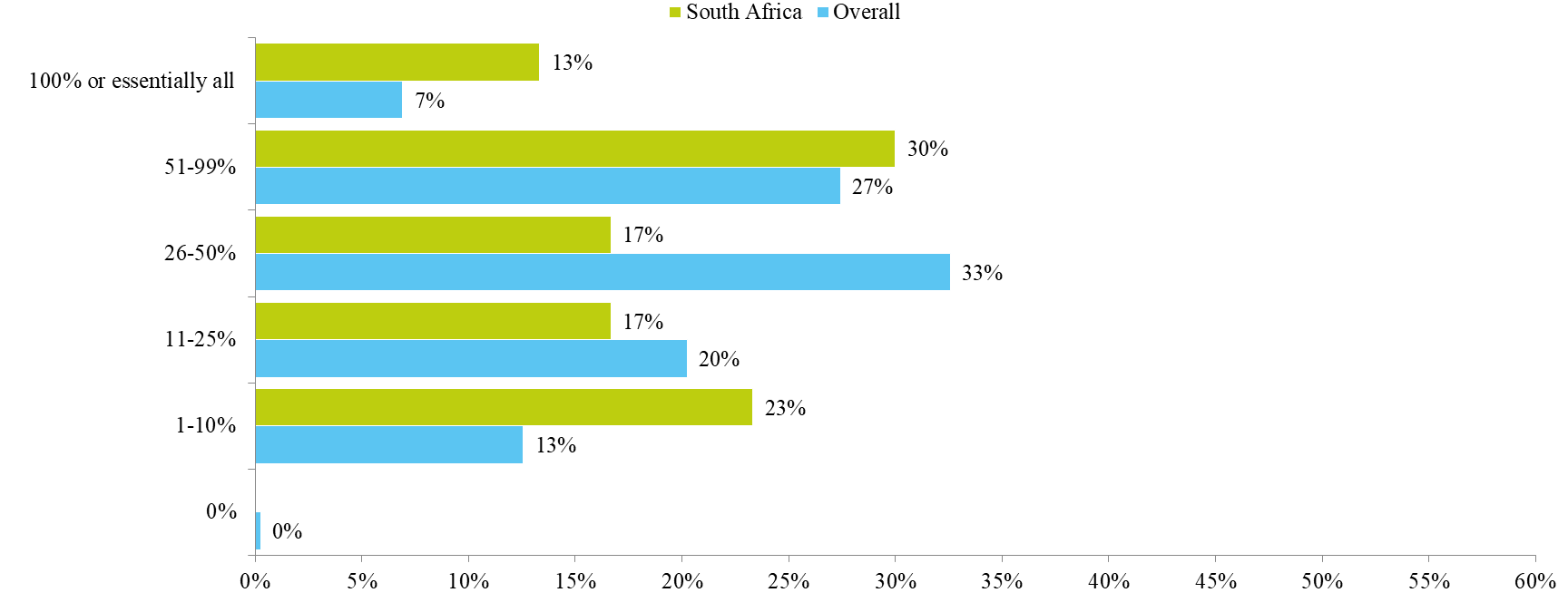


How would you respond to the following statements?


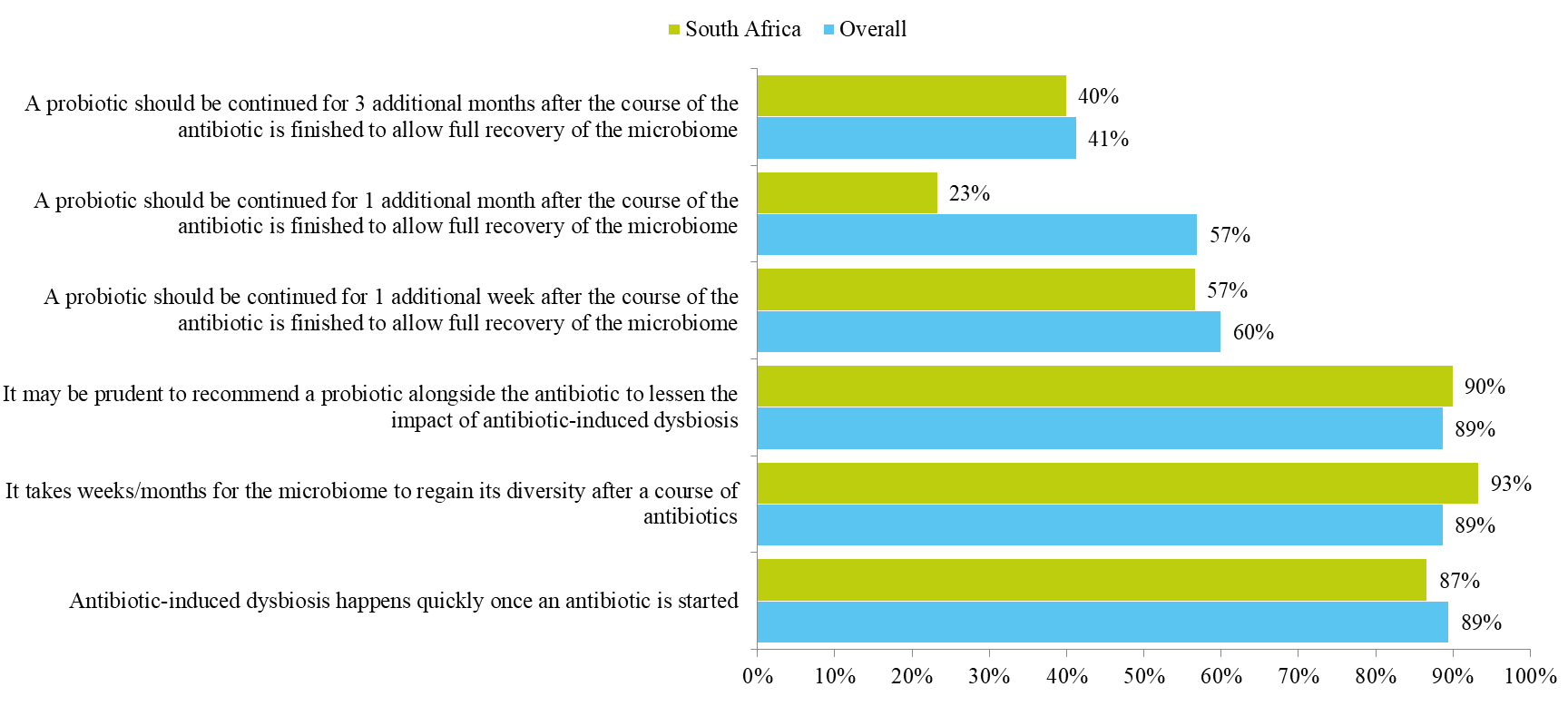


How would you respond to the following statements?


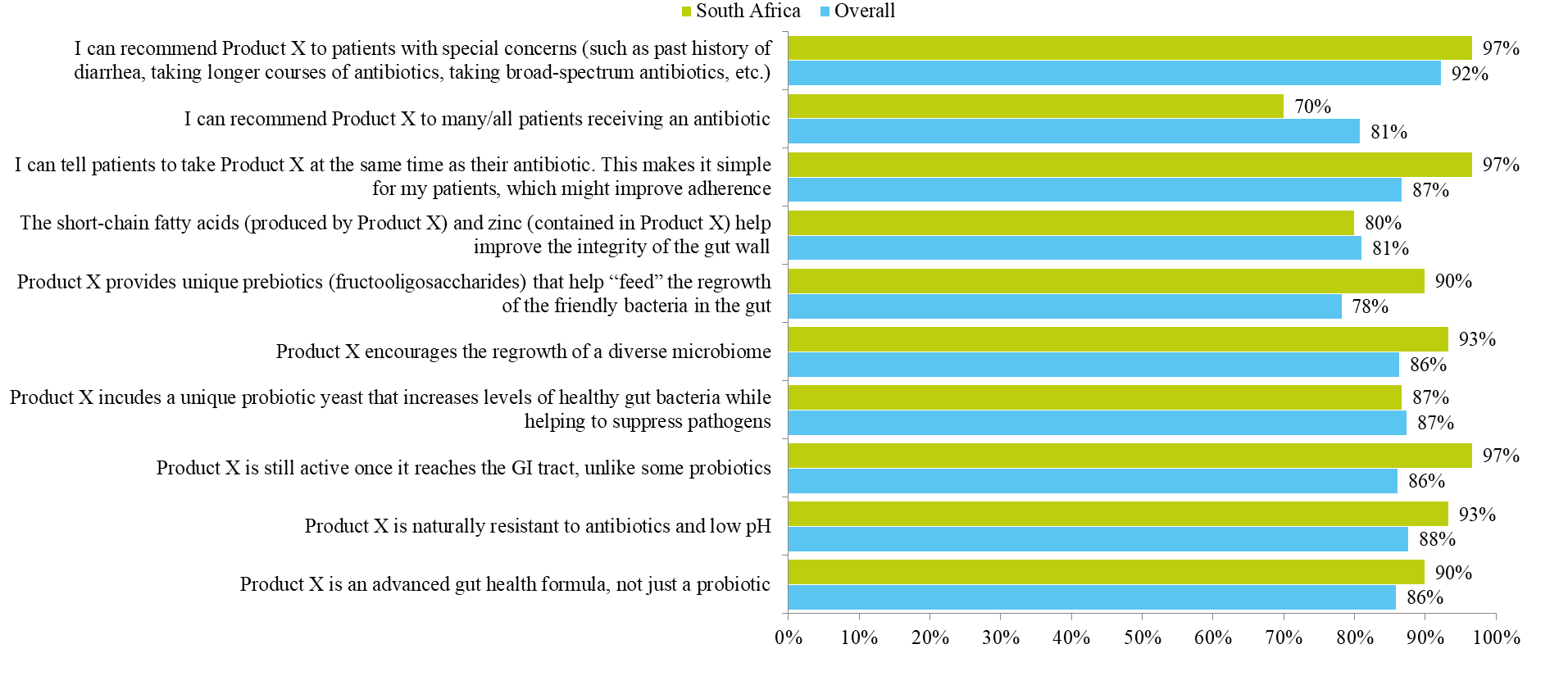


# Spain Report

In a typical week during cold and flu season, what percentage of the adult patients you see receive an antibiotic?


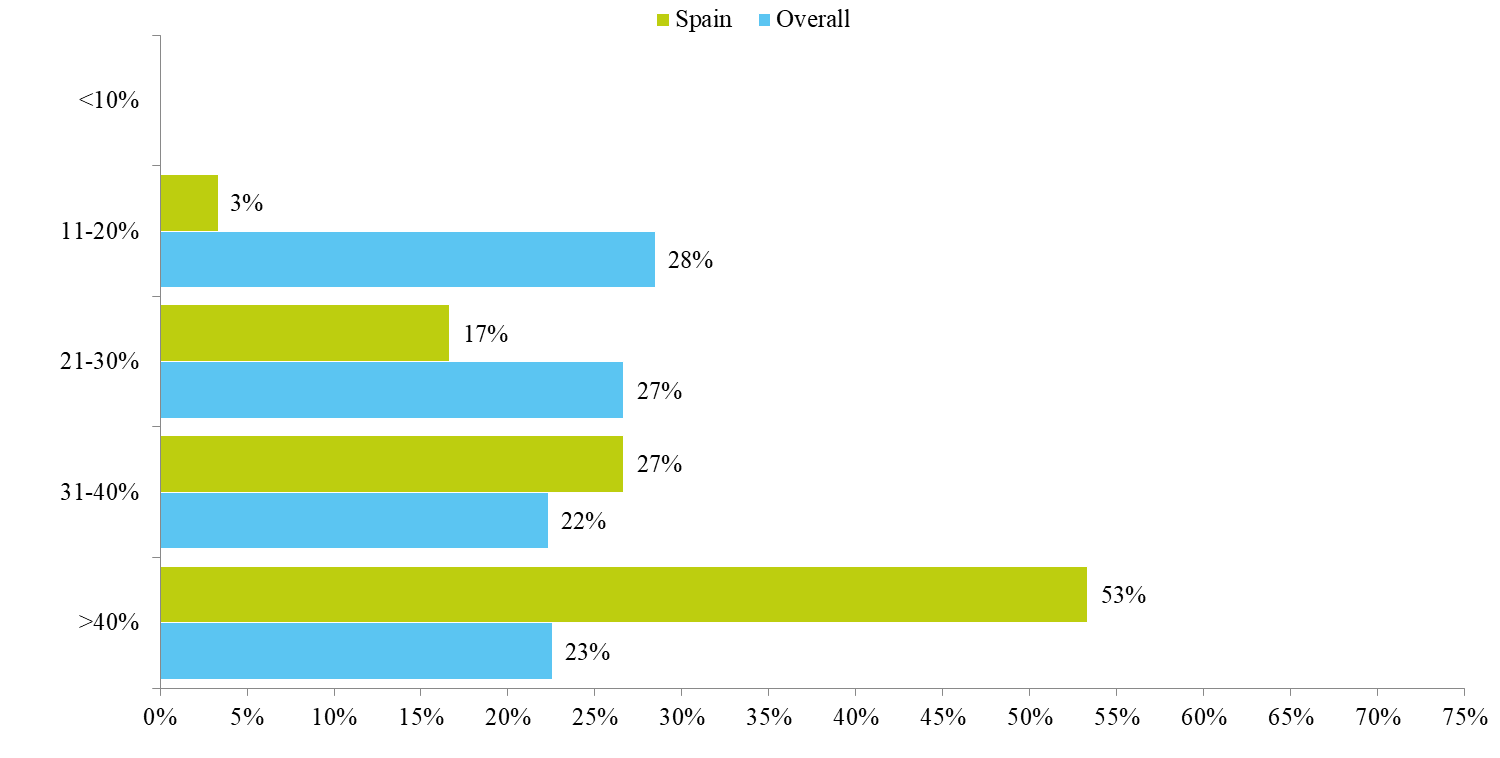


In a typical week, not during cold and flu season, what percentage of the adult patients you see receive an antibiotic?


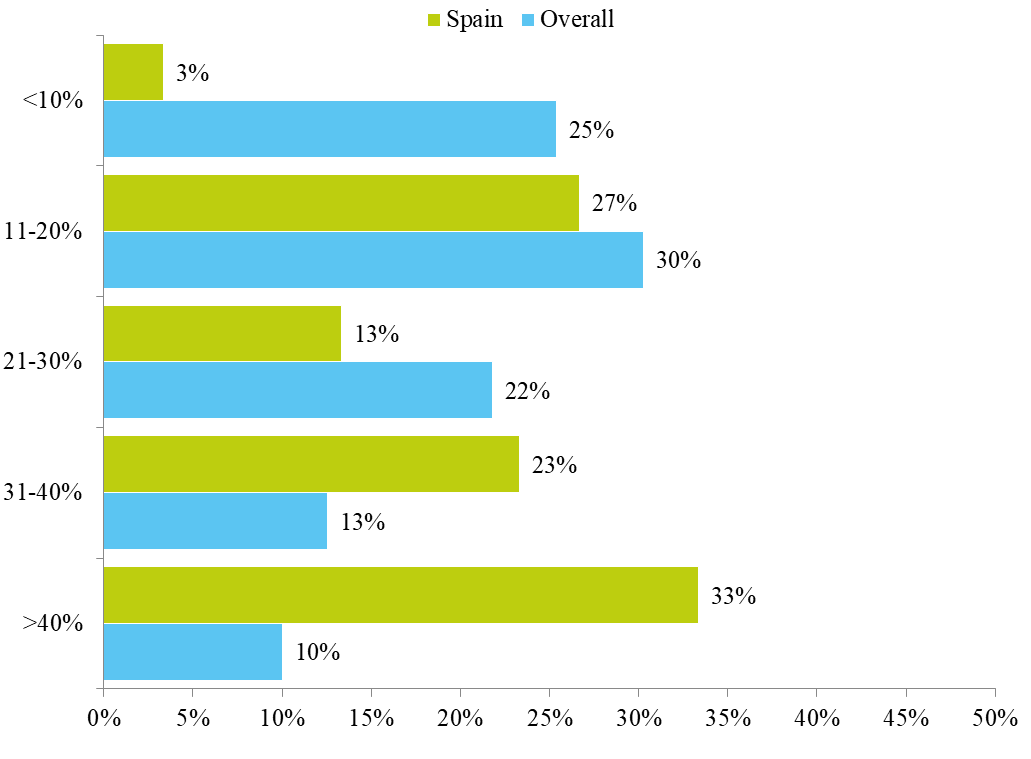


Do you see a role for probiotics when prescribing antibiotics to adult patients?


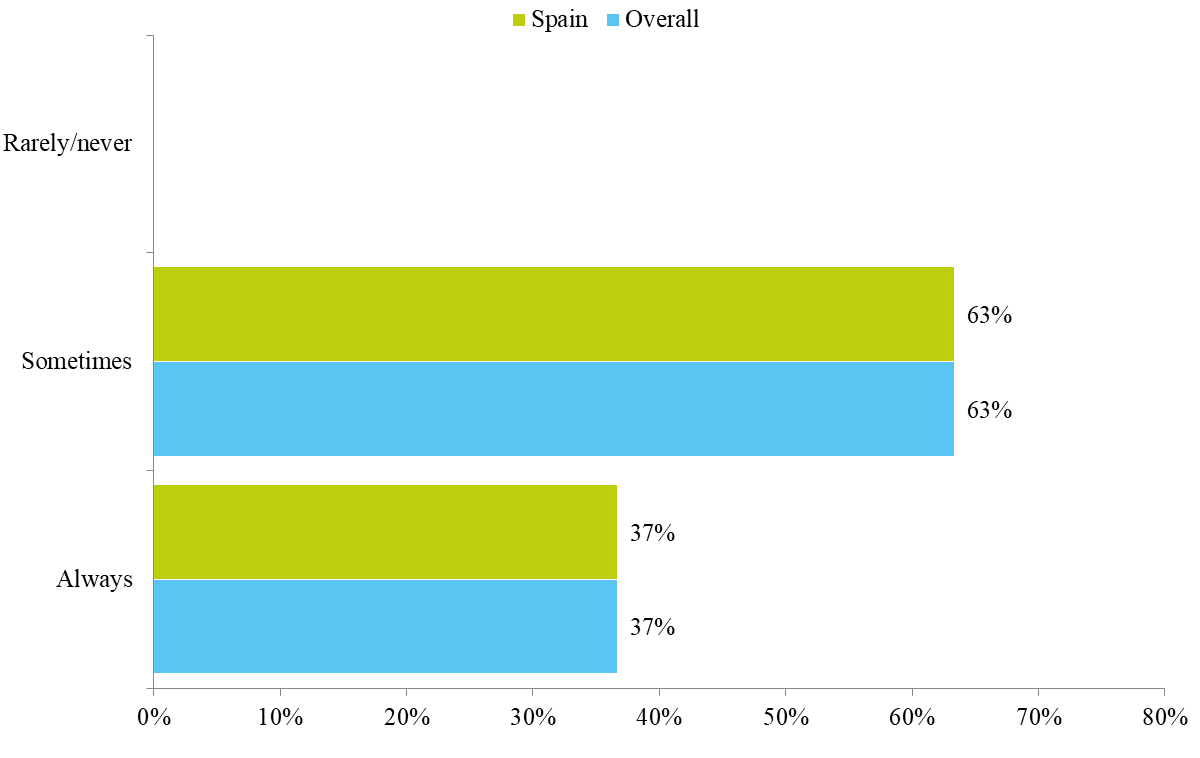


Thinking about antibiotics and the impact they can have on the microbiome, how would you respond to the following statements?


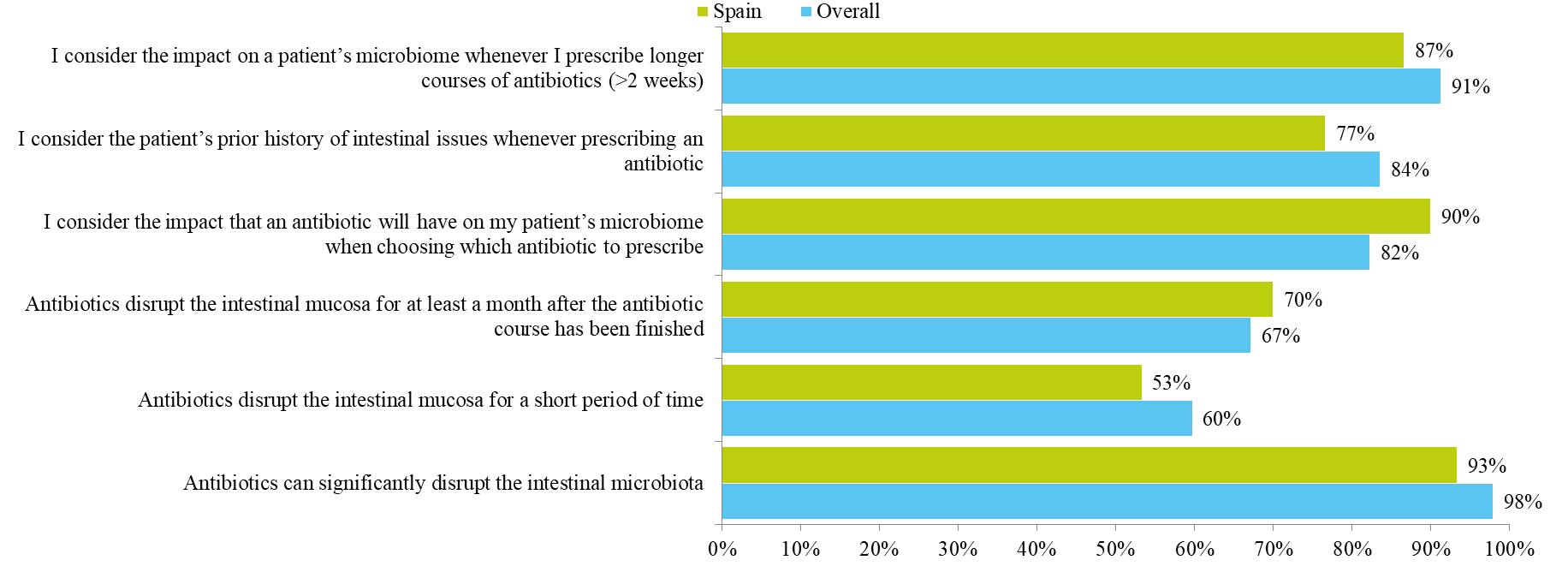


Thinking about antibiotics and the impact they can have on the microbiome, how do you respond to the following statements about probiotics?


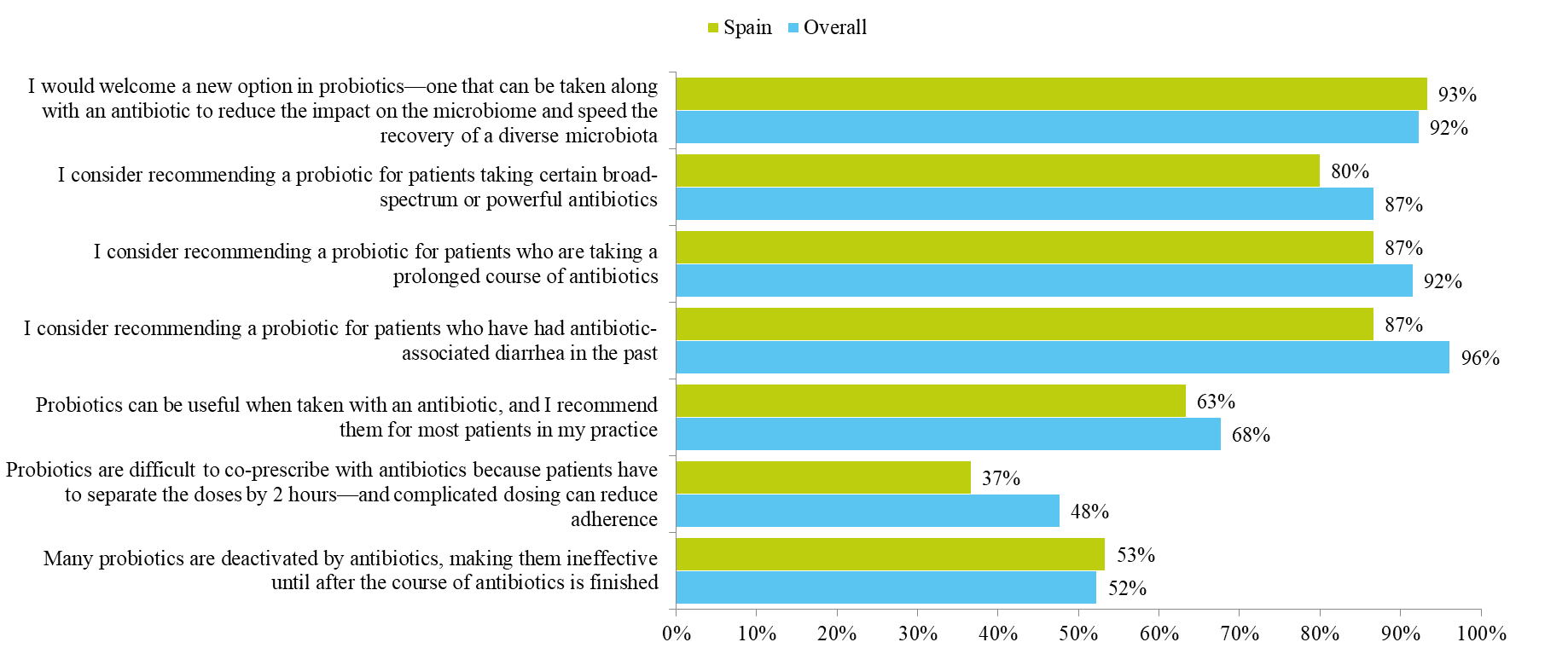


When prescribing antibiotics to adult patients today, for what percentage of patients do you also recommend probiotics?

How would you respond to the following statements?

How would you respond to the following statements?

# United Arab Emirates Report

In a typical week during cold and flu season, what percentage of the adult patients you see receive an antibiotic?

In a typical week, not during cold and flu season, what percentage of the adult patients you see receive an antibiotic?

Do you see a role for probiotics when prescribing antibiotics to adult patients?

Thinking about antibiotics and the impact they can have on the microbiome, how would you respond to the following statements?

Thinking about antibiotics and the impact they can have on the microbiome, how do you respond to the following statements about probiotics?

When prescribing antibiotics to adult patients today, for what percentage of patients do you also recommend probiotics?

How would you respond to the following statements?

How would you respond to the following statements?
